# Comparative transcriptome in large-scale human and cattle populations

**DOI:** 10.1186/s13059-022-02745-4

**Published:** 2022-08-22

**Authors:** Yuelin Yao, Shuli Liu, Charley Xia, Yahui Gao, Zhangyuan Pan, Oriol Canela-Xandri, Ava Khamseh, Konrad Rawlik, Sheng Wang, Bingjie Li, Yi Zhang, Erola Pairo-Castineira, Kenton D’Mellow, Xiujin Li, Ze Yan, Cong-jun Li, Ying Yu, Shengli Zhang, Li Ma, John B. Cole, Pablo J. Ross, Huaijun Zhou, Chris Haley, George E. Liu, Lingzhao Fang, Albert Tenesa

**Affiliations:** 1 MRC Human Genetics Unit at the Institute of Genetics and Cancer, The University of Edinburgh, EH4 2XU Edinburgh, UK; 2 School of Informatics, The University of Edinburgh, Edinburgh, EH8 9AB UK; 3 Animal Genomics and Improvement Laboratory, Henry A. Wallace Beltsville Agricultural Research Center, Agricultural Research Service, USDA, Beltsville, Maryland 20705 USA; 4 College of Animal Science and Technology, China Agricultural University, Beijing, 100193 China; 5 The Roslin Institute, Royal (Dick) School of Veterinary Studies, The University of Edinburgh, Midlothian, EH25 9RG UK; 6 Department of Psychology, 7 George Square, The University of Edinburgh, Edinburgh, EH8 9JZ UK; 7 Department of Animal and Avian Sciences, University of Maryland, College Park, MA 20742 USA; 8 Department of Animal Science, University of California, Davis, CA 95616 USA; 9 Present address: Institute of Animal Science, Chinese Academy of Agricultural Sciences, Beijing, China; 10 State Key Laboratory of Genetic Resources and Evolution, Kunming Institute of Zoology, Chinese Academy of Sciences, Kunming, 650223 Yunnan China; 11 Scotland’s Rural College (SRUC), Roslin Institute Building, Midlothian, EH25 9RG UK; 12 Guangdong Provincial Key Laboratory of Waterfowl Healthy Breeding, College of Animal Science & Technology, Zhongkai University of Agriculture and Engineering, Guangzhou, 510225 Guangdong China; 13 Present address: Center for Quantitative Genetics and Genomics, Aarhus University, Aarhus, Denmark

**Keywords:** Comparative transcriptome, Gene co-expression, Heritability enrichment, Inter-individual variability, RNA-seq

## Abstract

**Background:**

Cross-species comparison of transcriptomes is important for elucidating evolutionary molecular mechanisms underpinning phenotypic variation between and within species, yet to date it has been essentially limited to model organisms with relatively small sample sizes.

**Results:**

Here, we systematically analyze and compare 10,830 and 4866 publicly available RNA-seq samples in humans and cattle, respectively, representing 20 common tissues. Focusing on 17,315 orthologous genes, we demonstrate that mean/median gene expression, inter-individual variation of expression, expression quantitative trait loci, and gene co-expression networks are generally conserved between humans and cattle. By examining large-scale genome-wide association studies for 46 human traits (average *n* = 327,973) and 45 cattle traits (average *n* = 24,635), we reveal that the heritability of complex traits in both species is significantly more enriched in transcriptionally conserved than diverged genes across tissues.

**Conclusions:**

In summary, our study provides a comprehensive comparison of transcriptomes between humans and cattle, which might help decipher the genetic and evolutionary basis of complex traits in both species.

**Supplementary Information:**

The online version contains supplementary material available at 10.1186/s13059-022-02745-4.

## Background

Cross-species comparison of the transcriptome enables a better interpretation of how natural selection shapes gene expression and is crucial for exploring the evolutionary basis of phenotypic variation between and within species. Comparison of the transcriptome between human and mouse has enhanced the use of mouse as models for a wide variety of diseases including neurological and muscular disorders, as well as cancer [1]. Additionally, the comparison of the transcriptome across primates has provided molecular insights into human evolution, particularly in the brain [2].

Previous studies on comparative transcriptomics were essentially restricted to model organisms and human data from a few individuals, hindering the comparison of inter-individual variation of gene expression and associated genetic regulatory effects (e.g., expression quantitative trait loci, eQTLs) across species. Moreover, although it has been suggested that the genetic architecture underlying complex traits is conserved at a certain degree between humans and livestock [3–5], the molecular mechanisms underpinning such conservation are largely unknown. Until now, no study has systematically explored the conservation of transcriptome across a wide range of tissues in large populations of humans and any livestock species.

Cattle is one of the most economically important livestock species, supplying humans with a substantial fraction of animal protein. Driven by the high selection intensity of economically important traits, compared to humans, cattle has a different population structure, such as smaller effective population size (Ne ~100), higher linkage disequilibrium (LD) among genomic variants, and higher inbreeding rate (i.e., resulting in the accumulation of deleterious mutations) [6]. Furthermore, millions of highly accurate phenotypic records, including fertility, health, and growth traits, have been collected for cattle [7, 8]. As such, a better understanding of transcriptome conservation between humans and cattle may not only contribute to establishing cattle as a potential biomedical model for certain human diseases, but also enhance the cattle genetic improvement program by leveraging prior information from humans [5, 9]. Here, we select 10,830 and 4866 high-quality RNA-seq profiles from the human GTEx project (v8) [10] and the CattleGTEx project [11], respectively. We group human samples from similar tissues (e.g., different brain regions as brain) into bigger tissue classes, resulting in 20 matched tissues in humans and cattle (Additional file 1: Table S1). The large and tissue-diverse dataset analyzed allowed us to systematically compare the transcriptome of humans and a livestock species to gauge the conservation of gene expression in two outbred mammalian populations. We compare mean gene expression, inter-individual variation of gene expression, *cis*-eQTLs, and co-expression networks between humans and cattle, and then integrate results with large-scale genome-wide association studies (GWAS) from 46 human traits and 45 cattle traits to understand the genetic and evolutionary basis of complex traits.

## Results

### Global conservation of gene expression

**Fig. 1:**
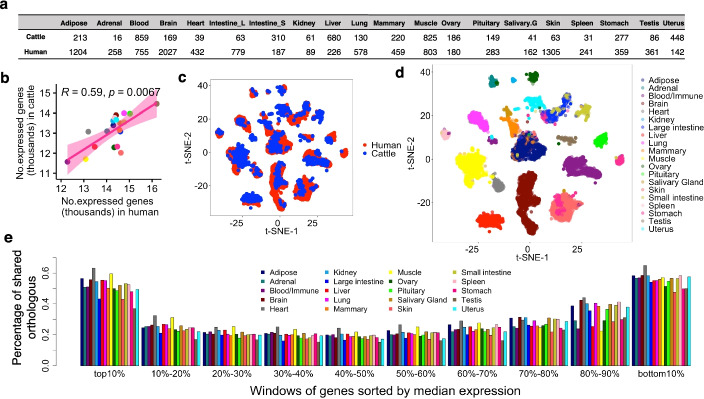
Data summary and conservation of transcriptomes of 20 common tissues in humans and cattle. **a** Sample size per tissue in humans and cattle. **b** Spearman’s correlation of number of expressed genes (median TPM > 0.1) across tissues between humans and cattle. Each dot represents a tissue. **c** Plot of *t*-SNE of samples based on batch-corrected gene expression (Methods). Each dot represents a sample, colored by species types. **d** Same as in **c**, but colored by tissue types. **e** Percentage of orthologous genes shared in each window between humans and cattle. Genes were ranked (from largest to smallest) by median expression in each tissue each species, and then divided into ten windows evenly (1731 genes per window)

We focused on the expression of 17,315 one-to-one orthologous genes, including 72% and 76% of all annotated protein-coding genes in humans and cattle, respectively. These orthologous genes, representing 16,510 protein-coding genes with 664 on sex chromosome, contributed to the majority of transcriptional outputs among all 20 tissues being studied in both humans and cattle (Additional file 2: Fig. S1). We analyzed an average of 243 and 541 RNA-seq samples across these 20 tissues in cattle and humans, respectively (Fig. 1a, Additional file 1: Table S1). We observed a significant correlation (Spearman’s *r* = 0.59, *p* = 6.7×10^−3^) between the number of expressed (median Transcripts per Kilobase Million, TPM > 0.1) genes in each tissue in humans and cattle (Fig. 1b). Testis has the largest number of expressed genes in both species (*n*_Human_ = 16,204; *n*_Cattle_ = 14,457), while muscle (*n*_Human_ = 13,081; *n*_Cattle_ = 11,707) and blood (*n*_Human_ = 12,283; *n*_Cattle_ = 11,573) have the smallest in cattle and humans, respectively.

The *t*-SNE-based visualization of expression variation among samples clearly recapitulated tissue types (Fig. 1c, d). The hierarchical clustering of tissues based on mean or median gene expression in each tissue also showed that tissues rather than species clustered together (Additional file 2: Fig. S2a-b). These results demonstrate that gene expression profiles of orthologous genes are generally conserved within corresponding tissues between cattle and humans (Additional file 2: Fig. S3a). Tissues with the highest similarity of gene expression between humans and cattle included brain, pituitary, muscle, and adipose, while tissues with the lowest included stomach (the majority were rumen in cattle), skin, testis, and mammary gland (Additional file 2: Fig. S3b). In addition, we sorted all orthologous genes according to their median level of expression in each tissue, and observed that humans and cattle share most genes in the top (highest expression) and bottom (lowest expression) 10% of genes (Fig. 1e).

### Conservation of tissue specificity of gene expression

**Fig. 2:**
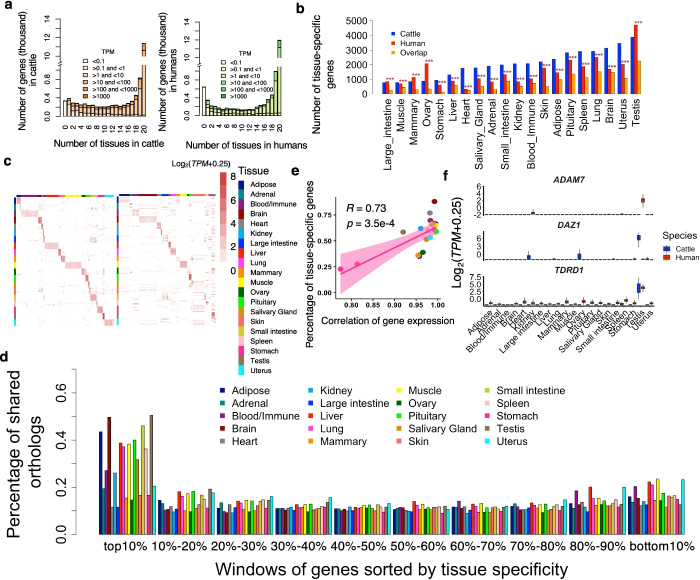
Comparison of tissue specificity of gene expression. **a** Gene expression levels and number of tissues in which genes were expressed (median TPM > 0.1) in cattle (left) and humans (right). **b** Number of tissue-specific genes (log_2_(fold-change) > 1.5 and FDR < 0.05) and their overlap across 20 tissues in humans and cattle. The overlap was tested using hypergeometric test. “***” represents FDR (Benjamini-Hochberg method corrected *P*-value) less than 1.0×10^−3^. **c** Expression profiles of top 10 tissue-specific genes that are detected in cattle among both cattle (left) and humans samples (right). Each row represents a gene and each column represents a sample from the corresponding tissue. The color represents log_2_-transformed expression value, i.e., log_2_(TPM+0.25). **d** Percentage of orthologous genes shared in each bin between humans and cattle. Genes were ranked (from largest to smallest) by degree (measured by −log_10_*p*) of tissue specificity, and then divided into ten bins (1731 genes per bin). **e** Spearman’s correlation between the percentage (%) of overlapping tissue-specific genes and gene expression correlation between humans and cattle across 20 tissues. Each dot represents a tissue. **f** Expression profiles of *ADAM7* (human-specific testis gene), *DAZ1* (cattle-specific testis gene), and *TDRT1* (conserved testis gene)

We found that the distribution of median gene expression across tissues was U-shaped (tending towards either tissue-specific or ubiquitously expressed) in both humans and cattle, with the majority of genes (69% and 66% in humans and cattle, respectively) expressed in all 20 tissues (Fig. 2a). The number of tissues in which each gene was expressed was significantly correlated between the two species (Spearman’s *r* = 0.75, *p* < 2.2×10^−16^), indicating that among orthologous genes there is global conservation of tissue-specific expression between humans and cattle. We found that 639 and 337 genes, with a significant (Hypergeometric test, *p* < 2.2×10^−16^) overlap of 165, were not measurably expressed (TPM < 0.1) at the time of measurement in any of 20 tissues in humans and cattle, respectively. These non-expressed genes were significantly enriched in embryonic development processes, such as embryonic morphogenesis, angiogenesis, and regulation of stem cell division (Additional file 2: Fig. S4a). This might be due to the underrepresentation of embryonic samples in the current study.

We found that the number of tissue-specific genes across tissues was significantly correlated (Spearman’s *r* = 0.68, *p* = 1.2×10^−3^) between humans and cattle (Additional file 2: Fig. S4b). The testis had the largest number of tissue-specific genes, while the large intestine and heart had the smallest in cattle and humans, respectively. In general, tissue-specific genes of the same tissues overlapped significantly (Hypergeometric test, FDR < 1.0×10^−3^) between humans and cattle (Fig. 2b). In each tissue, the top 10 tissue-specific genes with the largest expression values detected in cattle tissues also exhibited a strong pattern of tissue-specific expression in human tissues (Fig. 2c), and vice versa for the top 10 tissue-specific genes detected in human tissues (Additional file 2: Fig. S4c). We observed that tissue specificity in gene expression was linked to the chances of genes being transcriptionally conserved between humans and cattle (Fig. 2d). The more similar the expression of two tissues was between species the larger the number of shared tissue-specific genes the tissues had (Spearman’s *r* = 0.73; *p* = 3.5×10^−4^) (Fig. 2e). This finding indicates that tissues with more tissue-specific genes shared between humans and cattle tend to be more transcriptionally conserved between these two species.

We found that the tissue-specific genes shared by species (conserved) accurately reflected the known biology of tissues, while tissue-specific genes that were not shared by species (diverged) showed distinct biological functions in humans and cattle (Additional file 3: Table S2). For instance, the conserved testis-specific genes were significantly engaged in germ cell development, while human-specific and cattle-specific ones were significantly engaged in cilium organization and synapse assembly, respectively (Additional file 2: Fig. S4d). Of note, the difference in gene annotation databases between humans and cattle might bias the biological interpretation of human- and cattle-specific genes. We took *ADAM7*, *DAZ1*, and *TDRD1* as examples of human-specific, cattle-specific, and conserved genes in testis (Fig. 2f). *ADAM7* plays roles in sperm maturation and sperm-egg fusion [12]. *DAZ1* and *TDRD1* are essential for spermatogenesis [13, 14]. These species-specific genes in testis might be linked to the difference in fertility between humans and cattle, e.g., the difference in embryo implantation [15].

### Comparison of mean gene expression level

**Fig. 3:**
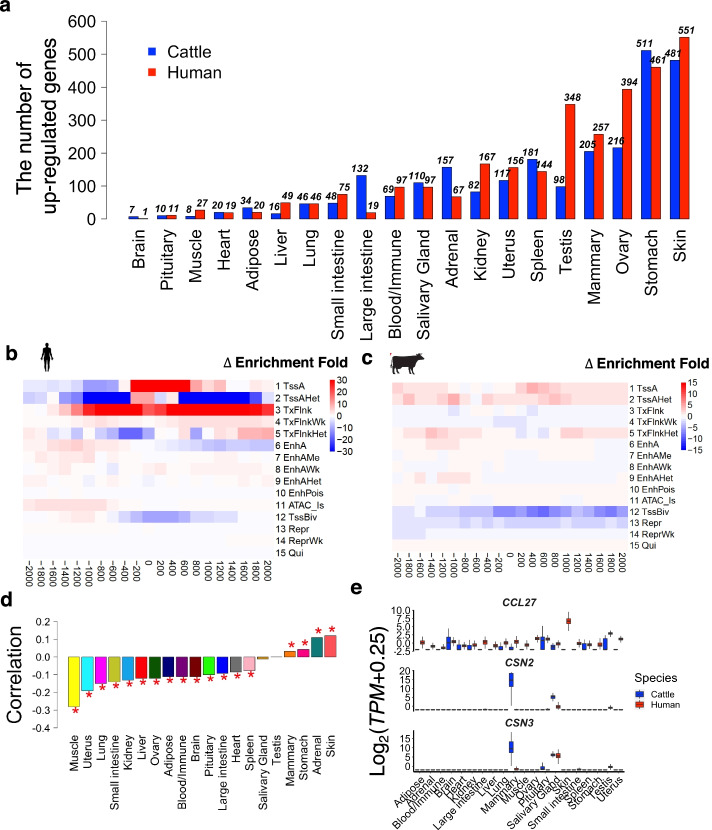
Comparison of average gene expression across 20 tissues between humans and cattle. **a** Number of significantly upregulated genes across tissues in humans (red) and cattle (blue) using the cutoff of fold-change (FC) > 1.2 and FDR < 0.05. **b, c** Changes of enrichment folds of 15 chromatin states around (± 2kb) transcriptional start sites (TSS) of top 500 upregulated genes in human and cattle adipose when compared with each other, respectively. The 15 chromatin states are predicted based on six epigenetic marks (i.e., ATAC, CTCF, H3K27ac, H3k27me3, H3K4me1 and H3K4me3). **d** Spearman’s correlation of genes between their tissue specificity (measured by −log_10_*p* from tissue specificity expression analysis) of expression and degrees (−log_10_*p*) of differential expression between species. “*” represents the correlation coefficient is significant (FDR < 0.01). **e** Expression profiles of *CNS2*, *CNS3*, and *CCL27* across human (red) and cattle (blue) tissues

We identified differentially expressed genes (DEGs) in each tissue between humans and cattle (Additional file 2: Fig. S5), and found that brain and pituitary showed the lowest number of DEGs (Fig. 3a), consistent with previous report that the central neural system evolves slowly across mammals [16]. In contrast, skin and stomach had the greatest number of DEGs, which was in line with the distinct physiological and anatomical characteristics of skin and stomach between humans and cattle. Using independent epigenetic data (i.e., ATAC-seq, and ChiP-seq for H3K4me3, H3K4me1, H3k27ac, and H3K27me3) in six common tissues in humans and cattle, we predicted 15 distinct chromatin states (Additional file 2: Fig. S6). We furthermore confirmed that TSS ± 2kb of human upregulated DEGs showed an increased enrichment of active promoter-related states (e.g., TssA and TxFlnk ) and decreased enrichment of repression-related states (e.g., TssBiv, TssAHet, Repr, and ReprWk) in humans when compared to their orthologous genes in cattle, and vice versa for cattle upregulated DEGs (Fig. 3b,c, Additional file 2: Fig. S7a). Furthermore, the upregulated DEGs in either humans or cattle exhibited distinct biological functions (Additional file 2: Fig. S7b, Additional file 4: Table S3). For instance, genes that were upregulated in cattle mammary gland were significantly engaged in protein secretion regulation, while genes that were upregulated in the human mammary gland were significantly engaged in responses to oxygen level (Additional file 4: Table S3). The oxygen level is important for supporting the increased metabolic rate during pregnancy and lactation in mammary gland. The downregulation of these genes in cattle mammary gland compared to humans might be partially due to the intensive selection of milk production and mammary gland health traits (e.g., mastitis) in cattle. We detected 511 and 461 genes were up- and downregulated in cattle rumen compared to human stomach. The upregulated genes in cattle rumen were mainly enriched in multicellular organismal water homeostasis, cell-cell adhesion, and tissue development, while the downregulated genes were significantly enriched in digestion, response to topologically incorrect protein, response to endoplasmic reticulum stress, and muscle contraction. In addition, we detected 481 and 551 genes were up- and downregulated in cattle skin compared to human skin. The upregulated genes in cattle skin were mainly enriched in anatomical structure morphogenesis, vasculature development, blood vessel development, and inflammatory response, while the downregulated genes were significantly enriched in skin development, epidermis development, regulation of water loss via skin, establishment of skin barrier, and keratinocyte differentiation. However, further experimental follow-ups are required to understand how the differential expression of these genes reflects biological differences in corresponding tissue functions between humans and cattle.

To further explore whether the findings were consistent between humans and mice, we integrated 113 RNA-seq samples from 14 tissues in mice [17, 18]. We found that gene expression profiles of most of tissues were generally conserved among the three mammals (Additional file 2: Fig. S8a), and the differential expression of genes (measured by *t*-statistics) were significantly but moderately correlated between humans *vs*. cattle and humans *vs*. mice (Additional file 2: Fig. S8b-c). We then detected genes that showed conservation (|FC|< 1.2 and FDR > 0.05) in humans *vs*. cattle, but divergence (|FC| > 1.2 and FDR < 0.05) in humans *vs*. mice (Additional file 2: Fig. S8d). For instance, those genes in adipose, spleen, lung, and mammary gland were significantly enriched for immune systems, such as T cell activation and regulation of lymphocyte proliferation (Additional file 2: Fig. S8e, Additional file 5: Table S4). This might suggest that cattle show a greater similarity to humans than mice in terms of several aspects of immunophysiology, which was in agreement with previous studies that cattle is a preferred model for human immunology [19, 20]. We also noticed that those genes in heart and liver were significantly involved in muscle contraction, ATP processing, and glucose metabolism, which might be in line with that cattle has been proposed as a model for some muscular disorders, e.g., brody disease [21].

Furthermore, we found that the degree (measured by −log_10_*p*) of differential expression of genes between humans and cattle was significantly and negatively correlated with their tissue specificity of expression in most of the tissues within humans (Fig. 3d), suggesting that genes with higher tissue-specific expression are more likely to be transcriptionally conserved (i.e., less differentially expressed) between humans and cattle. However, this was not universal as the opposite trend was found in skin, adrenal, and stomach, suggesting that certain functions of such tissues might be under positive selection in humans and cattle [22]. In addition, we found that dN/dS ratios (measuring DNA sequence conservation) of orthologous genes were weekly but significantly with their Tau values (measuring tissue-specific expression) in humans and cattle (Additional file 2: Fig. S9). We then investigated 30 genes with dN/dS ratio > 1, considered as positively selected between humans and cattle. Among them, 26 showed tissue-specific expression, and 14 were also significantly differentially expressed in at least one tissue between humans and cattle (Additional file 2: Fig. S10). For instance, *CSN2* and *CSN3*, which are associated with milk production traits in cattle [8], were significantly upregulated in the cattle mammary gland compared to human mammary gland (Fig. 3e). *CCL27*, which participates in T cell-mediated skin inflammation [23], was highly expressed in human skin, but not in cattle skin (Fig. 3e).

### Comparison of inter-individual variation of gene expression and their cis-genetic regulatory effects

**Fig. 4:**
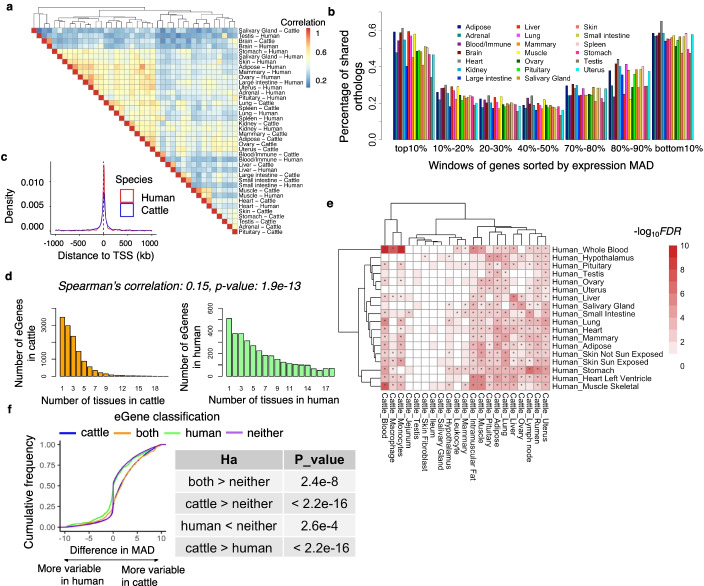
Comparison of inter-individual variability of gene expression and their *cis*-genetic regulatory effects. **a** Hierarchical clustering of tissues in humans and cattle based on Pearson’s correlation of median absolute deviation (MAD) of expression. **b** Percentage of orthologous genes shared in each bin between humans and cattle. Genes were ranked (from largest to smallest) by MAD, and then divided into ten bins (1731 genes per bin). **c** Distribution of top *cis*-eQTLs around transcriptional start sites (TSS) in human and cattle liver. **d** Number of eGenes (genes with significant *cis*-expression quantitative trait loci, *cis*-eQTLs) in what number of tissues in cattle (left) and humans (right). There is a weak but significant correlation (Spearman’s *r* =0.15; *p* = 1.91×10^−13^) between the number of tissues an eGene was detected on across both species **e**, Enrichment of eGenes between human and cattle tissues. Color represents −log_10_FDR. *P*-values are computed using the hypergeometric test for the overlaps of eGenes between human and cattle tissues, and then are adjusted for multiple testing with FDR method. “*” represents FDR < 0.05. **f** Distribution of difference in median absolute deviation (MAD) between humans and cattle among four groups of genes in blood, i.e., cattle-specific eGenes (cattle), human-specific eGenes (human), species-shared eGenes (both), and non-eGenes in neither species (neither)

Like mean gene expression levels, we found that the inter-individual variation of gene expression (measured by median absolute deviation, MAD) was generally conserved in humans and cattle (Fig. 4a). We then sorted all orthologous genes according to their level of variability and found that humans and cattle share most (around 55%, on average) in the top (most variable) and bottom (most consistent) 10% of genes (Fig. 4b). This result was consistent after adjusting for the mean of expression (i.e., the coefficient of variation, CV, which is the ratio of the standard deviation to the mean) (Additional file 2: Fig. S11a, b). The variable genes were significantly engaged in tissue-relevant functions, while consistent genes were significantly involved in essential biological functions, such as system processes and stimulus detection (Additional file 6: Table S5).

Since inter-individual variation of gene expression is partially due to genetic factors, we then compared *cis*-eQTLs of genes across tissues between humans and cattle. We found that compared to all tested SNPs that were evenly distributed around transcription start sites (TSS), top *cis*-eQTLs of eGenes centered around TSS in both humans and cattle (Fig. 4c). However, there was a higher enrichment of *cis*-eQTLs around TSS in humans than in cattle (Additional file 2: Fig. S12), which might be due to the difference in LD patterns between the two species [24]. For instance, 95% of top *cis*-eQTLs were within 873 kb and 698 kb around TSS in cattle and humans, respectively (Additional file 2: Fig. S12). We found that the majority of eGenes (i.e., genes with *cis*-eQTLs) were tissue-specific (shared with less than five tissues) in humans and cattle (Fig. 4d). We observed a weak but significant correlation (Spearman’s *r* =0.15; *p* = 1.91×10^−13^) between the number of tissues, in which an eGene was detected on across two species (Fig. 4d). We further observed a significant overlap of eGenes within similar tissues between humans and cattle (Fig. 4e). For instance, eGenes in human blood had the highest enrichment with those in cattle blood, monocytes, and macrophage, and the same was observed for liver, muscle, and heart (Fig. 4e).

Furthermore, we observed that species-specific eGenes had a significantly (one-side Wilcoxon rank-sum test, *p* < 2.20×10^−16^) higher variability than other genes in the corresponding species (Fig. 4f). Additionally, we found that eGenes showed significantly higher differential expression between humans and cattle than non-eGenes (one-side Wilcoxon rank-sum test, *p* < 2.2×10^−16^), and conserved eGenes showed significantly higher differential expression than species-specific ones (Additional file 2: Fig. S11c). Overall, this suggests that *cis*-genetic variants may contribute to the inter-species differences in inter-individual variation of gene expression.

### Comparison of gene co-expression network

**Fig. 5:**
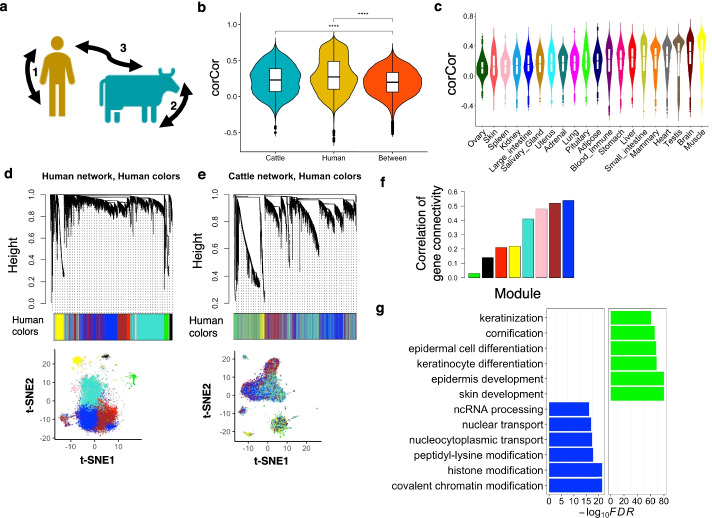
Comparison of gene co-expression network. **a** The diagram shows three comparisons, i.e., (1) between tissues within humans, (2) between tissues within cattle, and (3) within tissues between species. **b** Comparisons of corCor (measurement of gene co-expression conservation, details in “Methods”) among three groups. “****” represents the *P* < 0.0001 from one-side Student’s *t* test. **c** Comparisons of corCor in (3) across tissues. **d** The weighted gene co-expression network is constructed in human muscle using WGCNA package (“Methods”). Color represents gene co-expression module. Gene clustering is also visualized through *t*-SNE method. Each dot in the *t*-SNE plot represents a gene. **e** Similar with **d**, but the weighted gene co-expression network is constructed in cattle muscle. Genes in the cattle network are assigned same color as they in human modules to reflect the extent of module conservation between species. **f** Bar plot shows correlation of gene connectivity (measuring the conservation of gene co-expression module) between humans and cattle across human co-expression modules. **g** The top significantly (FDR<0.05) enriched Gene Ontology terms for genes in most conserved module (left) and most diverged module (right)

We estimated the conservation of gene co-expression profiles by calculating the correlation of the correlation coefficient (corCor, Methods) of genes between tissues within cattle, between tissues within humans, and within tissues between humans and cattle (Fig. 5a). We found that the overall corCors of genes among tissues within a species were significantly (one-side Student’s *t* test, *p* < 1.00×10^−4^) higher than those within tissues between species (Fig. 5b). This suggests that gene co-expression networks are less conserved than mean gene expression across species. However, we observed that tissues exhibited distinct conservation levels of gene co-expression between humans and cattle. For instance, muscle and brain showed the highest conservation levels, while ovary, skin, and spleen showed the lowest (Fig. 5c). In addition, we compared the conservation between gene expression and co-expression and found that expression-conserved genes showed significantly (Wilcoxon test, *p* < 2.20×10^−16^) higher co-expression conservation (i.e., corCors) than expression-diverged genes across tissues (Additional file 2: Fig. S13).

We here took muscle as an example, due to its highest conservation based on corCors, to show the conservation of individual gene co-expression modules between humans and cattle. We first conducted the weighted gene co-expression network analysis (WGCNA) in humans and cattle muscle samples to detect gene co-expression modules, separately (Methods). In general, we found that multiple gene co-expression modules were conserved between species (Fig. 5d–f). Genes in the most conserved module were significantly engaged in fundamental biological processes, such as histone modifications and covalent chromatin modifications. In contrast, genes in the least conserved gene module were significantly involved in skin development and keratinocyte differentiation (Fig. 5g). We repeated the analysis in all the 20 tissues and detected the most conserved and divergent gene co-expression modules, as well as found that these genes in different tissues were significantly enriched in distinct biological functions (Additional file 2: Fig. S14-15). For instance, genes of the most diverged module in blood were significantly enriched in neutrophil-mediated immunity, while genes of the most diverged module in brain were significantly enriched in mitochondrial ATP functions (Additional file 2: Fig. S15).

### Heritability of complex traits enriched in transcriptionally conserved genes

**Fig. 6:**
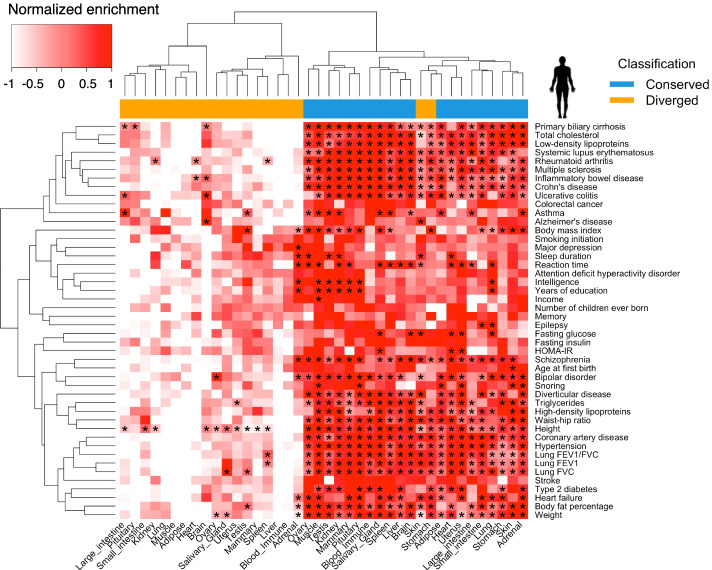
Heatmap of heritability enrichments of 46 human complex traits in transcriptionally conserved and diverged genes. Heritability enrichments obtained from LDSC for 46 human complex traits in transcriptionally diverged and conserved genes between humans and cattle (“Methods”). All orthologous genes are ranked (from largest to smallest) based on –log_10_*p* obtained from the differential gene expression analysis in each of 20 tissues between humans and cattle. The top and last 10% of genes are considered as transcriptionally diverged and conserved genes in each tissue, respectively. The enrichment is scaled to have mean of zero and variance of one by traits. “*” represents the adjusted *P*-value (FDR) < 0.05. Traits and tissues are clustered using the Hierarchical clustering method

**Fig. 7:**
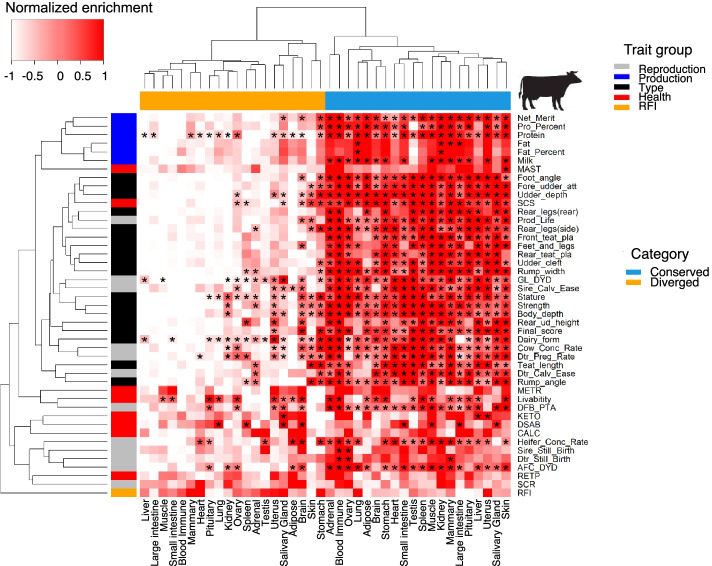
Heatmap of GWAS signal enrichments of 45 cattle complex traits in transcriptionally conserved and diverged genes. GWAS signal enrichments (i.e., −log_10_*p* from 10,000 times permutation, “Methods”) of cattle complex traits for transcriptionally diverged and conserved genes. All orthologous genes are ranked (from largest to smallest) based on –log_10_*p* obtained from the differential gene expression analysis in each of 20 tissues between humans and cattle. The top and last 10% of genes are considered as transcriptionally diverged and conserved genes in each tissue, respectively. The enrichment is scaled to have mean of zero and variance of one by traits. “*” represents FDR < 0.05

To better understand the genetic architecture underlying complex traits from an evolutionary point of view, we tested whether transcriptionally conserved genes were more enriched for genetic variants of complex traits than diverged genes (Methods). We analyzed GWAS summary statistics for 46 human complex traits with an average sample size of 327,973, and 45 cattle complex traits with a sample size of 27,214 (Additional file 7: Table S6). After ranking (from the largest to smallest) genes in each tissue according to their degree of differential expression (measured by −log_10_*p*) between humans and cattle, we considered the top and bottom 10% as diverged and conserved genes (*n* = 1731), respectively. The distributions of conserved and diverged genes across tissues are shown in Figure S16, and the majority of them were tissue-specific (shared with less than five tissues). In addition, the MAF and LD of SNPs were comparable between conserved and diverged genes (Additional file 2: Fig. S17). We found that genes with conserved mean expression explained more heritability or enriched more GWAS signals of complex traits than diverged ones (one-side Student’s *t* test, *p* < 2.20×10^−16^), and this was consistent across tissues and traits in both humans and cattle (Figs. 6 and 7, Additional files 8, 9 and 10: Table S7-9). We observed similar results for conserved and diverged genes that were detected from inter-individual variation and gene co-expression analyses (Additional file 2: Fig. S18). By further examining GWAS-discovered genes of 4756 complex traits (at least 10 genes per trait) using FUMA [25], we confirmed that conserved genes were significantly enriched for more complex traits GWAS signals than diverged ones, which was consistent across tissues except for skin, adrenal, and stomach (Additional file 2: Fig. S19). In addition to using the sum-based permutation method in cattle, we also employed the three-component GREML-LDMS model to estimate the per-SNP heritability of converged and diverged genes in three milk production traits (i.e., milk, fat and protein yield) (Additional file 10: Table S9), which had the largest sample size and the highest reliability of phenotypes [8, 26]. We found that the expression-conserved genes showed higher per-SNP heritability than DNA sequence-conserved genes and expression-diverged genes across most of the tissues (Additional file 2: Fig. S20a). We also found that the enrichment degrees based on the sum-based permutation test were significantly correlated with per-SNP heritability across tissues for milk and fat yield but not protein yield (Additional file 2: Fig. S20b). For other complex traits in cattle, the GREML-LDMS model could not converge properly across many tissues, mainly due to the variance components being estimated were close to zero. Compared to the GREML-LDMS or LDSC models, the sum-based permutation test only does the GWAS signal enrichment analysis rather than estimate proportions of genetic variance explained [27].

**Fig. 8:**
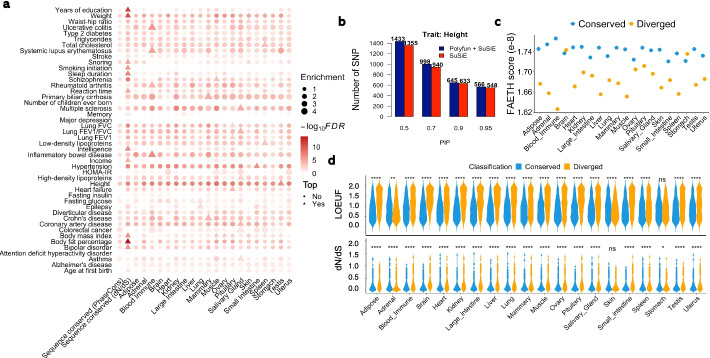
Transcriptionally conserved genes provide insights into the genetics of complex traits. **a** Heatmap of heritability enrichments obtained from LDSC for 46 human complex traits in transcriptionally and DNA sequence-conserved genes (measured by both dN/dS and PhastCons scores). All orthologous genes are ranked (from largest to smallest) based on –log_10_*p* obtained from the differential gene expression analysis in each of 20 tissues between humans and cattle. The bottom 10% of genes are considered as transcriptionally conserved genes in each tissue, respectively. The 10% of genes with the smallest dN/dS ratios are considered as DNA sequence conserved, whereas the 10% of genes with the largest PhastCons scores are also considered as DNA sequence conserved. In each trait, tissues or sequence-conserved genes with the top heritability enrichment are denoted as triangle, others as dots. **b** Bar plot shows the number of SNPs identified by PolyFun + SuSiE (blue) and SuSiE (red) at different PIP (posterior inclusion probability) cutoffs, respectively. **c** Comparison of FAETH scores of SNPs within transcriptionally conserved and diverged genes. SNPs located within 1000 bp up- and downstream of a gene are included. **d** Violin plot compares the LOEUF scores (up) and the dN/dS ratios (bottom) of transcriptionally conserved and diverged genes across 20 tissues. “*,” “**,” and “****” represents the *P* < 0.05, 0.01, 0.0001, respectively, from one-side Student’s *t* test

To test if the human-cattle conservation at the transcriptomic level could provide extra information than the conservation at the DNA level, we conducted the same heritability enrichment analysis for sequence-conserved genes (top 10% of genes with the highest sequence conservation between humans and cattle, measured by both Dn/Ds and PhastCons scores) together with expression-conserved genes. As shown in Fig. 8a, although sequence-conserved genes showed the highest enrichment for several traits (e.g., weight and years of education), expression-conserved genes in relevant tissues showed higher enrichments for certain traits. For instance, expression-conserved genes in blood showed the highest enrichment for immune/health traits (e.g., ulcerative colitis, systemic lupus erythematosus, rheumatoid arthritis, and inflammatory bowel disease). Similar findings were observed for genes showing conserved co-expression patterns (Additional file 2: Fig. S21a). For instance, we found that genes with conserved co-expression in brain showed the highest enrichment for schizophrenia, while genes in small intestine for immune-relevant traits (e.g., rheumatoid arthritis and inflammatory bowel disease), might be due to its immune function (Additional file 2: Fig. S21a). Although the interpretations of some trait-tissue associations were not straightforward due to the complexity in both complex traits and tissues, these results indicate that the transcriptome conservation in relevant tissues could provide additional information for interpreting complex trait genetics. We further compared the heritability enrichment of these 46 human traits for four groups of genes, i.e., the top 10% (most diverged), 40–50%, 50–60%, and bottom 10% (most conserved), ordered by −log_10_FDR (from largest to smallest) from the differential expression analysis between humans and cattle. We found that genes with higher conserved expression showed higher enrichments for the heritability of complex traits, and similar results were observed for gene co-expression (Additional file 2: Fig. S21b).

To investigate whether the transcriptional conservation between humans and cattle could help us identify new causal genes for complex traits, we took the well-studied human height as an example to perform the functionally informed (using conserved genes as functional priors) fine-mapping analysis using PolyFun + SuSiE [28, 29]. Comparing to results from the fine-mapping analysis without conserved genes (i.e., SuSiE [29] only), we fine-mapped more variants/genes for human height (Fig. 8b). Considering PIP (PolyFun + SuSiE) > 0.95 but PIP (SuSiE) < 0.95, we detected 53 variants for human height, out of which 10 were not genome-wide significant (*p* > 5×10^−8^) in the original GWAS. Out of these 10 variants, six could be mapped to protein-coding genes (Additional file 11: Table S10). By conducting phenome-wide association analysis for these genes using PheWAS (https://atlas.ctglab.nl/) [30], we found all these genes were associated with human height or relevant traits (Additional file 11: Table S10). We took *PFKP* and *CYP27B1* as examples in Figure S21c. To explore whether conserved genes could provide useful information in the cattle genomic prediction, we compared the FAETH scores of SNPs within conserved and diverged genes [26], which measures the predictive ability of SNPs for complex traits in dairy cattle. We found that SNPs in conserved genes had higher FAETH scores than those in diverged genes, consistent across all tissues except for stomach and brain (Fig. 8c).

We further explored the properties of transcriptionally conserved and diverged genes as a function of their tolerance to Loss-of-Function (LoF) variants (measured by Loss-of-Function observed/expected upper bound fraction, LOEUF) [31]. We observed that conserved genes had significantly smaller LOEUF scores (i.e., more depleted for LoF variation) compared to diverged genes across tissues, consistent for results from mean gene expression, inter-individual variation of gene expression, and co-expression networks (Fig. 8d, Additional file 2: Fig. S22a). Moreover, compared to diverged genes, we found that conserved genes had significantly smaller dN/dS ratios, indicating that transcriptionally conserved genes also exhibit more constrained protein-coding sequences (Fig. 8d, Additional file 2: Fig. S22b).

## Discussion

We comprehensively compared the transcriptomes of 20 tissues in humans and cattle. Despite the differences in experimental conditions and sample characteristics, we found that the mean expression of orthologous genes was, to a certain degree, conserved between humans and cattle. This is consistent with previous findings that the global gene expression pattern of orthologous genes between humans and mice is conserved, particularly for the central nervous system, liver, and heart/muscle [32]. We found that the brain had the highest correlation of median gene expression between humans and cattle, while testis and stomach had the lowest. This is in line with previous findings that suggested that the transcriptome evolves rapidly in testis but slowly in the central nervous system, based on a comparison of the gene expression profiles of six organs across ten mammals [33]. In addition, we investigated whether the gene expression of cattle-specific tissues (e.g., horn and rumen) were significantly correlated with those of human tissues, and found that cattle rumen showed the highest similarity with vagina, esophagus, and skin in humans compared to other tissues, which was due to the high enrichment of epithelial cells in these tissues. Meanwhile, cattle horns showed a low correlation of gene expression across all human tissues, while among them fallopian tube was the most similar one (Additional file 2: Fig. S23a-b).

Additionally, we found that inter-individual variability of gene expression was generally conserved in humans and cattle, which agrees with a previous comparison of gene expression between mice and humans [32]. However, we have taken this further and have shown that *cis*-genetic regulatory effects of gene expression (eGenes) were also conserved between humans and cattle, reflecting that the genetic regulation of gene expression evolves under similar evolutionary pressures among mammals [34]. In contrast, we found that gene co-expression networks were more conserved among tissues within a species than within corresponding tissues between species, suggesting that changes of gene co-expression networks play important roles in the adaptive evolution of species [2]. Of note, apart from the gene expression, many other functional elements (e.g., enhancers, ncRNAs, TFBS, and translation) and cell type composition might contribute to the difference in phenotypes between species.

The interpretation of the molecular mechanisms underlying complex traits has always been the research focus of genetics. GWASs provide strong evidence that most complex traits are extremely polygenic, yet the distribution of causal variants across the genome remains elusive. Finucane et al. reported that the heritability of complex traits was enriched in genomic regions with constrained DNA sequence across species [35]. We demonstrate that among orthologous genes, transcriptionally conserved genes had significantly higher enrichment for the heritability of complex traits than diverged genes in humans and cattle. We still noted that although on the relative scale, conserved genes seem to be more enriched with heritability than divergent genes, the total amount of heritability explained by conserved genes is not great in either humans or cattle on average across tissues. However, the top tissue for a complex trait could explain a relatively high proportion of heritability. For instance, 8% of SNPs in blood expression-conserved genes could explain 31% and 33% of heritability for inflammatory bowel disease and systemic lupus erythematosus, respectively (Additional file 9: Table S8). This finding suggested that expression-conserved genes contribute to the heritability of complex traits at a tissue-specific manner. Compared to previous studies [26, 35], we found a relatively lower enrichment of heritability in expression-conserved genes than sequence-conserved regions. This may be due to the previous studies considered the sequence-conserved regions in the entire genome, including both genic and intergenic regions, whereas we here only focused on orthologous genes between humans and cattle. Future research, with the increasing availability of functional annotation of animal genomes from the FAANG project [36], will allow examining the conservation of functionally regulatory elements (e.g., enhancer, promoter, and topologically associating domain) and non-coding RNAs in a wide range of tissues/cell types and species, as over 90% of GWAS hits are in non-coding regions [37].

## Conclusions

In summary, we showed the conservation of transcriptome among 20 common tissues between humans and cattle. We observed that transcriptionally conserved genes exhibited significantly higher enrichments for the heritability or GWAS signals of complex traits than diverged genes in both species. Our findings provided novel insights into the evolutionary basis of complex traits in humans and cattle.

## Methods

### RNA-seq samples in humans and cattle

All human RNA-seq samples were analyzed uniformly by human GTEx (v8) consortium previously [10], and the normalized gene expression (TPM) data were obtained in https://gtexportal.org/home/datasets. For cattle, we analyzed 11,642 publicly available RNA-seq runs from 8536 samples (by July 2019) using a similar pipeline as human GTEx [10, 11]. Briefly, we filtered out low-quality reads using Trimmomatic (v0.39) and mapped clean reads to cattle ARS-UCD1.2 reference genome using STAR (v2.7.0). We obtained TPM of all annotated genes (*n* = 27,608) in Ensembl (v96) using Stringtie (v2.1.1). We kept cattle samples with unique mapping reads > 70% and the number of clean reads > 800,000 for subsequent analysis. All gene expression data and the metadata of samples in cattle were available in https://cgtex.roslin.ed.ac.uk/. Ultimately, we obtained normalized gene expression values (TPM) for 10,830 and 4866 RNA-seq samples from 20 common tissues in humans and cattle, respectively. We obtained 17,315 one-to-one orthologous genes and their annotation information from Ensembl (v96).

### Sample clustering and differential gene expression analysis

We used the function *IntegrateData(anchorset = expression, dims = 1:30)* in R Seurat package [38] to combine expression values of orthologous genes in humans and cattle by removing hidden confounding factors. Afterward, we performed *t*-distributed stochastic neighbor embedding (t-SNE), implemented in Rtsne [39]: *Rtsne(expression,dims = 2, perplexity=150, theta=0.5, verbose=TRUE, max_iter = 1000, check_duplicates = FALSE,partial_pca = T, num_threads=50)* to project samples to a two-dimensional space based on corrected expression values of orthologous genes. We calculated the median gene expression in each tissue in cattle and humans separately, to represent the “true” expression of the particular tissue in each species. We then performed hierarchical clustering using R package *pheatmap* [40]: *pheatmap(corr_mat, cluster_rows = T,cluster_cols = T, clustering_distance_rows ="correlation", clustering_distance_cols = "correlation")*, to explore the relationship of tissues in humans and cattle based on the median gene expression.

We detected genes with tissue-specific expression using R *Limma* package [41] with function *model.matrix*, *lmFit*, *contrasts.fit*, *eBayes*, and *topTable* by comparing gene expression of samples in a given tissue to those in the remaining tissues. We also employed *Limma* package to detect species-specific genes in each tissue between humans and cattle. *Limma* returned adjusted *P*-values for multiple testing using Benjamini and Hochberg methods (FDR). Here, we used log2(FC) > 1.5 and FDR < 0.05 to detect tissue-specific genes. In contrast, we used FC > 1.2 and FDR < 0.05 to identify genes differentially expressed between species, as the differences in gene expression are much bigger between tissues within species than within tissues between species. We also ranked genes according to their degrees of differential expression (–log_10_p) from DEG analysis between humans and cattle. We then considered the top and last 10% of all orthologues genes as the most diverged and conserved genes for partitioning the heritability of complex traits.

We obtained and analyzed 113 RNA-seq samples from 14 tissues in mice from recount3 (http://rna.recount.bio/) [17, 18]. We used *Limma* package [41] to identify species-specific genes for human vs. cattle, and human vs. mouse, similarly as described above.

### Detection and comparison of chromatin states between humans and cattle

We analyzed genome-wide sequence data of five epigenetic marks (i.e., ATAC-seq and ChIP-Seq for H3K27ac, H3K27m3, H3K4m1, and H3K4m3) and their corresponding background inputs in six common tissues (two biological replicates per tissue) in humans and cattle. The tissues included liver, lung, spleen, muscle, brain, and adipose. We downloaded the human data from ENCODE (https://www.encodeproject.org/), and cattle data from FAANG (https://www.faang.org/). Using BWA algorithm with default settings [42], we mapped human and cattle data to GRCh38 and ARS-UCD1.2 reference genomes, respectively. We then employed a multivariate Hidden Markov Model (HMM), implemented in ChromHMM v1.18 [43], to define 15 chromatin states using 200-bp sliding windows through combining these epigenomic marks across samples in humans and cattle, separately. We calculated the enrichment fold of each chromatin state in TSS ±2kb of diverged genes as (C/A)/(B/D), where A is the number of bases in the state, B is the number of bases in TSS ±2kb, C is the number of bases overlapped between the state and TSS ±2kb, and D is the number of bases in the entire genome.

### Detection of differentially variable genes between species

We used the following F-test to conduct differential variability analysis of gene expression in each of 20 tissues between humans and cattle [44]. In a given tissue, $$f=\frac{{s_1}^2}{{s_2}^2}$$, where *s*_1_^2^ and *s*_2_^2^ are variances of gene expression values (i.e., log_2_TPM) in humans and cattle, respectively, with the null hypothesis: *s*_1_^2^ = *s*_2_^2^. Under the assumption that the expression of a gene follows a normal distribution, *f* follows an *F*_(*n* − 1, *m* − 1)_ distribution (where *n* and *m* is the number of human samples and cattle samples, respectively), from which we obtained *P*-values. We adjusted *P*-values for multiple testing using Benjamini and Hochberg methods (FDR) with R function *p.adjust(variance_diff$p_value,method = "BH").* According to their –log_10_FDR, we then ranked genes (from largest to smallest) and considered the top and last 10% genes as diverged and conserved genes.

Furthermore, we obtained fine-mapped results of *cis*-eQTLs for similar tissues in humans and cattle from the Human GTEx project [10] (https://gtexportal.org/home/datasets) and Cattle GTEx project (http://cgtex.roslin.ed.ac.uk/), respectively. We considered genes with significant *cis*-eQTLs (*P* < 10^−5^) as eGene. We used the hypergeometric test, implemented in *phyper* function in R: *phyper(Overlap-1 , human, 17315-human , cattle, lower.tail= FALSE)*, to test the significance of overlaps of eGenes across tissues between species. We adjusted *P*-values for multiple testing using the Benjamini-Hochberg method (FDR).

### Gene co-expression analysis

We employed an R package, *MergeMaid* with function *intCor(merged,method="pearson",exact=F)* [45], to calculate corCors for all orthologous genes in three scenarios, (1) between tissues within cattle, (2) between tissues within humans, (3) within tissues between humans and cattle. For a gene A in an expression matrix of a tissue in a species containing *n* genes, we computed the Spearman’s correlation of expression value between gene A and any other genes, resulting in a vector of length *n*-1 (vector A). Given gene A’ is the ortholog of gene A on the other expression matrix (a different tissue or species), we obtained a vector of length *n*-1 (vector A’) similarly by calculating Spearman’s correlation of A’ with any other genes in the same order as in vector A. We then computed the correlation between vector A and vector A’ (corCor), to represent the conservation level of gene A in terms of the co-expression network between two groups. We also applied another R package WGCNA with function *cutreeDynamic(dendro = hierTOM, distM = distTOM, deepSplit = 2, pamRespectsDendro = FALSE, minClusterSize = minModuleSize)* [46], to detect the weighted gene co-expression networks within each tissue in humans and cattle separately. We assigned colors to genes in each co-expression module using function *labels2colors(dynamicMods)*.

### Stratified LD score regression (S-LDSC) and POLYgenic FUNctionally informed fine-mapping (PolyFun) analysis for human complex traits

To determine whether transcriptionally conserved genes explain the more genetic variance of complex traits than diverged genes, we employed the commonly used stratified LD score regression to partition the heritability of human complex traits into distinct functional categories [35]. The stratified LD scores were calculated in 500 kb window using 1000G Phase 3 European human samples. Only HapMap3 SNPs with INFO≥0.9 and MAF > 0.05 in 1000G European samples were included for LD score calculation. We obtained 1000G samples and default SNP weights from (https://github.com/bulik/ldsc).

We collected GWAS summary statistics for 46 human complex traits from a public database (Additional file 6: Table S5). These GWAS are mainly European-ancestry based, with an average sample size of 327,973, a good overlap with HapMap3 panel, a mean *χ*^2^ statistics of > 1.02 and a heritability *Z*-score of > 4 [47]. For each GWAS summary, default quality control was performed by LDSC to remove GWAS SNPs that are with MAF ≤ 0.01, INFO ≤ 0.9, genotype call rate ≤ 0.75, duplicated rsid, out-of-bounds *P*-value, extreme large *χ*^2^ statistics, strand ambiguous variants, and in discordance with those used in previous LD score calculation^32^. After filtering, the average number of markers for LDSC regression was over one million. A summary of GWAS used in this study and the LDSC regression results of base model (without partitioning heritability) are available in Tables S6 and S7, respectively.

We tested 41 functional categories for each trait, including 20 groups of the most conserved genes (a group per tissue), 20 groups of the most diverged genes and a group of all SNPs to capture the total heritability. We extended −/+50 kb of gene regions to include their *cis*-regulatory regions. We detected the most conserved/diverged genes within each of 20 tissues between humans and cattle in three scenarios below:

1. The top 10% (diverged) and last 10% (conserved) of all orthologous genes based on –log_10_*P* (ranked from largest to smallest) from differentially expression analysis between humans and cattle;
2. The top 10% (diverged) and last 10% (conserved) of all orthologous genes based on –log_10_*P* (ranked from largest to smallest) from differential variability analysis between humans and cattle.
3. The top 10% (conserved) and last 10% (diverged) of all orthologous genes based on corCor scores (ranked from largest to smallest).

PolyFun [28] is an extension of S-LDSC [35] that computes SNP prior causal probabilities via the same statistical framework (Step 1). These prior causal probabilities were then used priors in SuSiE [29] for the fine-mapping (Step 2) analysis. Settings in Step 1 were the same as S-LDSC [35] analysis with two exceptions. First, we only annotated 21 functional categories, including a group of all SNPs to capture the total heritability and 20 groups of the conserved genes between humans and cattle. Second, to gain more power, we used the UK Biobank data as the reference panel and the LD scores were computed using pre-computed UK Biobank LD matrices composed of ~19M SNPs from [28]. In Step 2, we performed fine-mapping analysis using two models in SuSiE [29]. The first model only took into account LD information (i.e., pre-computed UK Biobank LD matrices), whereas the second model considered both LD information and SNP prior causal probabilities estimated from Step 1. We compared how many loci were detected at difference posterior causal probability (PIP) thresholds between these two models.

### GWAS signal enrichment analysis for cattle complex traits

We collected GWAS summary statistics from 45 agronomic traits of economic importance in cattle, including reproduction (*n* = 12), production (milk-relevant; *n* = 6), body conformation (*n* = 18), health (immune/metabolic-relevant; *n* = 8) and one feed efficiency trait (i.e., residual feed intake, RFI). For body type, reproduction and production traits, we conducted a single-marker GWAS by fitting a linear mixed model in 27,214 U.S. Holstein bulls as described previously [8]. For health traits, we conducted GWAS using the same method in a subset (ranging from 11,880 for hypocalcemia to 24,699 for livability) of the 27,214 available bulls [48]. GWAS of feed efficiency (i.e., residual feed intake, RFI) was conducted based on 3947 Holstein cows [49].

As linkage disequilibrium (LD) pattern is extremely complicated in the cattle population, we applied a commonly used genotype cyclical permutation method, implemented in QGG package [50], to test the enrichment of cattle GWAS signals in each of the functional categories defined above. Previous studies showed that results from this method were highly correlated with those from LDSC and other GWAS signal enrichment methods [5, 51, 52].

$${\it T}_{sum}={\textstyle\sum_{i=1}^{m_f}}b^2,$$

where *m*_*f*_ is the total number of genomic markers linked to a list of genes (e.g., transcriptionally conserved genes in liver), and *b* is the marker effect from single-marker GWAS. The markers linked to different genes were often not in LD. We controlled marker-set sizes and LD patterns among makers through applying a genotype cyclical permutation strategy [53]. To obtain an empirical *P*-value for a gene list, we repeated this permutation procedure 10,000 times and employed a one-tailed test of the proportion of random summary statistics greater than that observed.

In order to explore the patterns of MAF and LD between conserved and diverged groups, we calculated the MAF and LD using PLINK (v.1.9) (--freq and --r2) of 20 gene groups’ SNPs.

### GREML-LDMS

For cattle, we applied the 3-component GREML-LDMS model below [54] to estimate how much genetic variance in three milk production traits (i.e., milk, fat, and protein yield) could be attributed to common genetic variants within distinct gene groups (e.g., expression-conserved and divergent genes). This analysis included 27,235 individuals and 3,085,572 autosomal variants with MAF > 5% [8].

$$\boldsymbol{y}={\mu} +{\boldsymbol{g}}_{con}+{\boldsymbol{g}}_{div}+{\boldsymbol{g}}_{rest}+\boldsymbol{e};$$

where 𝒚 was the vector of phenotypes of individuals being analyzed. The phenotypes were deregressed transmitting ability, i.e., the additive genetic values of cattle after correcting for all the known fixed effects. ***μ*** is global mean, ***g***_*con*_ was the vector of polygenic effects for SNPs within conserved genes, where ***g***_*con*_~N(0, 𝐆_*con*_𝜎^2^ 𝑔), 𝐆_*con*_ was the genomic relationship matrix (GRM) calculated by SNPs within conserved genes; ***g***_*div*_ was the vector of polygenic effects for SNPs within diverged variants, where ***g***_*div*_~N(0, 𝐆_*div*_𝜎^2 ^𝑔), 𝐆_*div*_ was the GRM calculated by SNPs within diverged genes; ***g***_*rest*_ was the vector of polygenic effects for the rest of SNPs, where ***g***_*rest*_~N(0, 𝐆_*rest*_𝜎^2^ 𝑔), 𝐆_*rest*_ was the GRM calculated by the rest variants; and ***e*** was the vector of residual. We applied GREML in GCTA [55] to calculate the heritability of each trait, ℎ^2^_*con*_ and ℎ^2^_*div*_, respectively. For each group, the per-variant ℎ^2^ was calculated as the ℎ^2^ divided by the number of SNPs in the corresponding group.

### Other downstream bioinformatics analysis

We used the hypergeometric test, implemented in *clusterProfiler* R package [56], to explore the function of a list of genes based on Gene Ontology (GO) database. We applied function *bitr(gene_list, fromType="ENSEMBL", toType = c("SYMBOL", "ENTREZID"), OrgDb=org.Hs.eg.db, drop = T)* to translate Ensembl ID to gene symbols, and *enrichGO(gene = gene_cattle$ENTREZID, OrgDb= org.Hs.eg.db, ont = "BP", pAdjustMethod = "BH", minGSSize = 1, pvalueCutoff = 0.05, qvalueCutoff = 0.05, readable = TRUE)* to detect the enriched GO terms. We considered GO terms with FDR < 0.05 as significant.

We utilized tspex [57] to calculate the tau score (*τ*) (ranging from 0 to 1, with 1 for highly tissue-specific genes and 0 for ubiquitously transcribed genes) for each orthologous gene to measure its tissue-specific expression in humans and cattle. In each tissue, we used the median gene expression across all samples to calculate *τ* scores.

To explore whether transcriptionally conserved/diverged genes were significantly enriched for GWAS signals of complex traits in humans, we performed gene-set enrichment analysis for our conserved/diverged genes on reported gene-sets for a large number of human complex traits and diseases from GWAS-catalog using GENE2FUNC in FUMA (https://fuma.ctglab.nl/) [25]. To investigate the association of a gene/variant with a variety of complex traits, we performed phenome-wide association analysis using PheWAS (https://atlas.ctglab.nl) (https://atlas.ctglab.nl) [30], which includes totally 4756 GWAS. Only GWAS traits with Bonferroni-corrected *P*-value < 0.05 were displayed in the PheWAS plots.

## Supplementary Information

Additional file 1: Table S1. Summary of RNA-seq samples in humans and cattle.

Additional file 2: Supplementary Figs. S1-23. Comparative transcriptome in large-scale human and cattle populations.

Additional file 3: Table S2. Significantly enriched Gene Ontology terms for three groups of tissue-specific genes.

Additional file 4: Table S3. Significantly enriched Gene Ontology terms for up-regulated genes in cattle and humans.

Additional file 5: Table S4. Significantly enriched Gene Ontology terms for genes with more conserved expression between human and cattle than between human and mouse.

Additional file 6: Table S5. Significantly enriched Gene Ontology terms for genes with variable and consistent expression across tissues in humans and cattle.

Additional file 7: Table S6. Summary of 46 GWAS in humans.

Additional file 8: Table S7. Summary of LDSC results of base model (without partitioning heritability) for 46 human complex traits.

Additional file 9: Table S8. Heritability enrichment analysis of expression-conserved and divergent genes in human complex traits using LDSC.

Additional file 10: Table S9. Partitioning heritability with expression-conserved and divergent genes in milk production traits using GREML-LDMS.

Additional file 11: Table S10. Summary of novel variants detected by PolyFun + SuSiE in human height.

Additional file 12. Peer review history.

## Data Availability

All gene expression data analyzed in this study are publicly available in https://gtexportal.org/home/datasets [58] for humans and https://cgtex.roslin.ed.ac.uk/ [59] for cattle. All scripts codes used in this study can be found in https://github.com/B160389-2019/Comparative-Project [60].

## Abbreviations

*cis*-eQTLs: *cis*-expression Quantitative Trait Loci
corCor: Correlation of the Correlation coefficient
DEG: Differentially expressed gene
FAANG: Functional Annotation of Animal Genomes
GO: Gene Ontology
GTEx: Genotype-Tissue Expression
GWAS: Genome-Wide Association Studies
LD: Linkage disequilibrium
LDSC: Linkage Disequilibrium Score Regression
LOEUF: Loss-of-function Observed/Expected Upper bound Fraction
MAD: Median absolute deviation
