# Supplementary material for: Comparative transcriptome in large-scale human and cattle populations
Source: Genome Biol. 2022 Aug 22;23:176. doi: 10.1186/s13059-022-02745-4 (PMC9394047; doi:10.1186/s13059-022-02745-4)
Supplement: Supplementary file 2 — Additional file 2: Supplementary Figs. S1-23. Comparative transcriptome in large-scale human and cattle populations. [file 13059_2022_2745_MOESM2_ESM.pdf]

Additional file 2: Supplementary Figures 1-23

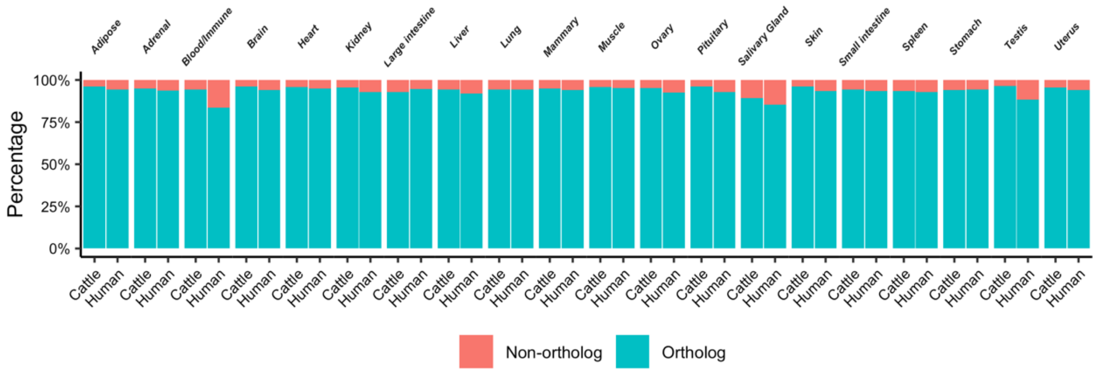

Fig. S1. The percentage of transcriptional outputs of orthologous and non-orthologous genes. Genes are classified into orthologues (blue) and non-orthologues (red) in humans and cattle. Orthologous genes generally contributed to a large majority of transcriptional outputs (sequence reads) across 20 tissues being studies.

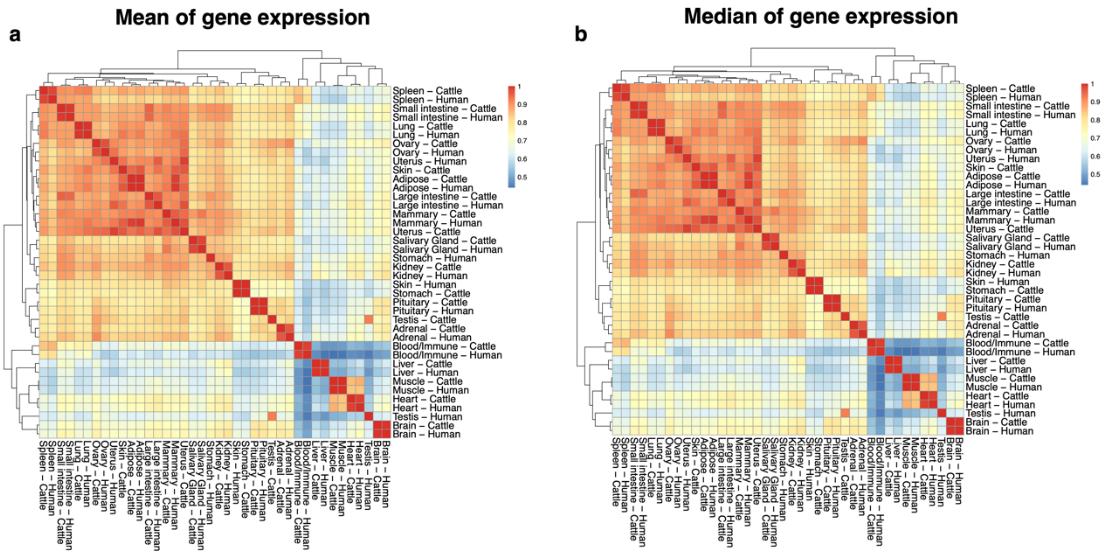

Fig. S2. Hierarchical clustering of tissues in humans and cattle. a, Heatmap of hierarchical clustering of tissues based on Pearson's correlation of mean expression of Orthologous genes. **b**, the same as **a**, but based on median expression value.

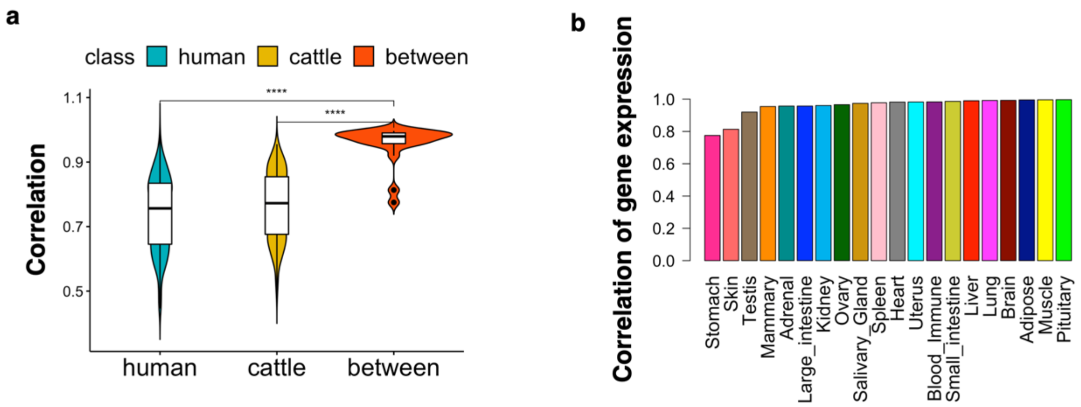

Fig. S3. Comparison of gene expression between human and cattle tissues. a, Violin plot compares three groups of correlations, 1) between tissues within humans (human), 2) between tissues within cattle (cattle), and 3) within tissues between species (between). "****" represents the adjusted *P*-value (FDR, one-side Student's *t*-test) less than 0.0001. Gene expression profiles are significantly more conserved within the same tissues between humans and cattle than between tissues within the same species. **b**, Bar-plot shows the correlations of gene expression in each of 20 tissue between humans and cattle. Brain, pituitary, muscle and adipose have the highest similarity between species, while stomach and skin have the lowest.

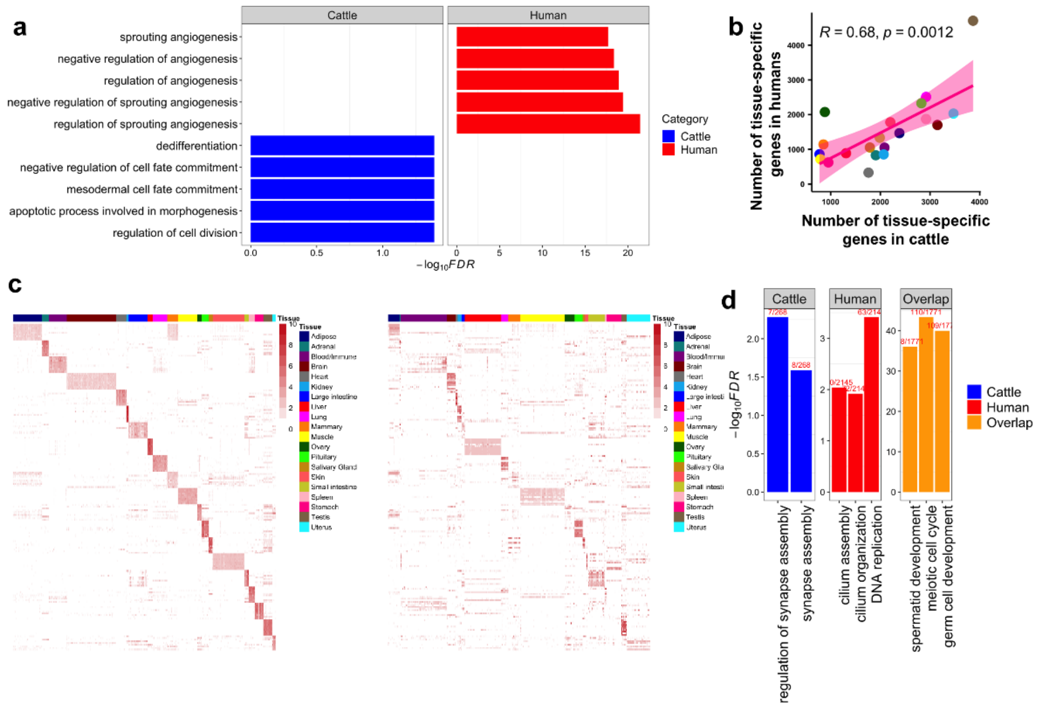

Fig. S4. Characterization of tissue-specific genes. a, Significantly enriched Gene Ontology (GO) terms for non-expressed genes (TPM < 0.1) in cattle and humans. **b**, Spearman's correlation of the number of tissue-specific genes between human and cattle tissues. **c**, Heatmap for expression (log2 (TPM+0.25)) of top 10 human tissue-specific genes across human samples (left). Each row represents a tissue-specific

gene and each column represents a sample from each of 20 human tissues. Heatmap for expression of the same tissue-specific genes above across cattle samples (right). **d,** Significantly enriched GO terms for species-specific (Cattle or Humans) genes and species-conserved (Overlap) genes in testis.

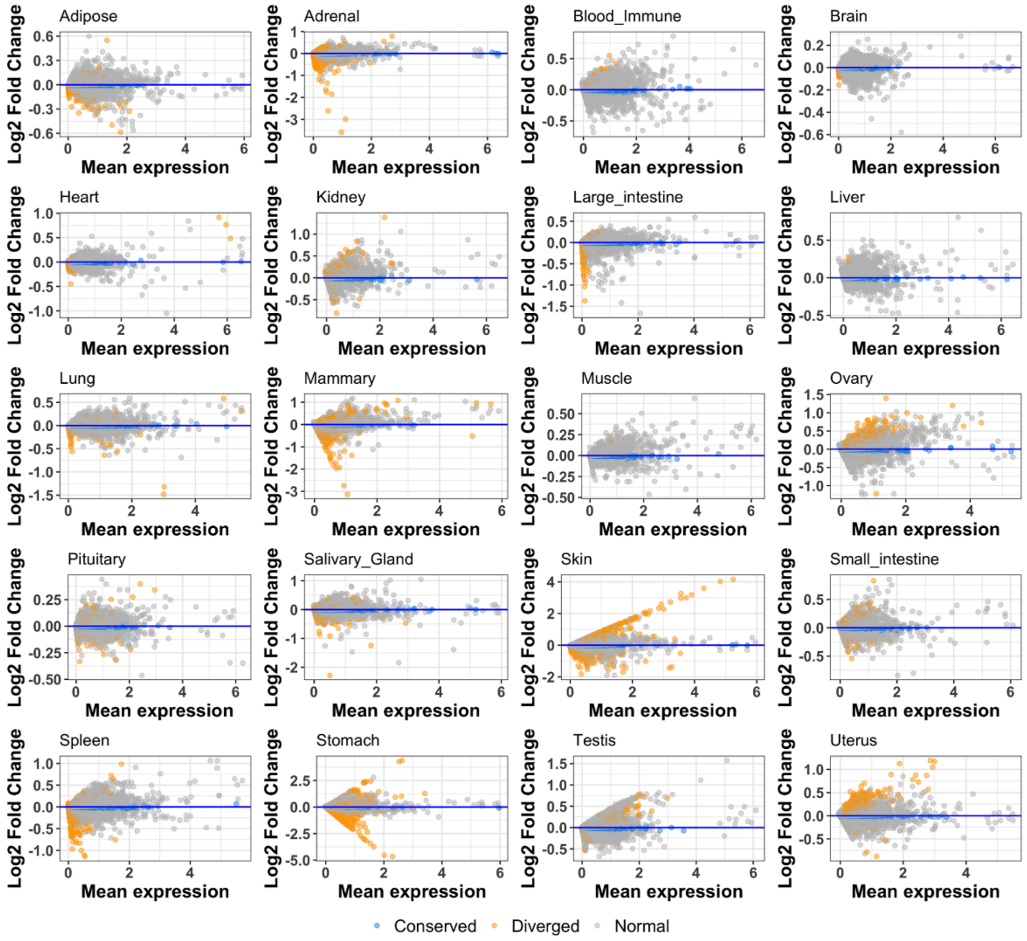

Fig. S5. MA-plots for differential expression analysis in each of 20 tissues between humans and cattle. X-axis represents the log2(fold change) of expression between human and cattle tissues. Y-axis represents the average gene expression across samples. All orthologous genes are ranked (from largest to smallest) based on $-\log_{10}p$ obtained from the differential gene expression analysis in each of 20 tissues between humans and cattle. The top and last 10% of genes are considered as transcriptionally diverged (orange) and conserved (blue) genes in each tissue, respectively.

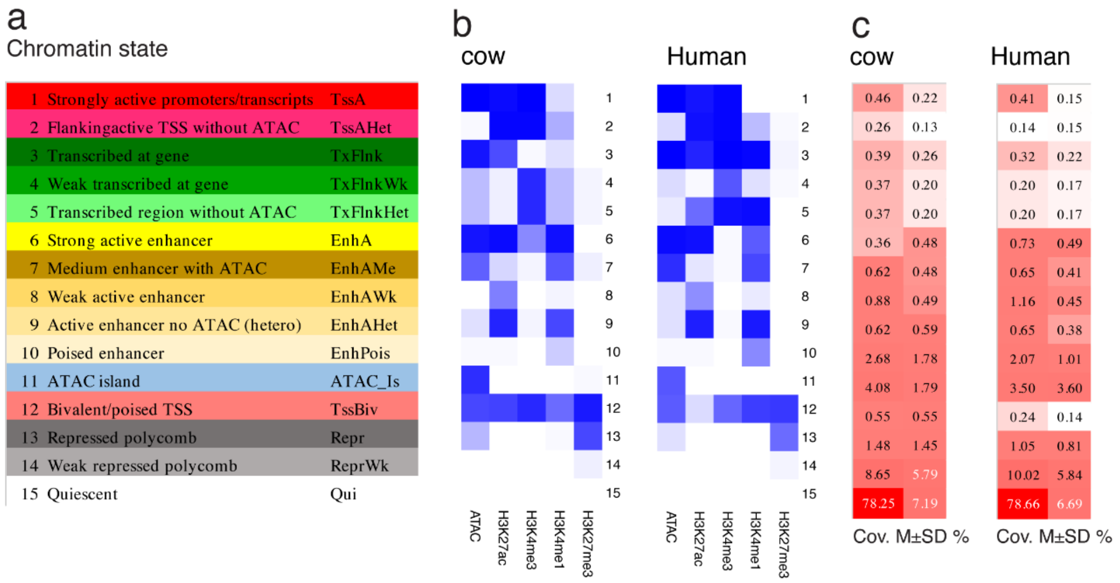

Fig. S6. Detection of 15 chromatin states using epigenetic marks in humans and cattle. a, 15 chromatin states predicted using ChromHMM. These states are defined using the epigenetic data (ATAC-seq, and ChiP-seq for H3K4me3, H3K4me1, H3k27ac and H3K27me3) from six tissues (i.e., liver, lung, spleen, muscle, brain and adipose) in humans and cattle, separately. **b**, Emission probabilities of individual epigenetic marks in each state in cattle and humans. **c**, Mean genomic coverages of chromatin states and standard deviation (SD) among six tissues.

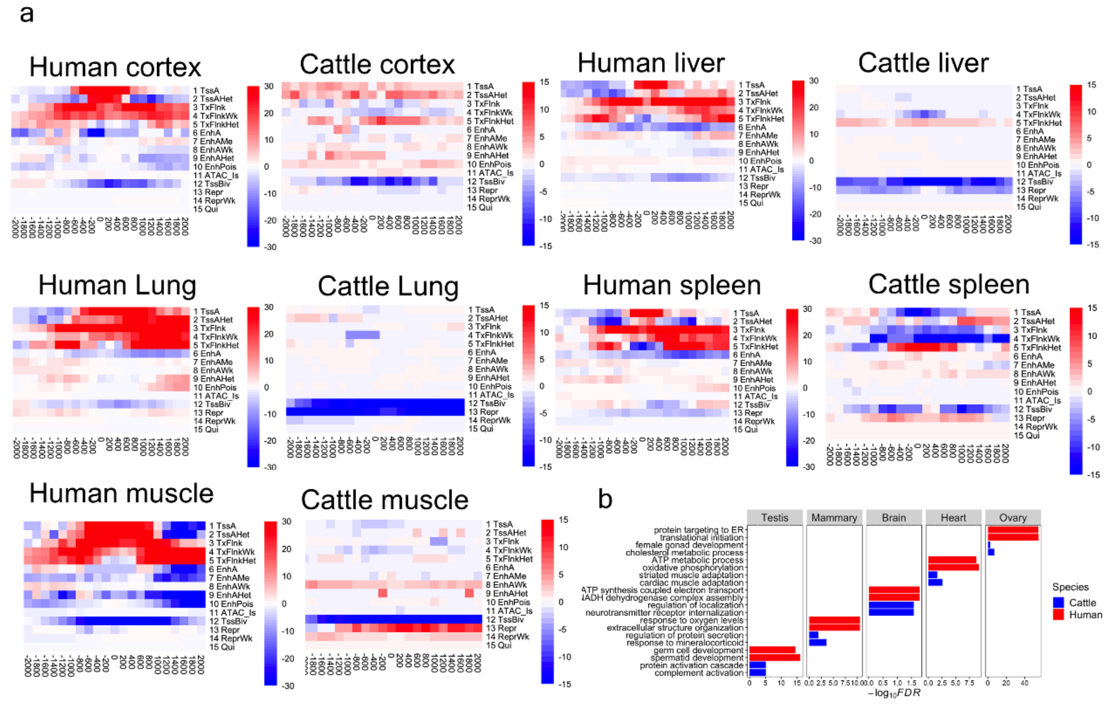

Fig. S7. Alteration of chromatin states and functional annotation for differentially expressed genes (DEGs) between humans and cattle. a, The changes of enrichment folds

of chromatin states round (±2kb) transcriptional start site (TSS) of top 500 up-regulated genes in humans and cattle across five tissues. **b**, Significantly (FDR < 0.05) enriched Gene Ontology terms for up-regulated genes in humans and cattle.

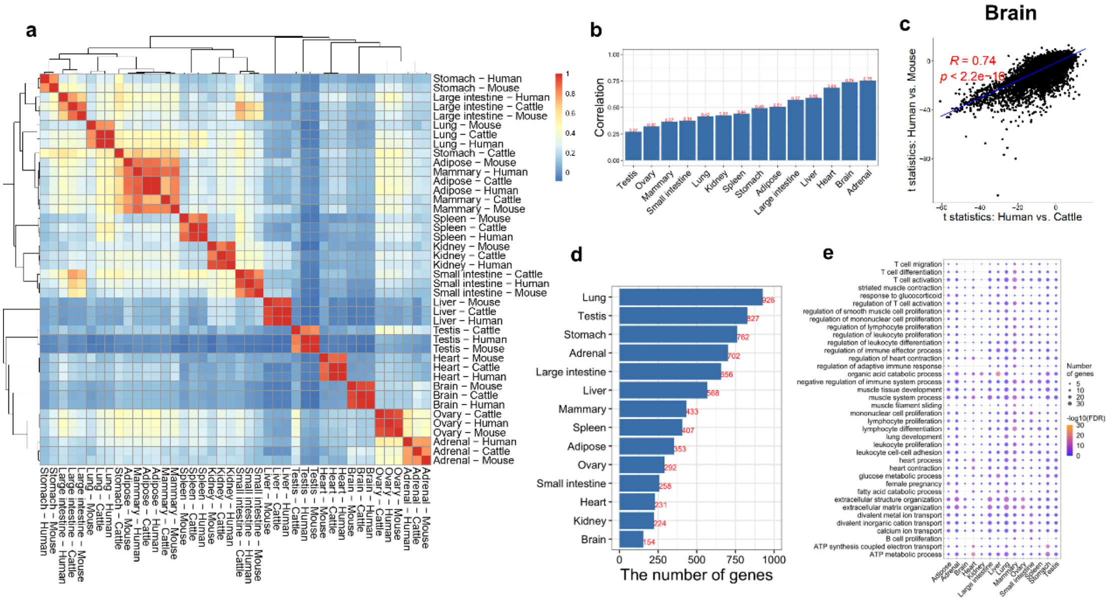

Fig. S8. Comparative transcriptome of 14 tissues among humans, cattle and mice. a, Heatmap of hierarchical clustering based on Pearson's correlation of median expression of orthologous genes. **b**, Barplot showing the correlation between the *t*-statistics (measuring the difference in gene expression) of humans *vs*. mice and humans *vs*. cattle. **c**, The scatter plot for t-statistics of humans vs. mice and those of humans vs. cattle in brain. **d,** Bar plot for number of genes with conserved expression (|Fold Change| < 1.2 and FDR > 0.05) between humans and cattle but diverged expression between humans and mice (|Fold Change| > 1.2 and FDR < 0.05). **e**, Enriched GO terms (Biological Process) for genes that are transcriptionally conserved in humans vs. cattle but diverged in humans vs. mice.

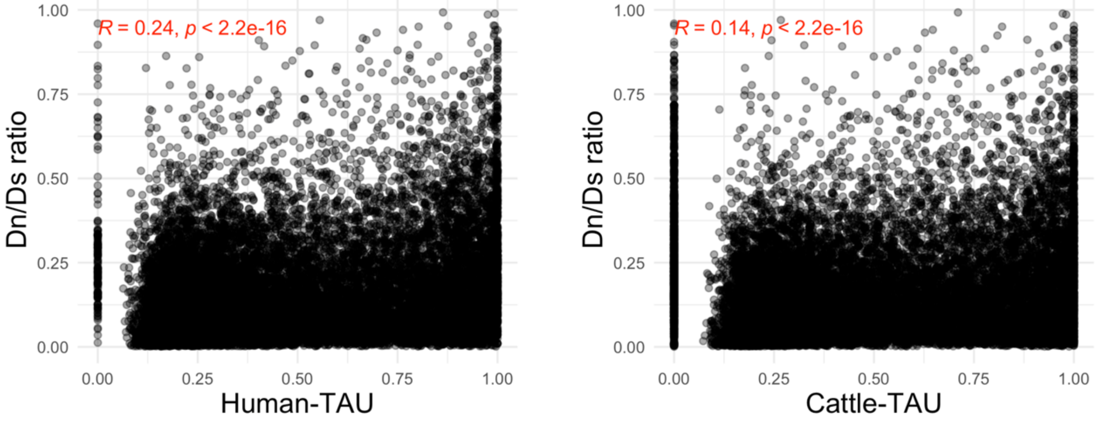

Figure S9. The Spearman correlation between dN/dS ratio and TAU of orthologous genes in humans (right) and cattle (left).

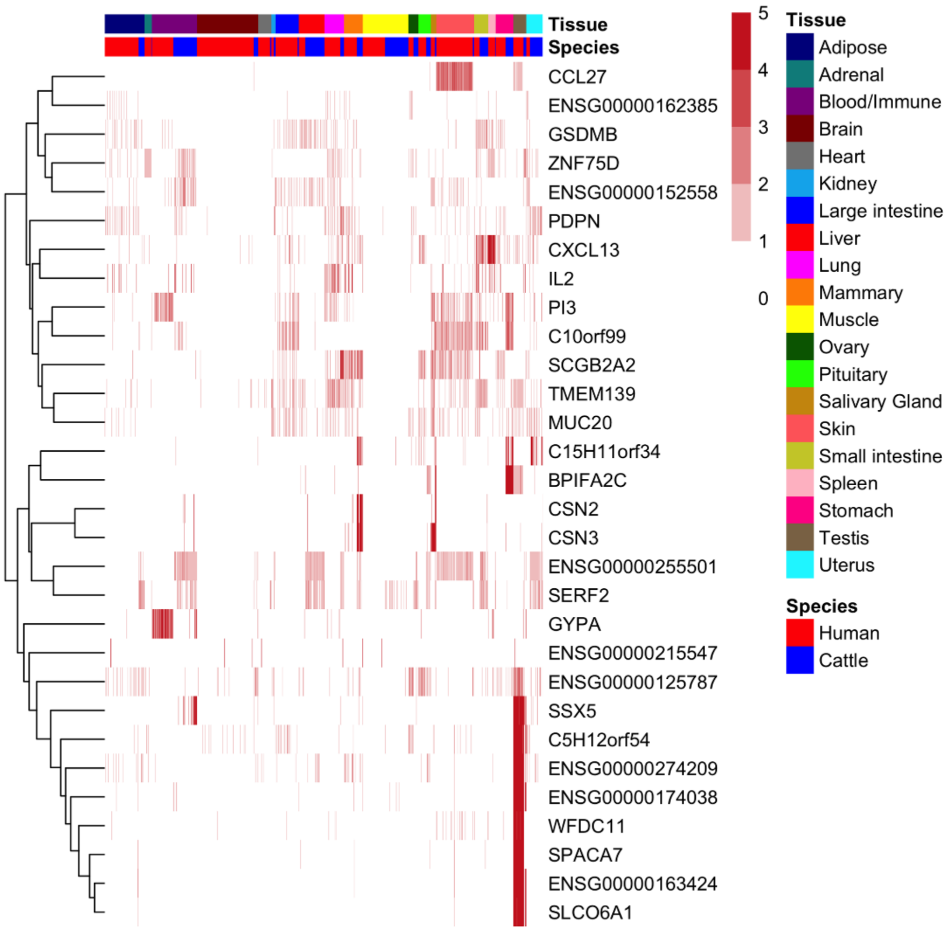

Fig. S10. Expression profiles of 30 positively selected genes (dN/dS > 1) between humans and cattle. Colour represents $\log_2$-transformed expression value, i.e., $\log_2$ (TMP+0.25). Each row represents a gene, and each column represents a sample from a certain tissue in humans or cattle.

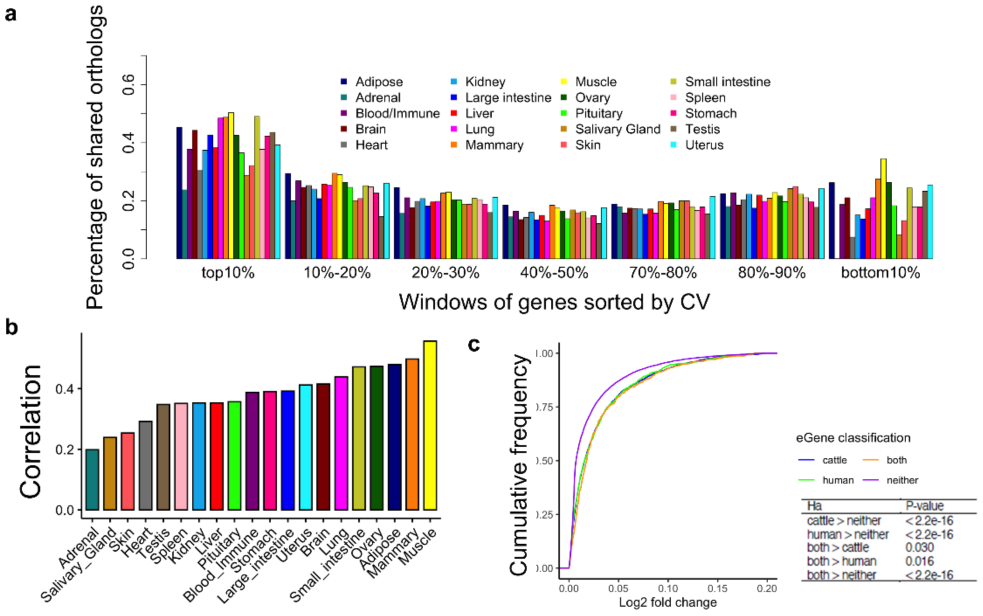

Fig. S11. Coefficient of variation in gene expression. **a**, Percentage of shared in each 10% of orthologous genes between humans and cattle. Genes are ranked (from largest to smallest) by the coefficient of variation (CV) in a tissue within a species. **b,** Spearman's correlation of CV in each tissue between humans and cattle. **c**, The empirical cumulative distribution function of species-specific $\log_2$(fold-change) for four groups of genes, i.e., cattle-specific eGenes (cattle), human-specific eGenes (human), species-shared eGenes (both), non-eGenes in neither species (neither). eGenes are more differentially expressed between human and cattle than non-eGenes, and shared eGenes (detected both in human and cattle) are even more differentially expressed. P values are obtained from one-side Student's *t*-test.

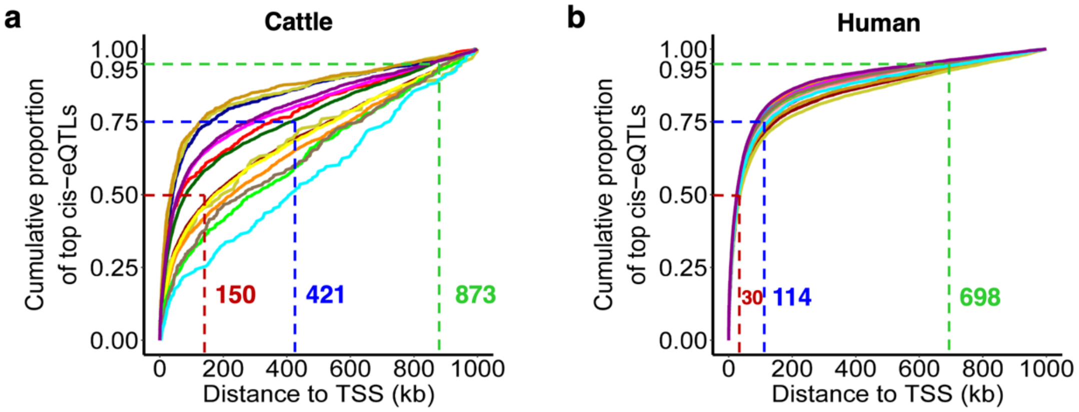

Fig. S12. The distance of top cis-eQTLs to the TSS in cattle and human tissues. a, Cumulative distribution of top cis-eQTLs to TSS across cattle tissues. **b**, Cumulative distribution of top cis-eQTLs to the TSS across human tissues (obtained from https://gtexportal.org/home/datasets)

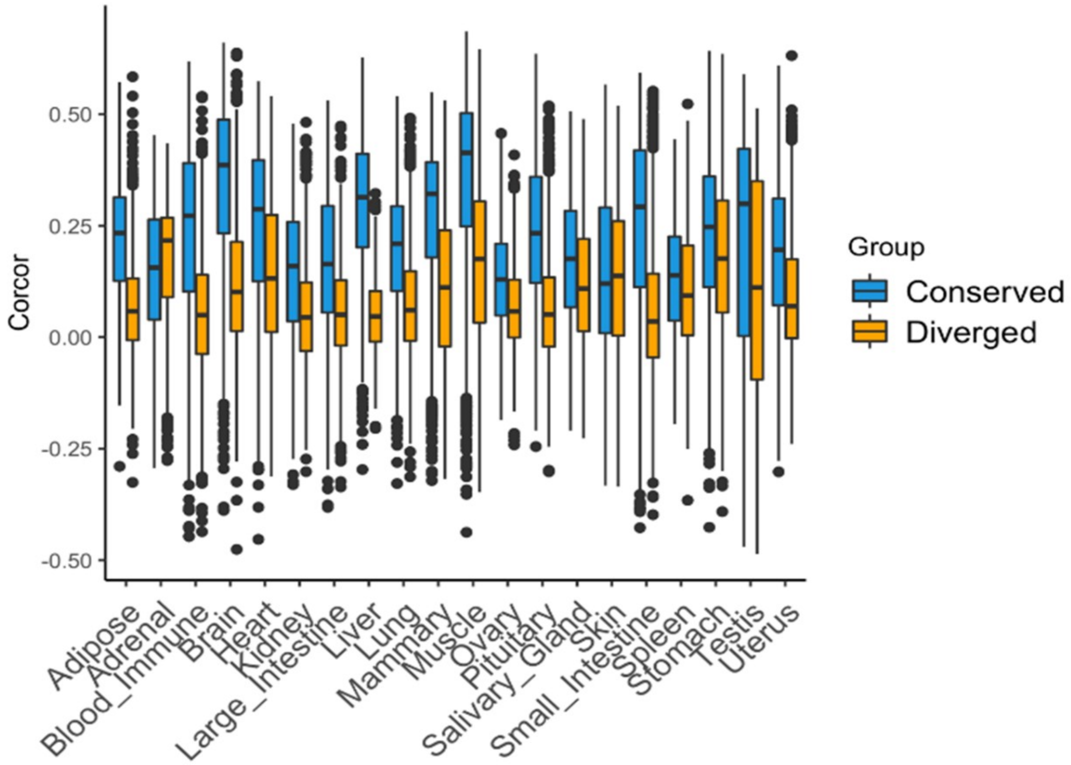

Fig. S13. Comparison of CorCor scores between expression-conserved and -diverged genes. Conserved and diverged genes represent the last (conserved) and top (diverged) 10% of all orthologous genes ordered by -$\log_{10}$FDR from the differential expression analysis in each of 20 tissues between humans and cattle.

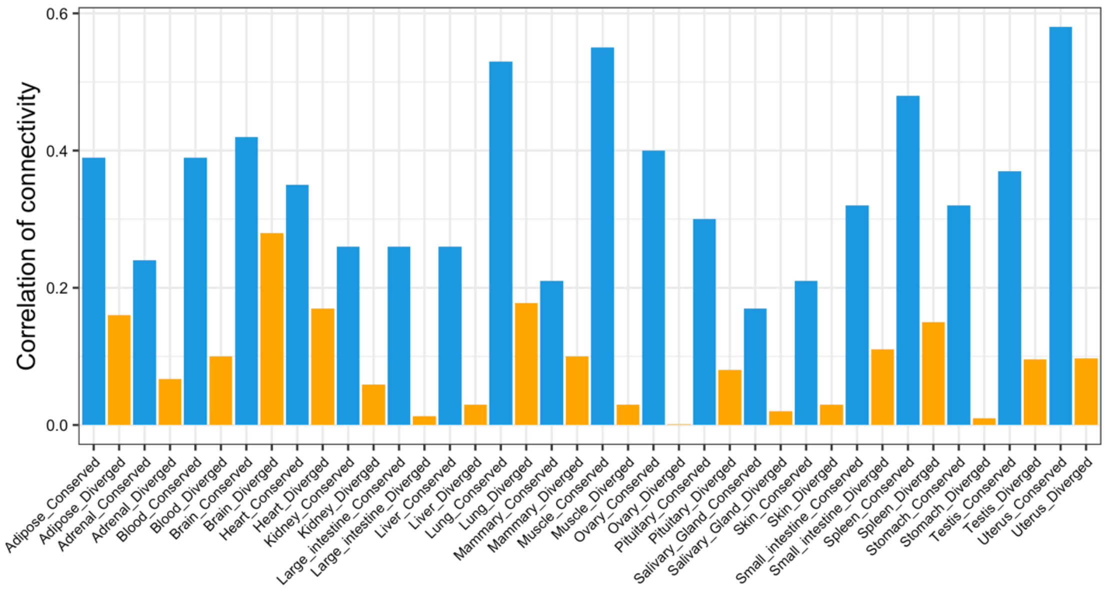

Fig. S14. Correlation of connectivity of the top conserved and diverged modules across 20 tissues between humans and cattle.

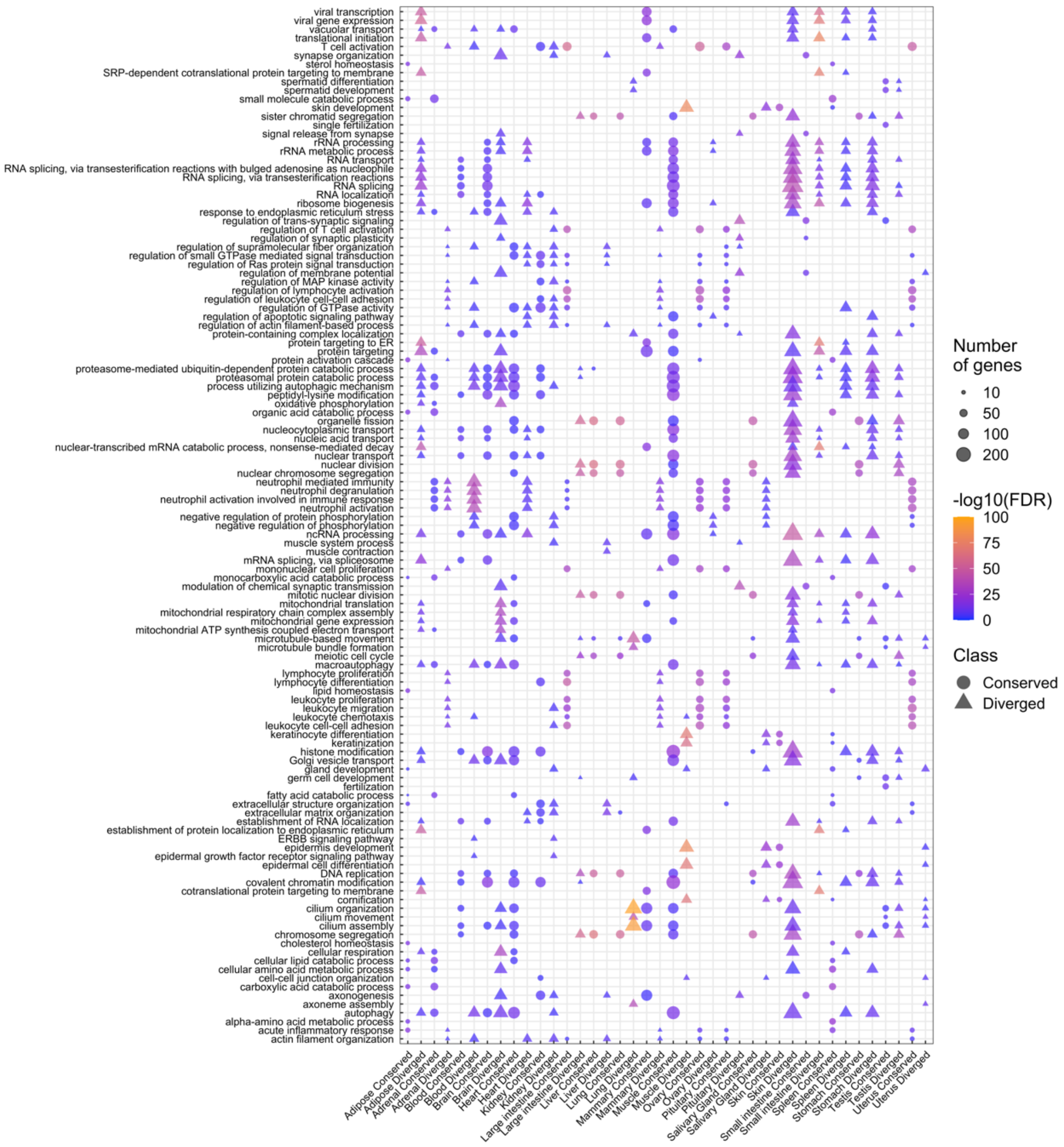

Fig. S15. Enriched Gene Ontology (GO) terms (Biological Process) for the most conserved and diverged modules across 20 tissues between humans and cattle.

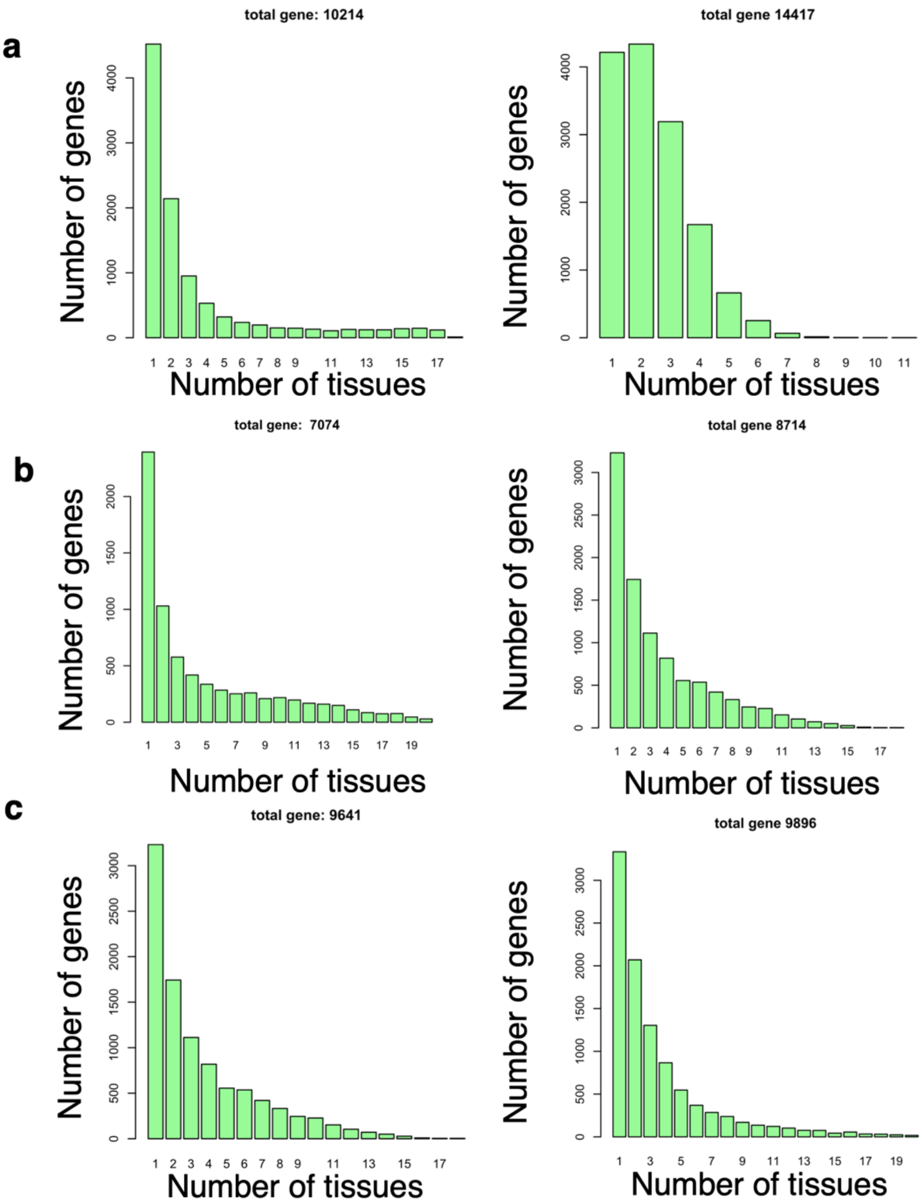

Fig. S16. Distribution of conserved and diverged genes across 20 tissues. a, The distribution of conserved (left) and diverged (right) genes across tissues. Genes are ranked (from largest to smallest) based on degrees (-$\log_{10}p$) of differential expression in each tissue between humans and cattle, while top and last 10% are considered as conserved and diverged genes. **b and c** are for conserved and diverged genes detected from inter-individual variability and gene co-expression, respectively.

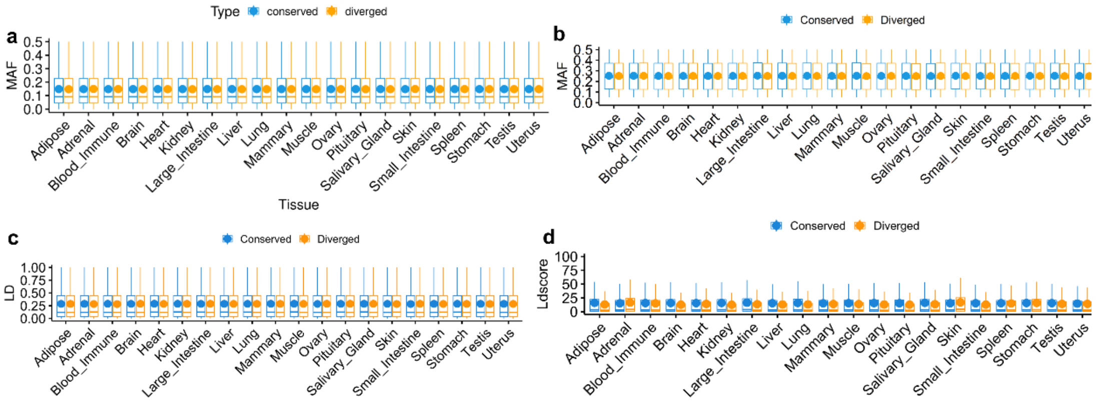

Fig. S17. Comparison of minor allele frequency (MAF) and linkage disequilibrium (LD) of SNPs in conserved and diverged genes. a, Comparison of MAF of LD-independent SNPs within conserved and diverged genes across tissues in cattle, **b**, the same as **a** but for humans. The LD-independent SNPs are obtained using plink (v1.9) (--indep pairwise 1000 5 0.5). **c**, Comparison of LD of SNPs within conserved and diverged genes across tissues in cattle. **d**, Comparison of LDscore (obtained from LDSC) for SNPs within conserved and diverged genes across tissues in humans.

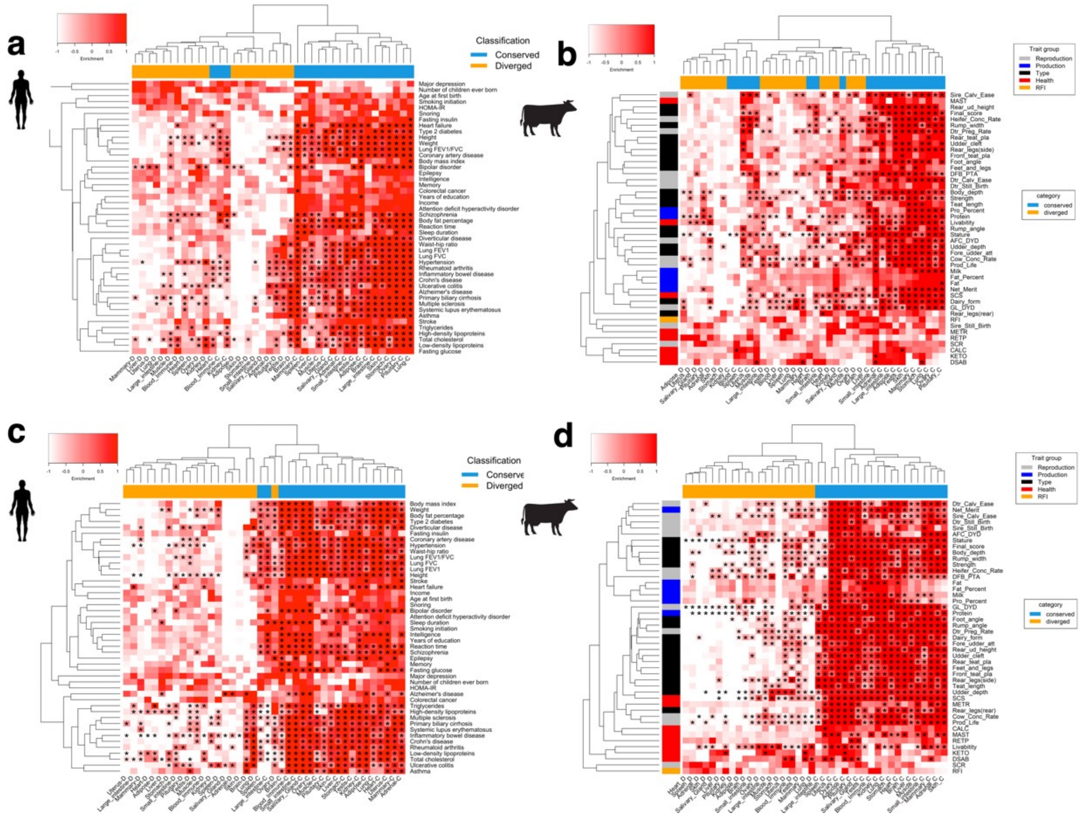

Fig. S18. Heritability enrichment of 46 human complex traits and 45 cattle complex traits in conserved and diverged genes from inter-individual variation and co-expression analysis. a, Heatmap of heritability enrichment estimates

(obtained from LDSC, Methods) of human complex traits for diverged and conserved genes in terms of inter-individual variability between human and cattle. All orthologous genes are ranked (from largest to smallest) based on $-\log_{10}p$ obtained from differential variability analysis. The top and last 10% of genes are considered as diverged and conserved genes, respectively. The enrichment is scaled to have mean of zero and variance of one by traits. "*" represents the adjusted P-value (FDR) < 0.05. Traits and tissues are clustered using Hierarchical clustering methods. **b**, Heatmap of heritability enrichment estimates ($-\log_{10}p$ from 10,000 times permutation, Methods) of cattle complex traits for diverged and conserved genes, which are obtained using the same approach above. The enrichment is scaled to have mean of zero and variance of one by traits. "*" represents FDR < 0.05. **c** and **d** are similar to **a** and **b**, instead for conserved and diverged genes in terms of co-expression conservation in human and cattle.

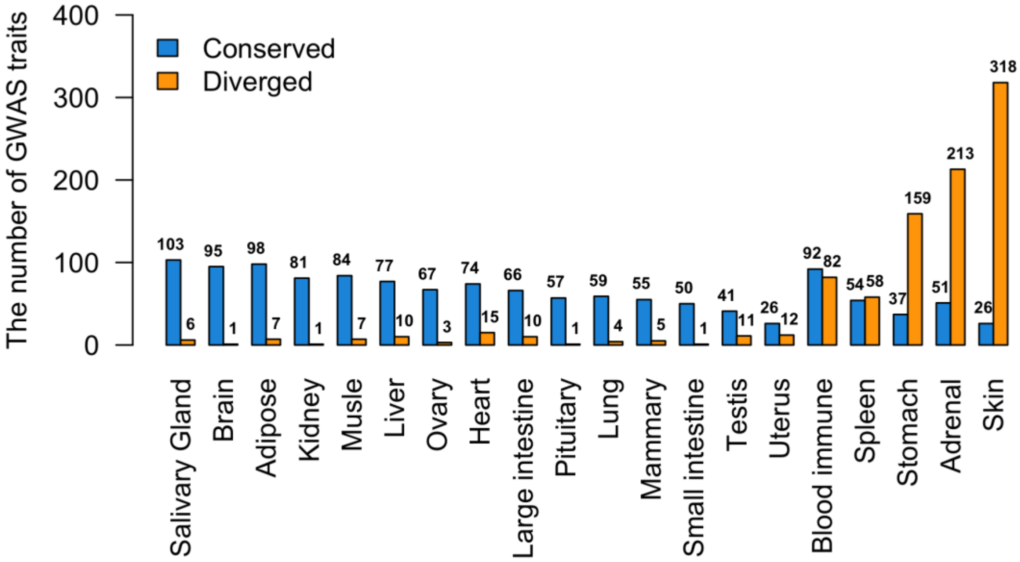

Fig. S19. Bar-plot for the number of GWAS traits significantly enriched (FDR < 0.05) for conserved and diverged genes. All orthologous genes are ranked (from largest to smallest) based on –log10p obtained from differential expression analysis between human and cattle. The bottom 10% and top 10% genes in each tissue are considered as transcriptionally conserved and diverged genes, respectively. The gene-set enrichment analysis, implemented in FUMA (https://fuma.ctglab.nl/), is conducted to test whether the conserved and diverged genes are significantly enriched for genes of each GWAS trait.

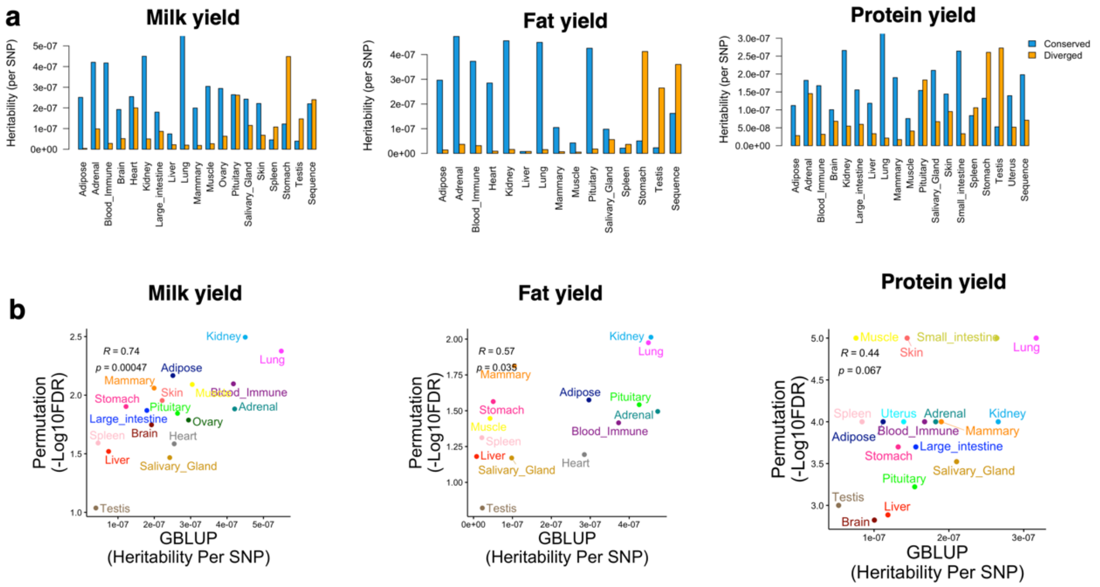

Fig. S20. The per-SNP heritability analysis with GREML-LDMS. **a**, Comparison of the per-SNP heritability between transcriptionally conserved and diverged genes across tissues among milk, fat and protein yield traits. **b,** The Pearson's correlation between -$\log_{10}$FDR from the sum-based permutation test and the per-SNP heritability estimated from the GREML-LDMS models.

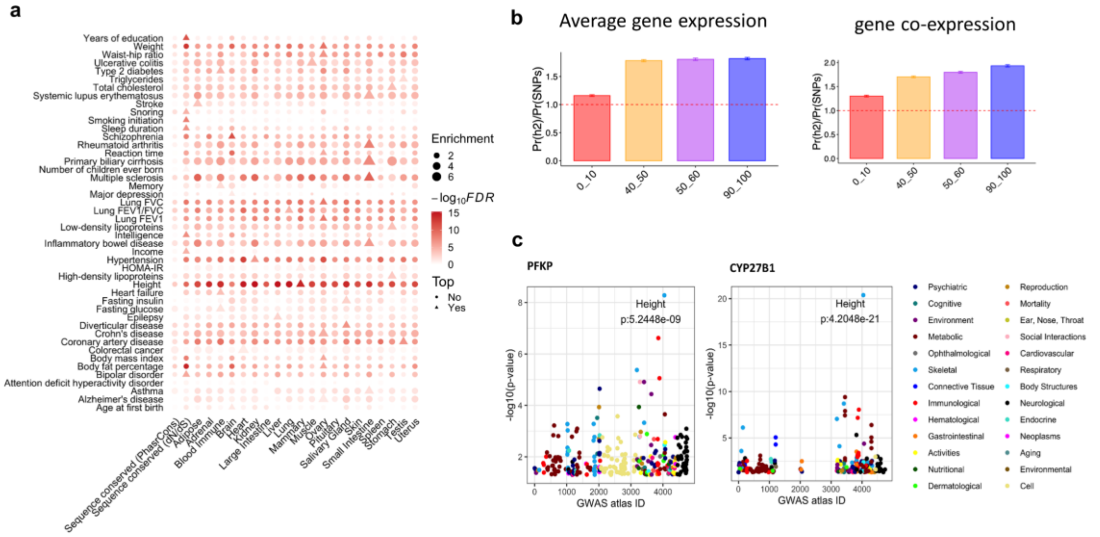

Fig. S21. Transcriptionally conserved genes provide novel insights into the genetics of complex traits. a, The heritability enrichment (Pr(h2g)/Pr(SNPs)) analysis is conducted using LDSC. In each of 20 tissues, genes are ranked (from largest to smallest) based on their corCor scores between humans and cattle (Methods), and then the top 10% of genes are considered as co-expression-conserved genes. The 10% of genes with the smallest dN/dS ratios are considered as DNA sequence conserved. Whereas, the 10% of genes with the largest PhastCons scores are also considered as DNA sequence conserved. **b,** Genes are first ordered by -log10FDR (from largest to smallest) from

differential expression analysis between species. The top 10% (most diverged), 40%-50%, 50%-60% and bottom 10 % (most conserved) of genes are selected in each of 20 tissues for the heritability enrichment analysis of 46 complex human traits using LDSC. The right panel is similar to the left panel, but genes are ordered by corCor scores (from the most diverged to the most conserved). **c**, PheWAS plot for gene *PFKP* and *CYP27B1*. The x-axis represents the -log10 (p-value) of the corresponding gene in a certain GWAS, and y-axis represents the trait ID in GWAS atlas (https://atlas.ctglab.nl/). A total of 4,756 GWAS are considered. Only GWAS traits with Bonferroni corrected P-value < 0.05 are displayed in the plots.

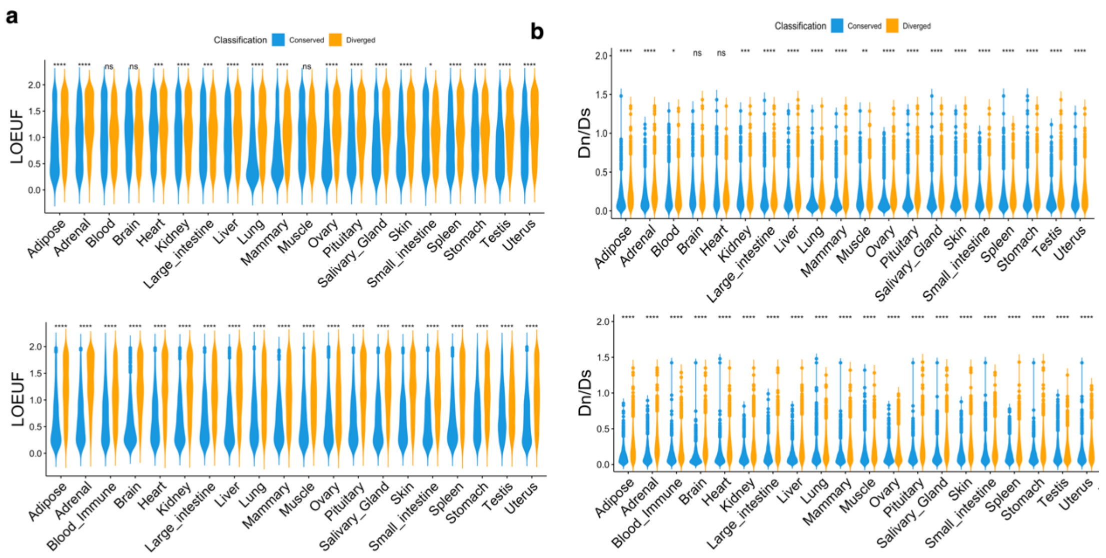

Fig. S22. Comparison of sequence characteristics between conserved and diverged genes. a, Violin plots comparing the LOEUF scores of the diverged and conserved genes across tissues that are defined from inter-individual variability (up) and co-expression analysis (down). **b**, Violin plots comparing the Dn/Ds ratio of diverged and conserved genes across tissues that were defined from inter-individual variability (up) and co-expression analysis (down). Transcriptionally conserved elements generally have significantly smaller LOEUF scores and Dn/Ds ratio than transcriptionally diverged elements.

a

b

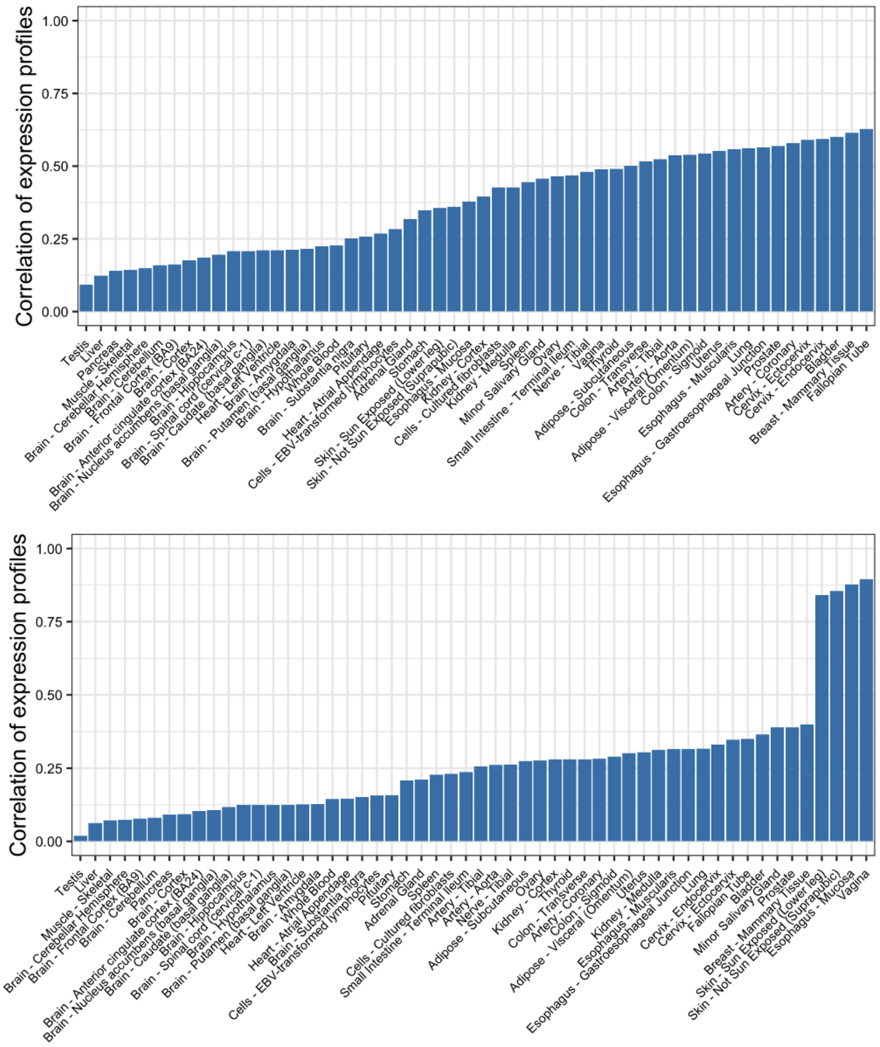

Fig. S23. Bar plot of Pearson's correlation of gene expression between two cattle tissues and human tissues. a, Bar plot of Pearson's correlation of gene expression between cattle horn and 54 human tissues. The Pearson's correlation is calculated based on the median gene expression of individuals between humans and cattle. **b**, same as in **a**, but for cattle rumen.
