# Supplementary material for: Comparative transcriptome in large-scale human and cattle populations
Source: Genome Biol. 2022 Aug 22;23:176. doi: 10.1186/s13059-022-02745-4 (PMC9394047; doi:10.1186/s13059-022-02745-4)
Supplement: Supplementary file 12 — Additional file 12. Peer review history. [file 13059_2022_2745_MOESM12_ESM.docx]

**Review history**

**First round of review**

**Reviewer 1**

Yao et al 2021 studied the conservation of the transcriptome between humans and cattle. The authors have put together a large amount of RNA-seq data. Based on human-cattle ortholog genes, they performed quite some analyses to quantify the extent of the conservation. The analyses ranged from the similarity of gene expression, genes affected by cis-variants to the enrichment of heritability in both humans and cattle traits. The authors show the extent of conservation of gene expression between two species, claimed that the heritability of complex traits in both species are enriched with regions where gene expression are conserved. They also claim that their analysis benefits both biomedicine in humans and genetic improvement in livestock.

I think the topic of the manuscript is of broad interest to others in the field. Also, the amount of data assembled and analyses conducted are impressive. However, I think that the amount of advance of the study over published work is limited, due to the design of the analysis. The conclusions that the work can improve biomedicine and agriculture lacks the support of results. Also, the estimation of enrichment of heritability lacks rigour and this may be a reason for some strange results.

I specify my concerns in the following:

The design of the experiment may bias the conclusions of the paper. The authors analysed data of 17,315 ortholog genes in 20 common tissues between species. It is known that ortholog genes share conservation between species. Also, tissues that are common between species represents similar features between species. If the authors focus on similar features between species, there is a large chance that they are going to find things similar between species. In fact, there are in total 67,000+ human genes and 27,000+ cow genes. So what the authors selected represent ~25% of humans genes and 64% of cow genes. Also, the authors selected 20 tissues in common between species but there are actually 54 human tissues in the human gtex. Therefore, 20 human tissues only represent a small fraction of the total human tissues. This means that what selected here to compare between species may not represent the majority of the features in the two species, with a bias towards similar features.

Previous studies, including recent ones from some of the authors, indicated some degree of the conservation of gene expression between the 2 species (e.g., https://pubmed.ncbi.nlm.nih.gov/32424068/, https://bmcbiol.biomedcentral.com/articles/10.1186/s12915-019-0687-8, https://bmcbiol.biomedcentral.com/articles/10.1186/s12915-020-00792-6). Although, the current study probably provides the most systematic comparison. All these previous studies plus the current one show the conservation of expression of genomic and epigenomic regulators between humans and cows. However, humans and cows are obviously very different. If from genome to epigenome they are so similar, what contributes to their difference? For example, if the authors take genes expressed on horns/tail in cattle and map them into the human genome, where would they be mapped to?

The conclusion is not well supported by the results. The authors concluded 'these findings will benefit both human biomedicine and livestock genetic improvement through leveraging biological information from each other.' I failed to see results to support this claim. As far as I can see, the conservation of the transcriptome was linked to human complex traits and some enrichment of heritability of these complex traits are determined. However, to my understanding, to show that their findings can improve biomedicine, results regarding novel causal genes for human diseases are needed. That is, can authors use the conservation between human and cattle to identify new causal genes for diseases? Also, can the authors show an improved polygenic risk score of human diseases due to the inclusion of mutations within conserved genes between humans and cattle? The same problem is with the cattle. Are there any new mutations for cattle complex traits identified by the conservation between human and cattle? Can conserved genes between humans and cattle improve the selection of productive cows?

It is known that conserved sites across many vertebrates (at the DNA-sequence level) contribute largely and significantly to complex traits. This appears to be true for both human traits (https://www.nature.com/articles/ng.3404) and cattle traits (https://www.pnas.org/content/116/39/19398). Since vertebrates would include humans and cattle, I am not sure if the results of the conservation between cattle and humans are very surprising. However, that being said, the authors have the opportunity to test if the human-cattle conservation at the transcriptomic level is more informative than the conservation at the DNA level. For example, will the transcriptomic conservation outperform sequence conservation in explaining genetic variance, finding additional causal mutations or enhance polygenic sore in humans and/or cattle?

The results of heritability enrichment form an important part of the paper. However, I have concerns regarding the results of correlated heritability between humans and cattle and this may be due to that the estimation of heritability lacks rigour. The author selected 40+ human traits for the analysis and another 40 traits of cows. Human traits selected include many disease traits and cow traits are largely related to milk production. Yet the conclusion is that genetic variance in these two sets of very different traits is more likely to be explained by SNPs within genes showing similar expression pattern between 2 species. This conclusion appears to be hard to accept biologically. Also by looking at some results of correlated heritability enrichment between cow and human traits, they are not very convincing. One of the most studied conserved traits between cattle and human is height (stature in cattle, https://journals.plos.org/plosgenetics/article?id=10.1371/journal.pgen.1008780, https://www.nature.com/articles/s41588-018-0056-5, https://www.ncbi.nlm.nih.gov/pmc/articles/PMC3048786/). However, in the authors' results height doesn't show the correlated heritability enrichment between human and cattle stature (Figure s9). Authors think the correlated heritability enrichment in dairy form and human Alzheimer's disease is expected. I am not sure of this, as 'dairy form' seems to be another measure of milk yield (https://onlinelibrary.wiley.com/doi/full/10.1111/j.1365-2052.2004.01151.x?sid=nlm%3Apubmed). I cant imagine linking a patient with Alzheimer's disease to more milk production…? Are these heritability enrichment results compared against random? For example, if the authors take a random set of SNPs sharing the same LD and MAF property with the targeted SNPs and repeat the analysis, will random SNPs not be enriched in trait heritability?

The heritability estimation lacks rigour. The method used in two species is not unified (LDSC in humans and sum-based test in cattle). The cattle analysis was not based on variance components but rather a GWAS enrichment in different genomic regions. I am not even sure if this method directly estimates the heritability of traits. This is not rigorous. What exactly is the amount of variance in each trait explained by SNPs within conserved and divergent genes? Please provide the actual h2 estimates rather than enrichment statistics (or at least alongside the enrichment results) for different genomic partitions. One of the common metric to compare heritability enrichment is the per-SNP heritability. Can the authors show this metric in 2 species across traits?

The authors cited Liu et al BMC biology 2020, Sørensen Scientific Reports 2017 and Fang Scientific Reports 2017 and said the sum based test is equivalent to LDSC: 'Previous studies showed that results from this method were highly correlated with those from LDSC and other GWAS signal enrichment methods'. In fact, only Liu et al BMC biology 2020 compared the p-value of enrichment of GWAS signals in different genomic regions between the 2 methods. The test statistic was not compared (so the direction of enrichment could be different, even the p-value is small in both methods?) and no simulation was run to compare methods. Sørensen Scientific Reports 2017 used results from GBLUP where marker effects are estimated simultaneously, while the current study applied the method to GWAS where marker effects are tested one at a time.

The authors need to make sure they ruled out potential artefacts that could lead to biased observation regarding the enrichment of heritability. One potential artefact may be that the authors only looked at orthologs. The other potential artefact is likely due to the nonrigorous estimation of heritability. For example, the results may be confounded with the SNP property of MAF and/or LD. That is, SNPs within conserved gene regions maybe also in high MAF and/or high LD, and thus explained more phenotypic variance (common variants explain more genetic variance in traits). These potential artefacts need to be ruled out before conclusions to be made. I suggest the authors consider applying GREML-LDMS (https://www.nature.com/articles/ng.3390) to cow data and apply a similar or comparable approach to human data. This approach allows adjustment of MAF and LD while estimating variance explained by SNPs from divergent and conserved regions. If authors think LD structure needs to be considered, they can also consider LDAK model (http://dougspeed.com/ldak/).
In human GWAS/heritability analysis the sample size is 300K+, but the sample size of cow GWAS is ~ 27,000. It is known that cows have smaller Ne than humans and therefore, there is a large (more than 10 times) difference in sample size, i.e., power, for population-level analysis between the 2 species. Will this bias the enrichment analysis of heritability? It does seem that some of the authors of this manuscript can access larger cattle datasets (https://www.frontiersin.org/articles/10.3389/fgene.2019.00412/full).

Specific comments:
Line 93: 'has an unusual population structure': please define what is unusual?
Line 95: 'large amounts': define large
Line 97-101: '…through genome-wide association studies (GWAS)5,7': GWAS alone has been proven not able to pinpoint causal mutations. It is not clear how GWAS with SNPs in conserved genes can help biomedicine or cattle breeding? Are there any new causal mutations identified due to the conservation between humans and cattle? It is known that conserved regions between vertebrates, which already includes human and cattle, significantly contributed to heritability in both humans and cattle. However, its possible that conserved transcriptomic features between cattle and humans capture more heritability than genomic regions conserved across all vertebrates. However, such a comparison was not done.
Line 165-168: 'human-specific and cattle-specific ones were significantly engaged in cilium organization and synapse assembly, respectively'. But non of these pathways are either human or cattle specific. Can this be artefects due to different gene annotation database between humans and cattle?
Line 185-188: can this be due to the difference in annotation database between humans and cattle? why would human genes upregulated in mammary gland be enriched in 'responses to oxygen level'? what is the biology supporiting this?
Line 191-206: the logic and/or description related to the statement 'genes which tend to be tissue-specific are under purifying selection' is not very clear. If a gene experiences purifying selection, this gene should rather differentially express between humans and cattle. then somehow the authors say the expression of such gene is conserved between humans and cattle, which means the expression is not different between species? Also, with '30 genes with dN/dS ratio > 1', only 14 were showing tissue-specific genes, this is less than half.
Line 221-222: cis eQTLs are selected close to TSS and of course they are found to be centred around TSS (fig 4c). This is again due to biased selection in the feature to compare.
Line 270:272: Figure 6a,b shows the heritability enrichment, not the heritability estimates. Table S6 only shows the human h2 estimates using LDSC baseline model, not partitioned in different genomic regions. No such results for cattle. also Figure 6a,b are bery blurry.
Legend of fig2: TPM cutoff, 0.1 or 1?
Fig3: figure has a-e but the legend has a-f.

**Reviewer 2**

Yao and colleagues performed a large-scale analysis of publicly available RNA-seq data across multiple tissues in cattle and compared with human. They have two major findings. First, they showed that the gene expression was largely conserved between cattle and humans. Second, they found that the heritability of complex traits is significantly enriched in expressed conserved genes. This is very interesting work with clear interpretation and writing. However, there are still some results and analyses that confused me.

1, Lots of comparative studies of transcriptomes from human, and primate or mammal species have been published, which demonstrated that gene expression was conserved during the evolution of these species. So, the one finding that gene expression was largely conserved between cattle and human from their analysis is not surprising. This result might reflect the evolutionary constraint in gene expression in the evolution of human and mammals rather than between human and cattle. I suggest the authors include mice repression data (might be available at ENCODE) into this analysis and perform parallel analysis to measure the gene expression conservation between human and mice as well as between cattle and mice as what they did between human and mice. It would be more interesting to see and analyze that genes with their expression is conserved between human and cattle but diverged between human and mice.

2. It is unclear why muscle transcriptome was selected as an example to show co-expression network conservation between cattle and human, how about other tissues? I think the same analysis should be performed as well for other tissues. It would be interesting to compare the difference in the conservation between gene expression and co-expression network across these tissues between cattle and human.

3. Generally, the method part is just a summary of the method and lacks many details, which is hard to get how the analyses present in the main text were performed, including command and major parameters. Research communities need to use their pipelines to analyze their data and/or repeat these analyses. I also suggest the authors include some proper descriptions for the principle of the method they used before simply listing the result, which will help readers better understand your result.

4. It is unclear why gene expression values were scaled differently among the analysis, some analyses used log2(TPM+0.25) but some used log10(TPM+1). Similarly, some analyses use TPM > 1 to measure the gene that was expressed, but some used TPM >0.1. These are confusing. Please clarify.

5. In the abstract L62, the author said, "We demonstrate that the degree of average gene expression …is generally conserved in humans and cattle". In fact, in their analysis, they used median gene expression level across the tissue, but not average ones? Is there any difference in the results by using average expression level or median expression level across each tissue? Please clarify.

6. L113, "We focused on the expression of 17,315 one-to-one orthologous genes,". Are these protein-coding genes only or both protein-coding and non-coding genes? I think the authors used protein-coding genes, but it is a little confused here. Were genes on the sex chromosome included in this study?

7. L168-169 on page 7: In Fig. 2g, the expression of GABRG2 was higher in the brain than in the testis of cattle, I think it is not a good example of cattle-specific genes to be highlighted here. It would be appreciated if the author could make some biological hypothesis for these specifically expressed genes that they highlighted here.

8. L176-177 on page 7: "As expected, brain and pituitary showed the smallest number of DEGs, while skin and stomach had the largest (Fig. 3a).". It is unclear why it is expected?

9. L179-183 on page 7: "an increased enrichment of active promoter-related states and decreased enrichment of repression-related states in humans when compared to their orthologous genes in cattle, and vice versa for cattle up-regulated DEGs). Please show what is the "active promoter-related states" and "repression-related states" in both figures and the main text.

10. Fig.2b legend, the cutoff (log2 (fold-change) > 1.5; FDR < 0.05) was used to measure tissue-specific genes, it is not clear the power for the cutoff. Is there any reference? Similar to fig.3a legend, it is unclear the power of the cutoff of log2(fold-change, FC) > 1.2 and FDR < 0.05 to calculate the number of significantly up-regulated genes.

**Reviewer 3**

The manuscript by Yuelin Yao and co-authors showcases a very comprehensive comparative functional genomics study between cattle and human populations. The topic falls within the scope of this journal and I believe this work could be relevant to both the readers specialized on mammalian functional genomics, and also to a wider audience with interest on advance methodology for the efficient and effective analysis of large-scale molecular biology datasets. In addition, the manuscript is exceptionally well written using a professional yet vibrant writing style allowing for a enjoyable reading. Because of these reasons, the manuscript deserves eventual publication in a journal the calibre of Genome Biology.
One key concern is that I am not convinced the work has generated significantly new findings benefiting both human biomedicine and livestock genetic improvement. Put another way: What is the new insight that would've been missed by analysing each dataset set in isolation?
It has been long documented that most genes are either tissue-specific or ubiquitously expressed. Also, the kidney of a cow (for argument sake) is more similar to the kidney of a human than it is to the lung of the same cow. Therefore, the finding that "several human traits were significantly correlated with cattle traits across tissues" is no doubt interesting, but hardly ground-breaking.
I believe there is an overemphasis on (and an over-representation of) similarities, and the authors could've explored key physiological differences between cattle and human other than the obvious cornification and skin development (Fig. 5g). One obvious example could be the difference between the digestive systems of ruminants (cattle) vs monogastric (human). Another could be the fact that cattle do not sweat effectively and rely on respiration to cool themselves (and this could be link to the skin development mentioned earlier).
Clearly from Fig. 6a and 6b, the enrichment for conserved genes is much higher than for diverged genes. I wonder if the reason for this over-representation of similarities is a bias generated by selecting only the top and bottom 10% DEG as the diverged and conserved genes, respectively. At the very least an MA-plot is warranted with cattle vs human differential expression on the y-axis is plotted against the average expression on the x-axis.
Also, a further approach to generate new insight could make use of all the genes (not just the top and bottom 10%) and explore the relationship between differential expression and heritability estimates (separate LDSC and permutation for human and cattle). Then focussing on discordances, eg. high differential expression and low heritability.
Having said all that, I consider these concerns a minor defect, not a major flaw. With the proper explanation they are not even a defect. Indeed, I insist that the manuscript is a phenomenal piece of research and I look forward to see it published so that I (like many of my colleagues) can make use of its findings and the available datasets and scripts codes provided therein.

**Authors’ response to reviewers**

**Response to reviewers’ comments:**

**Reviewer #1:**

**General comments:**

**Yao et al 2021 studied the conservation of the transcriptome between humans and cattle. The authors have put together a large amount of RNA-seq data. Based on human-cattle ortholog genes, they performed quite some analyses to quantify the extent of the conservation. The analyses ranged from the similarity of gene expression, genes affected by cis-variants to the enrichment of heritability in both humans and cattle traits. The authors show the extent of conservation of gene expression between two species, claimed that the heritability of complex traits in both species are enriched with regions where gene expression are conserved. They also claim that their analysis benefits both biomedicine in humans and genetic improvement in livestock.

I think the topic of the manuscript is of broad interest to others in the field. Also, the amount of data assembled, and analyses conducted are impressive. However, I think that the amount of advance of the study over published work is limited, due to the design of the analysis. The conclusions that the work can improve biomedicine and agriculture lacks the support of results. Also, the estimation of enrichment of heritability lacks rigour and this may be a reason for some strange results.**

**AU:** Thanks for the valuable comments and suggestions. We have modified the manuscript accordingly. Although many additional analyses have been conducted as suggested by the reviewers, e.g., the functionally informed fine-mapping (i.e., considering transcriptionally conserved genes as prior) analysis *via* PolyFun + SuSiE and the per-SNP heritability analysis *via* GREML-LDMS, we tuned down our conclusion that the current work can improve biomedicine and agriculture (Lines 68-70 in the revised manuscript). This was because that we realized that future studies based on comprehensive computational simulations and experimental functional validations are required to fully address this, which we believe is beyond the scope of the current study.

**I specify my concerns in the following:**
**Q1:** **The design of the experiment may bias the conclusions of the paper. The authors analysed data of 17,315 ortholog genes in 20 common tissues between species. It is known that ortholog genes share conservation between species. Also, tissues that are common between species represents similar features between species. If the authors focus on similar features between species, there is a large chance that they are going to find things similar between species. In fact, there are in total 67,000+ (lnRNA+pro-coding gene, UCSC) human genes and 27,000+ cow genes. So, what the authors selected represent ~25% of humans’ genes and 64% of cow genes.**

**Also, the authors selected 20 tissues in common between species but there are actually 54 human tissues in the human gtex. Therefore, 20 human tissues only represent a small fraction of the total human tissues. This means that what selected here to compare between species may not represent the majority of the features in the two species, with a bias towards similar features.**

**AU:** To ensure the direct comparison of expression of individual genes between species, we had to focus on one-to-one orthologous genes as did by many previous studies on humans and model organisms, which has been considered as a common approach in the comparative transcriptome/epigenome study [1-5]. For instance, Shin et al. conducted a comparison of expression of ~15,106 protein-coding orthologs between humans and mice [3], while Zheng-Bradley et al compared gene expression of 5,925 orthologous genes between humans and mice [4]. Alam et al. used 15,538, 14,915, 13,759, and 8696 one-to-one orthologous genes to conduct a comparative transcriptomics analysis in primary cells between human and mouse, rat, dog, and chicken, respectively [6]. It is difficult to compare the transcription of individual non-orthologous genes between species.

We understood the reviewer’s concerns on the difference in the total number of annotated genes between humans and cattle. This is due to the underrepresentation of non-coding gene annotations in the current cattle genome, and this inconsistency in the quality of genome annotation makes it difficult to distinguish the interspecies differences of non-coding genes*.* According to the latest annotation, 22,796 out of 67,128 genes in humans (GENCODE annotation v38, Ensembl v.104) and 21,861 out of 27,607 genes in cattle (ARS-UCD1.2, Ensembl Genes v.104) are protein-coding genes, which are comparable between these two species. We thus restricted our analysis to 17,315 1-1 unambiguous orthologous genes between humans and cattle, representing 72% and 76% of all annotated protein-coding genes in humans and cattle, respectively. Of note, these orthologous genes contributed to the majority of transcriptional outputs among all 20 tissues being studied between humans and cattle (Figure 1 below), indicating that the non-orthologous genes (the majority were non-coding genes) were often at the very low expression level. Such low expression levels of non-orthologous genes also makes it difficult to compare them between species. In addition, the biological interpretation of results from orthologous protein-coding genes is relatively straightforward due to their well-studied functions. We thus believe that the current results based on these orthologous genes are sufficient enough to reflect the overall difference/similarity in the expression patterns of protein-coding genes between these two species. In the future studies, advanced technologies and approaches are required to fully explore the difference in non-orthologous and non-coding genes between species.

About the selection of samples from the human GTEx project, to facilitate the comparison, we have grouped similar tissues (according to their clustering based on gene expression) into bigger tissue-classes. For instance, we considered different brain regions as brain, as they were clustered together (Figure 2). In total, we analysed 10,830 out of 17,382 RNA-seq samples from the human GTEx project. This resulted in 20 matched tissues between humans and cattle in this study, representing the major primary tissues in these two species. The details of tissue categories are summarized in Table S1 in the revised manuscript. For the unmatched tissues, it was difficult to distinguish whether the difference in expression of orthologous genes was due to differences in the tissue biology or species. Of note, although the global gene expression appeared to be conserved, the expression levels of many individual orthologous genes in the matched tissues were significantly different between humans and cattle (Figure 3). We indeed detected distinct numbers of differentially expressed genes (DEG; FDR < 0.05) across 20 tissues between humans and cattle (Figure 3). For instance, we identified the largest number of DEGs in stomach, skin and testis, while the smallest in brain, heart and muscle. This suggested that the conservation of transcriptome was different across tissues, consistent with that tissues have been proposed as the driver of molecular evolution across mammals [7].

We have discussed the information above in the revised manuscript (Lines 107-109, 119-123, Fig. S1, Table S1).

**Figure 1. The percentage of transcriptional outputs of orthologous and non-orthologous genes.** Genes are classified into orthologues (blue) and non-orthologues (red) in humans and cattle. Orthologous genes generally contributed to a large majority of transcriptional outputs (sequence reads) across 20 tissues being studied.

**Figure 2.** **Heatmap of hierarchical clustering of tissues based on Pearson’s correlation of median gene expression.** There are 54 tissues in humans (including all tissues in the human GTEx project) and 20 tissues in cattle. According to the clustering, the similar human tissues are grouped to 20 bigger tissue-classes (details in the revised Table S1).

**Figure 3**. **Differential gene expression analysis of orthologous genes across 20 matched tissues between humans and cattle**. The red and blue bars correspond to the percentage of all 17,315 1-1 orthologous genes with significantly (Benjamini-Hochberg corrected *P* < 0.05) higher and lower expression in humans compared to cattle, respectively.

**Q2:** **Previous studies, including recent ones from some of the authors, indicated some degree of the conservation of gene expression between the 2 species (e.g.,** [**https://pubmed.ncbi.nlm.nih.gov/32424068/**](https://pubmed.ncbi.nlm.nih.gov/32424068/)**,**[**https://bmcbiol.biomedcentral.com/articles/10.1186/s12915-019-0687-8**](https://bmcbiol.biomedcentral.com/articles/10.1186/s12915-019-0687-8)**,** [**https://bmcbiol.biomedcentral.com/articles/10.1186/s12915-020-00792-6**](https://bmcbiol.biomedcentral.com/articles/10.1186/s12915-020-00792-6)**). Although, the current study probably provides the most systematic comparison. All these previous studies plus the current one shows the conservation of expression of genomic and epigenomic regulators between humans and cows. However, humans and cows are obviously very different. If from genome to epigenome they are so similar, what contributes to their difference? For example, if the authors take genes expressed on horns/tail in cattle and map them into the human genome, where would they be mapped to?**

**AU:** Although the general trend of conservation of gene expression was observed across tissues between humans and cattle, around 50% of orthologous genes were still significantly (FDR < 0.05) differentially expressed in matched tissues between these two species, and the degree of conservation changed across tissues (Figure 3). We found that the conserved genes mainly participate in transcription processing or transcriptional regulation (e.g., RNA splicing, localization or transport), while the diverged genes are involved in the cellular signal transduction (Figure 4). This was in agreement with a previous study that genes peripheral in the regulatory networks (e.g., cellular communication and signal transduction) might contribute more to the phenotypic differences between species compared to other genes [6]. Apart from the gene expression, many other functional elements (e.g., enhancers, ncRNAs, TFBS and translation) and cell type composition might contribute to the difference in phenotypes between species [1, 8, 9]. For instance, many studies have proposed that enhancers evolved much faster across species compared to genes and promoters [8, 10]. In the future study, multi-tissue omics data (e.g., transcriptome, epigenome and proteome), particularly based on the single-cell technology, is highly required to explore the molecular mechanisms underlying the molecular evolution across species.

As suggested by the reviewer, we further compared the gene expression profiles of horns and rumen (the RNA-seq data from tails in cattle were not available) in cattle to those of all 54 tissues in human GTEx (Figure 5-6). We found that, in general, cattle horns showed a low correlation (mean Pearson’s *r* = 0.37) of gene expression across all human tissues, while among them Fallopian Tube was the most similar one (Figure 5, Pearson’s *r* = 0.62). On the other hand, we found that cattle rumen showed the highest similarity with vagina, esophagus and skin in humans compared to other tissues, which was due to the high enrichment of epithelial cells in these tissues (Figure 6).

We have added the information above in the revised manuscript (Lines 210-224, 433-436).

**Figure 4. Functional annotation of transcriptionally conserved and diverged genes between humans and cattle.** The top 10% (blue, conserved) and bottom 10% (orange, diverged) genes in each of 20 tissues are analysed to identify their significantly enriched biological processes based on the Gene Ontology (GO) database. The columns are for tissues, while the rows are for GO terms.

**Figure 5. Bar plot of Pearson’s correlation of gene expression between cattle horn and 54 human tissues.** The Pearson’s correlation is calculated based on the median gene expression of individuals between humans and cattle.

**Figure 6. Bar plot of Pearson’s correlation of gene expression between cattle rumen and 54 human tissues.** The Pearson’s correlation is calculated based on the median gene expression of individuals between humans and cattle.

**Q3:** **The conclusion is not well supported by the results. The authors concluded 'these findings will benefit both human biomedicine and livestock genetic improvement through leveraging biological information from each other.' I failed to see results to support this claim. As far as I can see, the conservation of the transcriptome was linked to human complex traits and some enrichment of heritability of these complex traits are determined. However, to my understanding, to show that their findings can improve biomedicine, results regarding novel causal genes for human diseases are needed. That is, can authors use the conservation between human and cattle to identify new causal genes for diseases? Also, can the authors show an improved polygenic risk score of human diseases due to the inclusion of mutations within conserved genes between humans and cattle? The same problem is with the cattle. Are there any new mutations for cattle complex traits identified by the conservation between human and cattle? Can conserved genes between humans and cattle improve the selection of productive cows?**

**AU:** Although we have added additional analyses below to support this conclusion, we still realized that a comprehensive study based on computational simulations and experimental functional follow-ups are required to fully address all these questions, which we think is beyond the scope of the current study. We thus tuned down this conclusion as also suggested by reviewer #3.

To explore whether transcriptionally conserved/diverged genes were significantly enriched for genes associated with complex traits/diseases in humans, we performed gene-set enrichment analysis based on GWAS-discovered genes of many human complex traits/diseases (at least 10 genes per trait/disease) from GWAS-catalog using GENE2FUNC in FUMA [11] ( <https://fuma.ctglab.nl/>). We found that conserved genes were significantly enriched (FDR < 0.05) for genes of more traits/diseases than diverged genes in most of the tissues, except for skin, adrenal and stomach (Figure 7). This finding further supported the LDSC results and suggested the important roles of transcriptionally conserved genes in complex traits and diseases.

To investigate whether the transcriptional conservation between humans and cattle can help us identify new causal genes for complex traits as suggested by the reviewer, we took the well-studied human height [12] as an example to perform functionally-informed fine-mapping analysis (considering conserved genes as functional priors) using PolyFun + SuSiE [13, 14]. Comparing with results from the fine-mapping analysis without conserved genes (SuSiE only), we fine-mapped additional SNPs and genes consistently at different cutoffs of PIP (Posterior Inclusion Probability) (Figure 8). Considering PIP from PolyFun + SuSiE > 0.95 but PIP from SuSiE only < 0.95, we detected 53 additional variants for human height, and ten of them were not genome-wide significant (*p* = 5e-07) in the original GWAS [12]. Out of these 10 variants, six were mapped to protein-coding genes (Table 1). By conducting the phenome-wide association analysis for these six genes using PheWAS (<https://atlas.ctglab.nl/>), we found that all of them were associated with human height and relevant traits (Table 1). We took *PFKP* and *CYP27B1* as examples in Figure 9.

For the usefulness of conserved genes in the cattle genomic prediction, we compared the FAETH scores of SNPs within conserved and diverged genes in cattle [15]. The FAETH score measures the predictive ability of SNPs for a range of complex traits in dairy cattle. We found that SNPs within conserved genes had significantly higher FAETH scores than those within diverged genes (Wilcox t-test, *p*-value=4.96e-9), consistent across all tissues except for stomach and brain (Figure 10). All these results might suggest that comparative transcriptome between species might provide additional information for the biological interpretation of GWAS loci and genomic prediction.

We have added this information in the revised manuscript (Lines 337-401, Fig. 8b-c, Fig. S19, Table S10, Fig. S21c).

**Figure 7. Bar-plot for the number of GWAS traits significantly enriched (FDR < 0.05) for conserved and diverged genes.** All orthologous genes are ranked (from largest to smallest) based on –log_10_*p* obtained from differential expression analysis between human and cattle. The bottom 10% and top 10% genes in each tissue are considered as transcriptionally conserved and diverged genes, respectively. The gene-set enrichment analysis, implemented in FUMA, is conducted to test whether the conserved and diverged genes are significantly enriched for genes of each GWAS trait.

**Figure 8. Bar-plot for the number of SNPs identified by PolyFun + SuSiE (blue) and SuSiE only (red) for human height at different Posterior Inclusion Probability (PIP) cutoffs.**

**Table 1. Summary information of 10 novel variants detected by PolyFun + SuSiE for human height.**

| SNP | Original GWAS *p* | PIP (PolyFun + SuSiE) | PIP (SuSiE only) | Gene | GWAS atlas (top gene-based *p*) | GWAS atlas trait with the top gene-based *p* |
| --- | --- | --- | --- | --- | --- | --- |
| rs413582 | 3.83E-02 | 0.9521 | 0.0931 | MYBPHL | 2.77E-29 | LDL |
| rs16851441 | 2.87E-06 | 0.9502 | 0.9500 |  |  |  |
| rs17383291 | 3.97E-06 | 0.9608 | 0.0532 |  |  |  |
| rs13220395 | 2.59E-02 | 0.9820 | 0.5042 |  |  |  |
| rs4606023 | 1.34E-01 | 0.9954 | 0.0000 | GSDMC | 5.15E-55 | Height |
| rs4611105 | 5.87E-04 | 0.9904 | 0.0964 | PFKP | 5.24E-09 | Height |
| rs4646537 | 2.61E-05 | 0.9920 | 0.0394 | CYP27B1 | 4.20E-21 | Height |
| rs9323599 | 3.10E-04 | 0.9866 | 0.1187 | NPC2 | 3.04E-13 | Height |
| rs17116589 | 2.76E-05 | 0.9610 | 0.3711 |  |  |  |
| rs12946669 | 2.05E-01 | 0.9796 | 0.0000 | CD79B | 5.25E-34 | Height |

**Figure 9. PheWAS plot for gene *PFKP* and *CYP27B1***. The x-axis represents the -log_10_ (*p*-value) of the corresponding gene in a certain GWAS, and the y-axis represents the trait ID in GWAS atlas (<https://atlas.ctglab.nl/>). A total of 4,756 GWAS are considered. Only GWAS traits with Bonferroni corrected *P*-value < 0.05 are displayed in the plots.

**Figure 10. Comparing FAETH scores of SNPs within transcriptionally conserved and diverged genes.** Transcriptionally conserved and diverged genes refer to the bottom 10% and top 10% genes in the differential expression analysis between cattle and humans, respectively. SNPs within 1000 bp up- and down-stream of gene regions are included. Transcriptionally conserved genes have significantly (Wilcox t-test, *p*-value < 0.05) higher average FAETH scores than diverged ones across tissues except for brain and stomach.

**Q4: It is known that conserved sites across many vertebrates (at the DNA-sequence level) contribute largely and significantly to complex traits. This appears to be true for both human traits (**[**https://www.nature.com/articles/ng.3404**](https://www.nature.com/articles/ng.3404)**) and cattle traits (**[**https://www.pnas.org/content/116/39/19398**](https://www.pnas.org/content/116/39/19398)**). Since vertebrates would include humans and cattle, I am not sure if the results of the conservation between cattle and humans are very surprising. However, that being said, the authors have the opportunity to test if the human-cattle conservation at the transcriptomic level is more informative than the conservation at the DNA level. For example, will the transcriptomic conservation outperform sequence conservation in explaining genetic variance, finding additional causal mutations or enhance polygenic sore in humans and/or cattle?**

**AU:** To test if the human-cattle conservation at the transcriptomic level could provide more information than the conservation at the DNA level, we conducted the heritability enrichment analysis of 47 complex traits for both sequence-conserved genes (i.e., top 10% of genes with the highest sequence conservation between humans and cattle based on their dN/dS ratios from Ensembl annotation) and expression-conserved genes of 20 tissues. As shown in Figure 11, although sequence-conserved genes showed the highest enrichment (denoted as the triangle) for 11 out of 47 traits (e.g., weight and years of education), expression-conserved genes in particular tissues showed the highest enrichment for the remaining 36 traits. For instance, expression-conserved genes in blood showed the highest enrichment for immune/health traits (e.g., Ulcerative colitis, Systemic lupus erythematosus, Rheumatoid arthritis and inflammatory bowel disease). Similar findings were also observed for genes showing conserved co-expression patterns (Figure 12). For example, we found that genes with conserved co-expression in brain showed the highest enrichment for Schizophrenia, while genes in small intestine for immune-relevant traits (e.g., rheumatoid arthritis and inflammatory bowel disease,) might be due to its immune function. Although several trait-tissue associations were not straightforward due to the complexity in both traits and tissues, these results indicate that the transcriptome conservation in relevant tissues might provide additional information for interpreting complex trait genetics compared to the DNA sequence conservation. Regarding how to leverage this transcriptomic level information for identifying causal mutations and genes for complex traits, please see the responses to Q3.

We have added this information in the revised manuscript (Lines 360-378, Fig. 8a, Fig. S21a).

**Figure 11. Heritability enrichment of 47 human complex traits for expression-conserved genes and sequence-conserved genes.** The heritability enrichment (Pr(h^2^_g_)/Pr(SNPs)) analysis is conducted using LDSC. In each of 20 tissues, we rank genes (from largest to smallest) based on –log_10_*p* from differential expression analysis between humans and cattle and then consider the bottom 10% genes as expression-conserved genes. We rank genes based on their dN/dS ratios (from smallest to largest) between humans and cattle and then consider the top 10% of genes as sequence-conserved genes.

**Figure 12. Heritability enrichment of 47 human complex traits for co-expression-conserved genes and sequence-conserved genes.** The heritability enrichment (Pr(h2g)/Pr(SNPs)) analysis is conducted using LDSC. In each of 20 tissues, genes are ranked (from largest to smallest) based on their corCor scores between humans and cattle (Methods), and then the top 10% of genes are considered as co-expression-conserved genes. Genes are ranked based on their dN/dS ratios (from smallest to largest) between humans and cattle, and then the top 10% of genes are considered as sequence-conserved genes.

**Q5: The results of heritability enrichment form an important part of the paper. However, I have concerns regarding the results of correlated heritability between humans and cattle and this may be due to that the estimation of heritability lacks rigour. The author selected 40+ human traits for the analysis and another 40 traits of cows. Human traits selected include many disease traits and cow traits are largely related to milk production. Yet the conclusion is that genetic variance in these two sets of very different traits is more likely to be explained by SNPs within genes showing similar expression pattern between 2 species. This conclusion appears to be hard to accept biologically. Also, by looking at some results of correlated heritability enrichment between cow and human traits, they are not very convincing. One of the most studied conserved traits between cattle and human is height (stature in cattle,** [**https://www.nature.com/articles/s41588-018-0056-5**](https://www.nature.com/articles/s41588-018-0056-5)**,** [**https://www.ncbi.nlm.nih.gov/pmc/articles/PMC3048786/**](https://www.ncbi.nlm.nih.gov/pmc/articles/PMC3048786/)**). However, in the authors' results height doesn't show the correlated heritability enrichment between human and cattle stature (Figure s9). Authors think the correlated heritability enrichment in dairy form and human Alzheimer's disease is expected. I am not sure of this, as 'dairy form' seems to be another measure of milk yield (**[**https://onlinelibrary.wiley.com/doi/full/10.1111/j.1365-2052.2004.01151.x?sid=nlm%3Apubmed**](https://onlinelibrary.wiley.com/doi/full/10.1111/j.1365-2052.2004.01151.x?sid=nlm%3Apubmed)**). I can’t imagine linking a patient with Alzheimer's disease to more milk production…? Are these heritability enrichment results compared against random? For example, if the authors take a random set of SNPs sharing the same LD and MAF property with the targeted SNPs and repeat the analysis, will random SNPs not be enriched in trait heritability?**

**AU**: We agree with the reviewer and notice that it is difficult to interpret the correlation of heritability enrichment between human and cattle traits across tissues, due to both the small number (n = 20) of tissues being analysed and the heterogeneity of complex traits between these two species. Therefore, we removed the heritability enrichment correlation part from the revised manuscript. Although we analysed 45 complex traits in dairy cattle, including reproduction (n = 12), production (milk-relevant; n = 6), body conformation (n = 18), health (immune/metabolic-relevant; n = 8) and one feed efficiency trait (i.e., residual feed intake, RFI), it was difficult to find the physiologically or biologically matched traits between humans and cattle. About the similarity between Alzheimer's disease and milk yield, based on the FUMA results, we found that expression-conserved genes detected in mammary gland were significantly enriched for GWAS-discovered genes of human brain-related diseases such as bipolar, Schizophrenia and Alzheimer's disease. This might support the observed heritability enrichment correlation between milk yield in cattle and Alzheimer’s disease in humans, since Alzheimer's disease and milk yield are both somehow related to brain functions [16]. However, we did not find a significant heritability enrichment correlation between human height and cattle stature across these 20 tissues.

**Q6: The heritability estimation lacks rigour. The method used in two species is not unified (LDSC in humans and sum-based test in cattle). The cattle analysis was not based on variance components but rather a GWAS enrichment in different genomic regions. I am not even sure if this method directly estimates the heritability of traits. This is not rigorous. What exactly is the amount of variance in each trait explained by SNPs within conserved and divergent genes? Please provide the actual h2 estimates rather than enrichment statistics (or at least alongside the enrichment results) for different genomic partitions. One of the common metric to compare heritability enrichment is the per-SNP heritability. Can the authors show this metric in 2 species across traits?**

**AU:** As suggested by the reviewer, we calculated the per-SNP heritability for conserved and diverged genes in cattle using the three-component (i.e., SNPs within conserved, diverged and the rest of genome) GREML-LDMS model [17], as did by Xiang et al., [15]. We took three milk production traits (i.e., milk, fat and protein yield) as examples, as they had the largest sample size (n = 27,235) and the highest reliability of phenotypes [14]. Briefly, we computed the amount of genetic variance in each trait explained by the three groups of SNPs, i.e., conserved genes, divergent genes and the rest of genome. We then calculated the per-SNP heritability for each group by adjusting for their SNP sizes. We only considered autosomal variants with MAF > 5% (n = 3,085,572 SNPs) in this analysis. However, the models were not converged properly for several tissues in each of the three traits. We found that expression-conserved genes had the higher per-SNP heritability than DNA sequence-conserved genes and expression-diverged genes across most of the tissues (Figure 13a). We also observed that the enrichment degrees of GWAS signals based on the sum-based permutation test were significantly correlated with the per-SNP heritability across tissues for milk and fat yield but not protein yield (Figure 13b). Compared to GREML-LDMS models, the sum-based permutation test based on the summary statistics was computationally faster, which could be used to scan a large number of genomic features in a short time [18].

The details of the explained variance and heritability of cattle complex traits are summarized in the revised Table S9, while the corresponding information based on LDSC for human traits is updated in the revised Table S8. We have added this information in the revised manuscript (Lines 348-359, Table S8-9, Fig. S20).

**Figure 13. The per-SNP heritability analysis with GREML-LDMS**. **a**, Comparison of the per-SNP heritability between transcriptionally conserved and diverged genes across tissues among milk, fat and protein yield traits. **b,** The Pearson’s correlation between -log_10_FDR from the sum-based permutation test and the per-SNP heritability estimated from the GREML-LDMS models.

**Q7: The authors cited Liu et al BMC biology 2020, Sørensen Scientific Reports 2017 and Fang Scientific Reports 2017 and said the sum-based test is equivalent to LDSC: 'Previous studies showed that results from this method were highly correlated with those from LDSC and other GWAS signal enrichment methods'. In fact, only Liu et al BMC biology 2020 compared the p-value of enrichment of GWAS signals in different genomic regions between the 2 methods. The test statistic was not compared (so the direction of enrichment could be different, even the p-value is small in both methods?) and no simulation was run to compare methods. Sørensen Scientific Reports 2017 used results from GBLUP (raw genotype) where marker effects are estimated simultaneously, while the current study applied the method to GWAS where marker effects are tested one at a time.**

**AU:** As suggested by the reviewer, we have run the three-component GREML-LDMS model to estimate the per-SNP heritability for SNPs within conserved and diverged genes, as did by Xiang et al., (2019) [15]. We then compared the per-SNP heritability with the enrichment degree (i.e., -log_10_FDR) from the sum-based permutation test (Figure 13b). Please see details in our response to Q6 above. In addition, LDSC does not work well in the population with related individuals (e.g., cattle population) (<https://www.biorxiv.org/content/biorxiv/early/2021/10/13/2021.10.10.463846.full.pdf>). As suggested by the reviewer, it will be of interest to conduct a benchmark study to compare different heritability enrichment methods based on both comprehensive computational simulations and real data in the future studies.

**Q8: The authors need to make sure they ruled out potential artefacts that could lead to biased observation regarding the enrichment of heritability. One potential artefact may be that the authors only looked at orthologs. The other potential artefact is likely due to the nonrigorous estimation of heritability. For example, the results may be confounded with the SNP property of MAF and/or LD. That is, SNPs within conserved gene regions maybe also in high MAF and/or high LD, and thus explained more phenotypic variance (common variants explain more genetic variance in traits). These potential artefacts need to be ruled out before conclusions to be made. I suggest the authors consider applying GREML-LDMS (**[**https://www.nature.com/articles/ng.3390**](https://www.nature.com/articles/ng.3390)**) to cow data and apply a similar or comparable approach to human data. This approach allows adjustment of MAF and LD while estimating variance explained by SNPs from divergent and conserved regions. If authors think LD structure needs to be considered, they can also consider LDAK model (**[**http://dougspeed.com/ldak/**](http://dougspeed.com/ldak/)**).**

**AU:** As suggested by the reviewer, we have applied the GREML-LDMS model to estimate the per-SNP heritability for three milk production traits and then compared it with the results from the sum-based permutation test. Please see details in our response to Q6 above. In addition, we compared the MAF and LD of SNPs within conserved and diverged genes, and found no significant differences in MAF or LD between these two groups of genes across tissues in humans or cattle (Figure 14). Therefore, MAF and LD might not bias the results.

We have added this information in the revised manuscript (Lines 337-338, Fig. S17).

**Figure 14. Comparison of MAF and LD of SNPs in conserved and diverged genes**. **a,** Comparison of MAF of SNPs within conserved and diverged genes across tissues in cattle, **b**, the same as **a** but for human. **c**, Comparison of LD of SNPs within conserved and diverged genes across tissues in cattle (using PLINK v.1.9). **d,** Comparison of LDscore (obtained from LDSC) for SNPs within conserved and diverged genes across tissues in humans.

**Q9: In human GWAS/heritability analysis the sample size is 300K+, but the sample size of cow GWAS is ~ 27,000. It is known that cows have smaller Ne than humans and therefore, there is a large (more than 10 times) difference in sample size, i.e., power, for population-level analysis between the 2 species. Will this bias the enrichment analysis of heritability? It does seem that some of the authors of this manuscript can access larger cattle datasets (**[**https://www.frontiersin.org/articles/10.3389/fgene.2019.00412/full**](https://www.frontiersin.org/articles/10.3389/fgene.2019.00412/full)**).**

**AU:** In this study, the phenotypes of bulls we analysed are DE-regressed PTA (breeding values), which are calculated based on hundreds and thousands of phenotypic records from the relatives (e.g., their daughters), particularly for milk production traits. Therefore, the accuracy or reliability of phenotypes in bulls is much higher than those in cows. The high accuracy in phenotypic measurement, to a great extent, compensate for the reduction of GWAS power due to the smaller sample size. Our previous estimation showed that the power of GWAS (N ~ 300K) was similar between this Holstein bull population and the large cow population analysed previously [19] (<https://www.frontiersin.org/articles/10.3389/fgene.2019.00412/full>). Therefore, our cattle GWAS used in this study has comparable power as human GWAS (both have an effective sample size of 300K+). The genotypes for our cow population have not been imputed to the whole genome sequence level, which makes it difficult to link enough SNPs to a particular gene.

**Specific comments:**
**Line 93: 'has an unusual population structure': please define what is unusual?**

**AU**: Compared to humans, cattle (e.g., Holstein and Angus) has a much smaller effective population size (Ne ~100), a larger linkage disequilibrium (LD) among genomic variants, and a higher inbreeding rate (i.e., resulting in the accumulation of deleterious mutations) partially due to the intensive artificial selection for complex traits of economic value (e.g., milk yield) [20, 21]. We have added this information in the revised manuscript (Lines 95-99).

**Line 95: 'large amounts': define large**

**AU**: Millions of highly accurate phenotypic records, including fertility, health and growth traits, have been collected for the genomic improvement in cattle. For instance, over five million cows and bulls have been genotyped and phenotyped in the U.S.A dairy cattle population (<https://www.uscdcb.com/wp-content/uploads/2021/10/2021-Activity-Report_101921_lowres.pdf>). We have added this information in the revised manuscript (Lines 99-101).

**Line 97-101: '…through genome-wide association studies (GWAS)5,7': GWAS alone has been proven not able to pinpoint causal mutations. It is not clear how GWAS with SNPs in conserved genes can help biomedicine or cattle breeding? Are there any new causal mutations identified due to the conservation between humans and cattle? It is known that conserved regions between vertebrates, which already includes human and cattle, significantly contributed to heritability in both humans and cattle. However, its possible that conserved transcriptomic features between cattle and humans capture more heritability than genomic regions conserved across all vertebrates. However, such a comparison was not done.**

**AU**: As suggested by the reviewer, through including the expression-conserved genes as biological priors in the fine-mapping model (PloyFun+SuSiE) [13], we detected several new genetic variants and genes for human height (Table 1 above). Please find the details in the responses to Q3-4 above. However, the experimental functional follow-ups are required to further confirm whether they are really causal for height and the underlying mechanisms. Thus, we have turned down this conclusion in the revised manuscript. We have also compared sequence-conserved genes and expression-conserved ones in terms of the heritability enrichment of complex traits and found that expression-conserved genes in certain tissues showed higher enrichment for relevant traits than sequence-conserved genes (Figure 11-12 and Figure 13a).

We have added this information in the revised manuscript (Lines 359-377).

**Line 165-168: 'human-specific and cattle-specific ones were significantly engaged in cilium organization and synapse assembly, respectively'. But none of these pathways are either human or cattle specific. Can this be artefects due to different gene annotation database between humans and cattle?**

**AU**: Yes, this might be biased by the difference in gene annotations between humans and cattle, since genes specific in cattle might not be well studied and functionally annotated. We have added this information in the revised manuscript (Lines 180-181).

**Line 185-188: can this be due to the difference in annotation database between humans and cattle? why would human genes upregulated in mammary gland be enriched in 'responses to oxygen level'? what is the biology supporting this?**

**AU**: Yes, we agree with the reviewer that the difference in gene annotation databases between humans and cattle might bias the interpretation of human- and cattle-specific genes. From pregnancy to lactation, the mammary gland exhibits an increased metabolic rate to support mammary growth, lactogenesis, and lactation, which requires an increasing amount of oxygen consumption [22]. The oxygen level is thus important for supporting the increased metabolic rate during pregnancy and lactation in mammary gland [22]. In cattle, due to the high intensive selection on milk production or mammary gland health traits (e.g., mastitis), the oxygen level in milk might be co-selected, which might result in the down-regulation of the relevant genes in the cattle mammary gland [23, 24]. However, comprehensive experimental functional follow-ups are required to further understand the molecular mechanisms underlying this.

We have added this information in the revised manuscript (Lines 202-209).

**Line 191-206: the logic and/or description related to the statement 'genes which tend to be tissue-specific are under purifying selection' is not very clear. If a gene experiences purifying selection, this gene should rather differentially express between humans and cattle. then somehow the authors say the expression of such gene is conserved between humans and cattle, which means the expression is not different between species? Also, with '30 genes with dN/dS ratio > 1', only 14 were showing tissue-specific genes, this is less than half.**

**AU**: We have rephrased the statement to make it clear in the revised manuscript (Lines 243-245, 251-253).

In general, purifying selection or negative selection is the selective removal of deleterious genetic variants that arise from random mutations and reduce genetic diversity, while positive selection promotes the presence of new phenotypes [25, 26]. Therefore, the expression levels of genes under purifying selection are likely to be conserved between species [27, 28]. In most of the tissues except for mammary gland, stomach and skin, we found that genes with higher tissue-specific expression exhibited higher levels of expression conservation between humans and cattle (Figure 3d). This is in line with that the main biological functions of many tissues (reflected by the expression of tissue-specific genes) are conserved between these two species. By saying “*many of these genes (14 out of 30) showed tissue-specific expression patterns and DEGs in at least one tissue between human and cattle*”, we meant that these 14 genes exhibited both tissue-specific expression and significantly differential expression between humans and cattle. In fact, 26 out of 30 genes showed tissue-specific expression (log_2_FC >1.5, FDR < 0.05) in at least one tissues.

**Line 221-222: cis eQTLs are selected close to TSS and of course they are found to be centred around TSS (fig 4c). This is again due to biased selection in the feature to compare.**

**AU**: When conducting cis-eQTL mapping, we only considered SNPs with ± 1Mb of TSS. However, all tested SNPs were uniformly distributed around TSS, and only top cis-QTLs of genes were centred around TSS. This was consistent in cattle (Figure 16a) and humans (Figure 16b, adopted from [29]). However, we found that top cis-eQTLs in humans had a higher concentration around TSS than those in cattle, might be partially due to the difference in LD patterns between these two species. For instance, we found that 50% of top cis-eQTLs were located within 150 kb and 30 kb around TSS in cattle and humans, respectively (Figure 16c-d).

We have added this information in the revised manuscript (Lines 272-278, Fig. S12).

**Figure 15. The distribution of top cis-eQTLs around TSS in cattle and humans.** **a,** Distribution of cis-eQTLs around TSS (1Mb up- and down-stream) in adipose. All indicates all tested SNPs; Sig indicates the top cis-eQTLs for significant eGenes; No_sig indicates the top associated SNPs for non-significant genes (non-eGenes). **b,** Distribution of top cis-eQTLs around the TSS (1Mb up- and down-stream) in human GTEx (adopted from [29]). **c,** Cumulative distribution of top cis-eQTLs to TSS across cattle tissues. **d,** Cumulative distribution of top cis-eQTLs to the TSS across human tissues (obtained from <https://gtexportal.org/home/datasets>).

**Line 270:272: Figure 6a,b shows the heritability enrichment, not the heritability estimates. Table S6 only shows the human h2 estimates using LDSC baseline model, not partitioned in different genomic regions. No such results for cattle. also Figure 6a, b are bery blurry.**

**AU:** To make the original Figure 6a-b easy to read, we have separated them into two individual Figures (i.e., the revised Figure 6 and 7) in the revised manuscript. We have provided h^2^ estimates and the heritability enrichment in each genomic region for humans and cattle in the revised Table S8 and S9, respectively.

**Legend of fig2: TPM cutoff, 0.1 or 1?
Fig3: figure has a-e but the legend has a-f.**

**AU**: Corrected. We defined gene with TPM > 0.1 as expressed, and modified the legends of Fig. 2 and 3 carefully in the revised manuscript.

**Reviewer #2**:

**Yao and colleagues performed a large-scale analysis of publicly available RNA-seq data across multiple tissues in cattle and compared with human. They have two major findings. First, they showed that the gene expression was largely conserved between cattle and humans. Second, they found that the heritability of complex traits is significantly enriched in expressed conserved genes. This is very interesting work with clear interpretation and writing. However, there are still some results and analyses that confused me.**

**Q1, Lots of comparative studies of transcriptomes from human, and primate or mammal species have been published, which demonstrated that gene expression was conserved during the evolution of these species. So, the one finding that gene expression was largely conserved between cattle and human from their analysis is not surprising. This result might reflect the evolutionary constraint in gene expression in the evolution of human and mammals rather than between human and cattle. I suggest the authors include mice expression data (might be available at ENCODE) into this analysis and perform parallel analysis to measure the gene expression conservation between human and mice as well as between cattle and mice as what they did between human and cattle. It would be more interesting to see and analyse that genes with their expression is conserved between human and cattle but diverged between human and mice.**

**AU**: Thanks for the valuable suggestions. We have collected and analysed 113 mice RNA-seq samples from 14 tissues (obtained from <http://rna.recount.bio/>) [3, 30] together with human and cattle RNA-seq data. In general, we found that gene expression profiles for most of the tissues are conserved among the three mammals (Figure 16a). However, as expected, cattle stomach (mainly rumen) was different from human and mice stomach (monogastric). We conducted the differential expression analyses of orthologous genes in the matched tissues between humans and mice, and compared them with results from humans *vs.* cattle. In general, the *t*-statistics (measuring the difference in gene expression) from humans *vs*. mice were significantly but moderately correlated with those from humans *vs.* cattle (Figure 16b-c). These results suggest although genes that were conserved between humans and cattle were largely overlapped with those between humans and mice, there were still a fraction of genes that were specifically conserved between humans and cattle. We then detected genes that showed conservation (|Fold Change| < 1.2 and FDR > 0.05) between humans and cattle but divergence (|Fold Change| > 1.2 and FDR < 0.05) between humans and mice in each of 14 tissues being studied (Figure 16d). The functional enrichment analysis based on Gene Ontology (GO) showed that those genes were significantly engaged in distinct biological processes (Figure 17). For instance, those genes in adipose, spleen, lung and mammary gland were significantly enriched for immune systems, such as T cell activation and regulation of lymphocyte proliferation (Figure 17). This might suggest that cattle show a greater similarity to humans than mice in terms of several aspects of immunophysiology, which was in agreement with previous studies [31, 32]. We also noticed that those genes in heart and liver were significantly involved in muscle contraction, ATP processing and glucose metabolism, which might be in line with that cattle has been proposed as a model for some muscular disorders, e.g., brody disease [33].

We have added this information in the revised manuscript (Lines 224-239, Fig. S8).

**Figure 16. Comparative transcriptome across human, cattle and mouse.** **a**, Heatmap of hierarchical clustering based on Pearson’s correlation of median expression of 14,490 orthologous genes. **b**, Barplot showing the correlation between the *t*-statistics (measuring the difference in gene expression) of humans *vs*. mice and those of humans *vs*. cattle. **c**, The scatter plot for *t*-statistics of humans *vs*. mice and those of humans *vs*. cattle in brain. **d**, Bar plot for the numbers of genes with conserved expression (|Fold Change| < 1.2 and FDR > 0.05) between humans and cattle but diverged expression between humans and mice (|Fold Change| > 1.2 and FDR < 0.05).

**Figure 17. Enriched GO terms (Biological Process) for genes that are transcriptionally conserved in humans *vs*. cattle but diverged in humans *vs*. mice.**

**Q2. It is unclear why muscle transcriptome was selected as an example to show co-expression network conservation between cattle and human, how about other tissues? I think the same analysis should be performed as well for other tissues. It would be interesting to compare the difference in the conservation between gene expression and co-expression network across these tissues between cattle and human.**

**AU**: We chose muscle as an example to show the conservation of gene co-expression modules detected from WGCNA analysis [34], because muscle was the tissue with the highest global conservation of gene co-expression based on the Corcor score [35] (Fig. 5c in the revised manuscript). We have performed WGCNA for all 20 tissues to detect gene co-expression modules and assess their conservation between humans and cattle. In each tissue, we summarized the top conserved and divergent modules and their corresponding functional annotations in Figure 18 and 19, respectively. We found that these genes in different tissues were significantly enriched in distinct biological functions. For instance, genes of the most diverged module in blood were significantly enriched in neutrophil-mediated immunity, while genes of the most diverged module in brain were significantly enriched in mitochondrial ATP functions. In addition, we compared the conservation between gene expression and co-expression, and found that expression-conserved genes showed significantly (Wilcoxon test, p-value < 2.2e-16) higher co-expression conservation than expression-diverged genes (Figure 20).

We have added this information in the revised manuscript (Lines 305-311, 318-324, Fig. S13-15).

**Figure 18. Correlation of connectivity of the top conserved and diverged modules across 20 tissues between humans and cattle.**

**Figure 19. Enriched GO terms (Biological Process) for genes within the most conserved and diverged module across 20 tissues between humans and cattle.**

**Figure 20. Comparison of CorCor scores between expression-conserved and -diverged genes.** Conserved and diverged genes represent the last (conserved) and top (diverged) 10% of all orthologous genes ordered by -log_10_FDR from the differential expression analysis in each of 20 tissues between humans and cattle.

**Q3, Generally, the method part is just a summary of the method and lacks many details, which is hard to get how the analyses present in the main text were performed, including command and major parameters. Research communities need to use their pipelines to analyse their data and/or repeat these analyses. I also suggest the authors include some proper descriptions for the principle of the method they used before simply listing the result, which will help readers better understand your result.**

**AU**: We have added details of methods in the revised manuscript.

**Q4, It is unclear why gene expression values were scaled differently among the analysis, some analyses used log2(TPM+0.25) but some used log10(TPM+1). Similarly, some analyses use TPM > 1 to measure the gene that was expressed, but some used TPM >0.1. These are confusing. Please clarify.**

**AU**: We have checked and corrected all of these carefully to keep them consistent throughout the entire manuscript. The gene expression values were scaled as log_2_(TPM + 0.25), and genes with TMP > 0.1 were defined as expressed.

**Q5, In the abstract L62, the author said, "We demonstrate that the degree of average gene expression …is generally conserved in humans and cattle". In fact, in their analysis, they used median gene expression level across the tissue, but not average ones? Is there any difference in the results by using average expression level or median expression level across each tissue? Please clarify.**

**AU**: We compared the tissue-pair correlations based on both average and median gene expression (Figure 21a), and found that they were highly and significantly correlated (*r* = 0.99 and p < 2.2e-16). We have changed ‘average’ to ‘mean/median’ in the revised manuscript (Line 61), and added this information in the revised manuscript (Lines 132-134, Fig. S2a-b).

**Figure 21.** **a**, Heatmap of hierarchical clustering of tissues based on Pearson’s correlation of average gene expression. **b**, The scatter plot for tissue-pair correlations based on mean (y-axis) and median (x-axis) gene expression.

**Q6, L113, "We focused on the expression of 17,315 one-to-one orthologous genes,". Are these protein-coding genes only or both protein-coding and non-coding genes? I think the authors used protein-coding genes, but it is a little confused here. Were genes on the sex chromosome included in this study?**

**AU**: Out of these 17,315 one-to-one orthologous genes, 16,510 are protein-coding genes with 664 on the sex chromosome. We have added these details in the revised manuscript (Lines 118-122).

**Q7, L168-169 on page 7: In Fig. 2g, the expression of GABRG2 was higher in the brain than in the testis of cattle, I think it is not a good example of cattle-specific genes to be highlighted here. It would be appreciated if the author could make some biological hypothesis for these specifically expressed genes that they highlighted here.**

**AU**: As suggested by the reviewer, we have replaced *GABRG2* with *DAZ1* in the revised Fig. 2g*,* which was highly and specifically expressed in the cattle testis. *DAZ1* is essential for spermatogenesis and its variants have been associated with infertility in males [36]. We have also added some biological hypotheses for these genes in the revised manuscript (Lines 181-186)

**Q8, L176-177 on page 7: "As expected, brain and pituitary showed the smallest number of DEGs, while skin and stomach had the largest (Fig. 3a).". It is unclear why it is expected?**

**AU:** Since the central neural system (e.g., Brain and pituitary) has been reported to evolve slowly across mammals [7], we thus expected a small number of DEGs that could be detected in such tissues between humans and cattle. In contrast, as the physiological and anatomical characteristics of skin and stomach are very distinct between humans and cattle, we thus expected a large number of DEGs that could be detected in these tissues between these two species. We have added this information in the revised manuscript (Lines 189-193).

**Q9, L179-183 on page 7: "an increased enrichment of active promoter-related states and decreased enrichment of repression-related states in humans when compared to their orthologous genes in cattle, and vice versa for cattle up-regulated DEGs). Please show what is the "active promoter-related states" and "repression-related states" in both figures and the main text.**

**AU**: Active promoter-related states include strongly active promoters (TssA) and transcribed at gene (TxFlnk), while repression-related states include Bivalent/poised TSS (TssBiv), Repressed polycomb (Repr) and Weak repressed polycomb (ReprWk). We have added this information in the revised manuscript (Lines 198-199).

**Q10, Fig.2b legend, the cutoff (log2 (fold-change) > 1.5; FDR < 0.05) was used to measure tissue-specific genes, it is not clear the power for the cutoff. Is there any reference? Similar to fig.3a legend, it is unclear the power of the cutoff of log2(fold-change, FC) > 1.2 and FDR < 0.05 to calculate the number of significantly up-regulated genes.**

**AU**: In the gene differential expression analysis, both multiple-test adjusted *P*-value and fold change (FC) are often considered simultaneously to identify significantly differentially expressed genes (DEGs) between conditions [37]. However, the thresholds for these two quantities depend much on the sample sizes, the underlying biology and other aspects of the experiment design. In general, the fold-change of 1.5 or 2 is often used as the cut-offs for identifying DEGs [38]. For instance, Tusher V G et al. identified DEGs with FC > 1.5 and FDR < 12% [39].Another previous study considered genes with FDR < 0.1 (without considering FC) as DEGs between species [6]. Here, we used log_2_(FC) > 1.5 & FDR < 0.05 to detect tissue-specific genes. In contrast, we used FC > 1.2 & FDR < 0.05 to identify genes differentially expressed between species, as the differences in gene expression are much bigger between tissues within species than within tissues between species. We have added this information in the revised manuscript (Lines 491-495).

**Reviewer #3:**

**The manuscript by Yuelin Yao and co-authors showcases a very comprehensive comparative functional genomics study between cattle and human populations. The topic falls within the scope of this journal and I believe this work could be relevant to both the readers specialized on mammalian functional genomics, and also to a wider audience with interest on advance methodology for the efficient and effective analysis of large-scale molecular biology datasets. In addition, the manuscript is exceptionally well written using a professional yet vibrant writing style allowing for an enjoyable reading. Because of these reasons, the manuscript deserves eventual publication in a journal the calibre of Genome Biology.**

**AU**: Thanks for such positive comments and compliments.

**Q1: One key concern is that I am not convinced the work has generated significantly new findings benefiting both human biomedicine and livestock genetic improvement. Put another way: What is the new insight that would've been missed by analysing each dataset set in isolation?**

**AU**: Although we have added additional analyses (e.g., functionally informed fine-mapping analysis) to support this conclusion (please see details in the responses to Q3-4 of reviewer #1), we still noted that comprehensive studies based on computational simulations and functional follow-ups would be required to fully address this, which we think is beyond the current study. We thus turned down this conclusion in the revised manuscript. By comparing human and cattle data, we could provide some insights into the evolutionary basis of complex trait genetics in humans and cattle, which will help borrow information across species in the future studies (Lines 101-104, in the revised manuscript).

**Q2: It has been long documented that most genes are either tissue-specific or ubiquitously expressed. Also, the kidney of a cow (for argument sake) is more similar to the kidney of a human than it is to the lung of the same cow. Therefore, the finding that "several human traits were significantly correlated with cattle traits across tissues" is no doubt interesting, but hardly ground-breaking.
I believe there is an overemphasis on (and an over-representation of) similarities, and the authors could've explored key physiological differences between cattle and human other than the obvious cornification and skin development (Fig. 5g). One obvious example could be the difference between the digestive systems of ruminants (cattle) vs monogastric (human). Another could be the fact that cattle do not sweat effectively and rely on respiration to cool themselves (and this could be link to the skin development mentioned earlier).**

**AU**: Thanks for these valuable comments and suggestions. We have highlighted the results on the difference between rumen of cattle *vs*. stomach of human in the revised manuscript. We detected 511 and 461 genes were up- and down-regulated in cattle rumen compared to human stomach. The up-regulated genes in cattle rumen were mainly enriched in multicellular organismal water homeostasis,cell-cell adhesion and tissue development, while the down-regulated genes were significantly enriched in digestion, response to topologically incorrect protein, response to endoplasmic reticulum stress and muscle contraction. In addition, we also highlighted the results on the difference in skin between humans and cattle. We detected 481 and 551 genes were up- and down-regulated in cattle skin compared to human skin. The up-regulated genes in cattle skin were mainly enriched in anatomical structure morphogenesis, vasculature development, blood vessel development and inflammatory response, while the down-regulated genes were significantly enriched in skin development, epidermis development, regulation of water loss via skin, establishment of skin barrier and keratinocyte differentiation.

We have added this information in the revised manuscript (Lines 209-223).

**Q3: Clearly from Fig. 6a and 6b, the enrichment for conserved genes is much higher than for diverged genes. I wonder if the reason for this over-representation of similarities is a bias generated by selecting only the top and bottom 10% DEG as the diverged and conserved genes, respectively. At the very least an MA-plot is warranted with cattle vs human differential expression on the y-axis is plotted against the average expression on the x-axis. Also, a further approach to generate new insight could make use of all the genes (not just the top and bottom 10%) and explore the relationship between differential expression and heritability estimates (separate LDSC and permutation for human and cattle). Then focussing on discordances, eg. high differential expression and low heritability.**

**AU**: We compared the heritability enrichment of genes defined by the top 5%, 10% and 20% of genes, and found consistent results. In addition, we found that the majority of conserved and diverged genes were different among tissues. We have provided the MA-plot for all 20 tissues as Fig. S5 in the revised manuscript. We have grouped all genes into 10 bins evenly according to their expression conservation in each of 20 tissues, and then tried to estimate the heritability enrichment for all these 200 gene groups together. However, due to the high overlap among these gene groups, the LDSC did not work well [40]. In the future study, more advanced methods are required for exploring this. Instead, we only investigated the heritability enrichment of 46 human traits for four groups of genes using LDSC, i.e., the top 10% (most diverged), 40%-50%, 50%-60%, bottom 10 % (most conserved), which were ordered by -log_10_FDR (from largest to smallest) from differential expression analysis between species. We found that genes with higher conserved expression showed higher enrichments for the heritability of complex traits (Figure 22a), and similar results were observed for gene co-expression (Figure 22b).

We have added this information in the revised manuscript (Lines 377-382, Fig. S21b).

**Figure 22. The heritability enrichments for four groups of genes with different conservation levels of gene expression and co-expression. a,** Genes are first ordered by -log_10_FDR (from largest to smallest) from differential expression analysis between species. The top 10% (most diverged), 40%-50%, 50%-60% and bottom 10 % (most conserved) of genes are selected in each of 20 tissues for the heritability enrichment analysis of 46 complex human traits using LDSC. **b**, Similar to **a** but genes are ordered by Corcor score (from the most diverged to the most conserved).

**Having said all that, I consider these concerns a minor defect, not a major flaw. With the proper explanation they are not even a defect. Indeed, I insist that the manuscript is a phenomenal piece of research and I look forward to seeing it published so that I (like many of my colleagues) can make use of its findings and the available datasets and scripts codes provided therein.**

**AU**: Thanks again for such positive comments.

**Reference:**

1.	Wang ZY, Leushkin E, Liechti A, Ovchinnikova S, Mossinger K, Bruning T, Rummel C, Grutzner F, Cardoso-Moreira M, Janich P, et al: **Transcriptome and translatome co-evolution in mammals.** *Nature* 2020, **588:**642-647.

2.	Breschi A, Gingeras TR, Guigo R: **Comparative transcriptomics in human and mouse.** *Nat Rev Genet* 2017, **18:**425-440.

3.	Lin S, Lin Y, Nery JR, Urich MA, Breschi A, Davis CA, Dobin A, Zaleski C, Beer MA, Chapman WC, et al: **Comparison of the transcriptional landscapes between human and mouse tissues.** *Proc Natl Acad Sci U S A* 2014, **111:**17224-17229.

4.	Zheng-Bradley X, Rung J, Parkinson H, Brazma A: **Large scale comparison of global gene expression patterns in human and mouse.** *Genome Biol* 2010, **11:**R124.

5.	Gerstein MB, Rozowsky J, Yan KK, Wang D, Cheng C, Brown JB, Davis CA, Hillier L, Sisu C, Li JJ, et al: **Comparative analysis of the transcriptome across distant species.** *Nature* 2014, **512:**445-448.

6.	Alam T, Agrawal S, Severin J, Young RS, Andersson R, Arner E, Hasegawa A, Lizio M, Ramilowski JA, Abugessaisa I, et al: **Comparative transcriptomics of primary cells in vertebrates.** *Genome Res* 2020, **30:**951-961.

7.	Gu X, Su Z: **Tissue-driven hypothesis of genomic evolution and sequence-expression correlations.** *Proc Natl Acad Sci U S A* 2007, **104:**2779-2784.

8.	Villar D, Berthelot C, Aldridge S, Rayner TF, Lukk M, Pignatelli M, Park TJ, Deaville R, Erichsen JT, Jasinska AJ, et al: **Enhancer evolution across 20 mammalian species.** *Cell* 2015, **160:**554-566.

9.	Berthelot C, Villar D, Horvath JE, Odom DT, Flicek P: **Complexity and conservation of regulatory landscapes underlie evolutionary resilience of mammalian gene expression.** *Nat Ecol Evol* 2018, **2:**152-163.

10.	Shibata Y, Sheffield NC, Fedrigo O, Babbitt CC, Wortham M, Tewari AK, London D, Song L, Lee BK, Iyer VR, et al: **Extensive evolutionary changes in regulatory element activity during human origins are associated with altered gene expression and positive selection.** *PLoS Genet* 2012, **8:**e1002789.

11.	Watanabe K, Taskesen E, van Bochoven A, Posthuma D: **Functional mapping and annotation of genetic associations with FUMA.** *Nat Commun* 2017, **8:**1826.

12.	Yengo L, Sidorenko J, Kemper KE, Zheng Z, Wood AR, Weedon MN, Frayling TM, Hirschhorn J, Yang J, Visscher PM, Consortium G: **Meta-analysis of genome-wide association studies for height and body mass index in approximately 700000 individuals of European ancestry.** *Hum Mol Genet* 2018, **27:**3641-3649.

13.	Weissbrod O, Hormozdiari F, Benner C, Cui R, Ulirsch J, Gazal S, Schoech AP, van de Geijn B, Reshef Y, Marquez-Luna C, et al: **Functionally informed fine-mapping and polygenic localization of complex trait heritability.** *Nat Genet* 2020, **52:**1355-1363.

14.	Jiang J, Cole JB, Freebern E, Da Y, VanRaden PM, Ma L: **Functional annotation and Bayesian fine-mapping reveals candidate genes for important agronomic traits in Holstein bulls.** *Commun Biol* 2019, **2:**212.

15.	Xiang R, Berg IVD, MacLeod IM, Hayes BJ, Prowse-Wilkins CP, Wang M, Bolormaa S, Liu Z, Rochfort SJ, Reich CM, et al: **Quantifying the contribution of sequence variants with regulatory and evolutionary significance to 34 bovine complex traits.** *Proc Natl Acad Sci U S A* 2019, **116:**19398-19408.

16.	Lee YS, Shin D, Lee W, Taye M, Cho K, Park KD, Kim H: **The Prediction of the Expected Current Selection Coefficient of Single Nucleotide Polymorphism Associated with Holstein Milk Yield, Fat and Protein Contents.** *Asian-Australas J Anim Sci* 2016, **29:**36-42.

17.	Yang J, Bakshi A, Zhu Z, Hemani G, Vinkhuyzen AA, Lee SH, Robinson MR, Perry JR, Nolte IM, van Vliet-Ostaptchouk JV, et al: **Genetic variance estimation with imputed variants finds negligible missing heritability for human height and body mass index.** *Nat Genet* 2015, **47:**1114-1120.

18.	Fang L, Sahana G, Ma P, Su G, Yu Y, Zhang S, Lund MS, Sorensen P: **Exploring the genetic architecture and improving genomic prediction accuracy for mastitis and milk production traits in dairy cattle by mapping variants to hepatic transcriptomic regions responsive to intra-mammary infection.** *Genet Sel Evol* 2017, **49:**44.

19.	Jiang J, Ma L, Prakapenka D, VanRaden PM, Cole JB, Da Y: **A Large-Scale Genome-Wide Association Study in U.S. Holstein Cattle.** *Front Genet* 2019, **10:**412.

20.	Subramanian S: **Deleterious protein-coding variants in diverse cattle breeds of the world.** *Genet Sel Evol* 2021, **53:**80.

21.	Hall SJ: **Effective population sizes in cattle, sheep, horses, pigs and goats estimated from census and herdbook data.** *Animal* 2016, **10:**1778-1785.

22.	Shao Y, Zhao FQ: **Emerging evidence of the physiological role of hypoxia in mammary development and lactation.** *J Anim Sci Biotechnol* 2014, **5:**9.

23.	Schröder MJA: **Effect of oxygen on the keeping quality of milk: I. Oxidized flavour development and oxygen uptake in milk in relation to oxygen availability.** *Journal of Dairy Research* 1982, **49:**407-424.

24.	Mayer SJ, Waterman AE, Keen PM, Craven N, Bourne FJ: **Oxygen concentration in milk of healthy and mastitic cows and implications of low oxygen tension for the killing of Staphylococcus aureus by bovine neutrophils.** *Journal of Dairy Research* 1988, **55:**513-519.

25.	Pennisi R, Salvi D, Brandi V, Angelini R, Ascenzi P, Polticelli F: **Molecular Evolution of Alternative Oxidase Proteins: A Phylogenetic and Structure Modeling Approach.** *J Mol Evol* 2016, **82:**207-218.

26.	Cvijovic I, Good BH, Desai MM: **The Effect of Strong Purifying Selection on Genetic Diversity.** *Genetics* 2018, **209:**1235-1278.

27.	Jordan IK, Rogozin IB, Wolf YI, Koonin EV: **Essential genes are more evolutionarily conserved than are nonessential genes in bacteria.** *Genome Res* 2002, **12:**962-968.

28.	Hess K, Oliverio R, Nguyen P, Le D, Ellis J, Kdeiss B, Ord S, Chalkia D, Nikolaidis N: **Concurrent action of purifying selection and gene conversion results in extreme conservation of the major stress-inducible Hsp70 genes in mammals.** *Sci Rep* 2018, **8:**5082.

29.	Consortium GT: **Human genomics. The Genotype-Tissue Expression (GTEx) pilot analysis: multitissue gene regulation in humans.** *Science* 2015, **348:**648-660.

30.	Wilks C, Zheng SC, Chen FY, Charles R, Solomon B, Ling JP, Imada EL, Zhang D, Joseph L, Leek JT, et al: **recount3: summaries and queries for large-scale RNA-seq expression and splicing.** *Genome Biol* 2021, **22:**323.

31.	Baldwin CL, Telfer JC: **The bovine model for elucidating the role of gammadelta T cells in controlling infectious diseases of importance to cattle and humans.** *Mol Immunol* 2015, **66:**35-47.

32.	Hein WR, Griebel PJ: **A road less travelled: large animal models in immunological research.** *Nat Rev Immunol* 2003, **3:**79-84.

33.	Mascarello F, Sacchetto R: **Structural study of skeletal muscle fibres in healthy and pseudomyotonia affected cattle.** *Ann Anat* 2016, **207:**21-26.

34.	Langfelder P, Horvath S: **WGCNA: an R package for weighted correlation network analysis.** *BMC Bioinformatics* 2008, **9:**559.

35.	Cope L, Zhong X, Garrett E, Parmigiani G: **MergeMaid: R tools for merging and cross-study validation of gene expression data.** *Stat Appl Genet Mol Biol* 2004, **3:**Article29.

36.	Li Q, Qiao D, Song NH, Ding Y, Wang ZJ, Yang J, Wang W, Yin CJ: **Association of DAZ1/DAZ2 deletion with spermatogenic impairment and male infertility in the South Chinese population.** *World J Urol* 2013, **31:**1403-1409.

37.	Love MI, Huber W, Anders S: **Moderated estimation of fold change and dispersion for RNA-seq data with DESeq2.** *Genome Biol* 2014, **15:**550.

38.	Thomas JG, Olson JM, Tapscott SJ, Zhao LP: **An efficient and robust statistical modeling approach to discover differentially expressed genes using genomic expression profiles.** *Genome Research* 2001, **11:**1227-1236.

39.	Tusher VG, Tibshirani R, Chu G: **Significance analysis of microarrays applied to the ionizing radiation response.** *Proc Natl Acad Sci U S A* 2001, **98:**5116-5121.

40.	Finucane HK, Bulik-Sullivan B, Gusev A, Trynka G, Reshef Y, Loh PR, Anttila V, Xu H, Zang C, Farh K, et al: **Partitioning heritability by functional annotation using genome-wide association summary statistics.** *Nat Genet* 2015, **47:**1228-1235.

**Second round of review**

**Reviewer 1**

The authors have provided additional analysis and results to address the concerns raised in the last round. However, there are still a number of issues, including unaddressed previous comments, some inconsistencies in the analyses, missing or disorganized tables/figures, lacking clarity in descriptions, especially in the methodology of analyses and the discussion section lacking depth.

My comments are specified in the following:

I appreciate the author correcting their heritability analysis. In the revised manuscript the author stated that ‘Compared to GREML-LDMS models, the sum-based permutation test based on the summary statistics was computationally faster, which could be used to scan a large number of genomic features in a short time [18].’ I disagree with this statement. Unless there is solid evidence including simulations to prove the equivalence of sum-based permutation test to GREML in estimating variance components. GREML-LDMS or LDSC is a method to estimate proportions of variance explained but the sum-based permutation test does not do this task. And you only test 3 traits for the comparison between GREML-LDMS the sum-based permutation test and 1/3 the correlation between the 2 estimates is not significant. what about the rest of 30+ traits? In supplementary figures, there is still mixed-up use of results not from variance components methods.

Although on the relative scale, conserved genes seem to be more enriched with h2 than divergent genes, the total amount of h2 explained is not great in either humans or cattle. I think the authors need to make this clear and provide a discussion on this point in the manuscript. Also, the sequence-level conservation based on dN/dS is only between humans and cattle. Therefore, such 2-species conservation is not very powerful, compared to the conservation between 30 mammals (https://www.ncbi.nlm.nih.gov/pmc/articles/PMC4626285/) or 100 vertebrates (https://www.pnas.org/doi/10.1073/pnas.1904159116). Thus, the results that human-cattle sequence-level conserved genes show less enrichment of h2 than transcriptome-conserved genes could be somewhat expected. The authors should clearly state this caveat in the results and provide discussions on this. Otherwise the authors can provide additional analysis to compare how the enrichment of heritability is different between transcriptome-conserved gene and sequence-level conserved sites across 30 mammals/100 vertebrates, instead of conserved between just human and cattle, to get to the bottom of the issue.

Accordingly, the statement ‘Finucane et al. reported that the heritability of complex traits was enriched in genomic regions with constrained DNA sequence across species [33]. We demonstrate that among orthologous genes (i.e., sequence conserved), transcriptionally conserved genes had significantly higher enrichment for the heritability of complex traits than diverged genes in humans and cattle.’ is a bit misleading. What Finucane et al found is ‘with 2.6% of SNPs explaining an estimated 35% of SNP-heritability on average across traits’. Xiang et al found 2.2% of conserved SNPs explain ~40% of heritability. The current study found on average slightly less than 10% of SNPs explain a bit more than 10% of heritability. therefore, in terms of heritability enrichment, the current study can not compare to previous findings. This needs to be clearly stated and discussed in the manuscript.

Fig. S17. Comparison of MAF and LD of SNPs in conserved and diverged genes. it appears at least in panel d appears to show that conserved genes have higher LD than divergent ones. When comparing MAF between groups, because of LD, I think it’s best to prune SNPs first and then use pruned SNPs to compare average MAF between groups.

In author response ‘As suggested by the reviewer, we further compared the gene expression profiles of horns and rumen (the RNA-seq data from tails in cattle were not available) in cattle to those of all 54 tissues in human GTEx (Figure 5-6). We found that, in general, cattle horns showed a low correlation (mean Pearson’s r = 0.37) of gene expression across all human tissues, while among them Fallopian Tube was the most similar one (Figure 5, Pearson’s r = 0.62). On the other hand, we found that cattle rumen showed the highest similarity with vagina, esophagus and skin in humans compared to other tissues, which was due to the high enrichment of epithelial cells in these tissues (Figure 6).’ I cant find any specific information regarding rumen and horn’s expression related to humans. Please provide/specify detailed figures/tables.

Authors kept mentioning the results from of PolyFun + SuSiE, but there is no methods description regarding how the models are constructed. Also I cant see the code of doing these analyses. They also mentioned PheWAS but there is not a description of this method.

Please specify the heritability model (LDMS) used in the paper, i.e., what fixed effects and random effects are used. the description of the approach in methods is ‘3-component GREML-LDMS model’. Does this mean only 3 GRMs are fitted? What about those ‘200-kb segmentation stratification of SNPs in four sets by LD scores of individual SNPs in R followed by GRM estimation’? were those LD and MAF stratified GRMs fitted together with GRMs made of SNPs within conserved, diverged and the rest of genome’ as well?

The authors have further checked their results and in the response letter stated that ‘However, we did not find a significant heritability enrichment correlation between human height and cattle stature across these 20 tissues.’ Many published works state the genomic conservation of this trait between humans and cattle. This point seems not addressed in the revised manuscript. Given that this finding appears to be in contrast to several published works, I think this may need some further investigations/discussions. Is it because the tissues enriched with variants affecting height are different between 2 species? Is it because the data quality/power of GWAS/RNA-seq of cattle are not good enough, compared to what's in humans? Maybe it’s a fact that human height is very different from cattle stature, but in any case, I think some more evidence/discussions are needed to support this disagreement with published results.

The author stated that ‘Our previous estimation showed that the power of GWAS (N ~ 300K) was similar between this Holstein bull population and the large cow population analysed previously [19] (https://www.frontiersin.org/articles/10.3389/fgene.2019.00412/full).’ I failed to find evidence in [19] to support that the GWAS power of 28,000 bulls is equivalent to the GWAS power of 300K cows. Can the authors show where exactly the evidence is and show statistical evidence of how the power was calculated?

In all analyses involving using statistical models in the manuscript, please specify the detailed mathematical equation used, what are the fixed effects and random effects fitted, and label these equations in numbers sequentially. For any claims made in the manuscript, please provide rigorous statistical support.

In author responses, ‘As shown in Figure 11, although sequence-conserved genes showed the highest enrichment (denoted as the triangle) for 11 out of 47 traits (e.g., weight and years of education), expression-conserved genes in particular tissues showed the highest enrichment for the remaining 36 traits.’ there is no figure 11 in main text and Fig. S11. Describes coefficient of variation in gene expression.

‘Thus, we have turned down this conclusion in the revised manuscript. We have also compared sequence-conserved genes and expression-conserved ones in terms of the heritability enrichment of complex traits and found that expression-conserved genes in certain tissues showed higher enrichment for relevant traits than sequence-conserved genes (Figure 11-12 and Figure 13a).’ I think the figures in the main text stopped at 8th and supplementary figures don’t fit the descriptions so I am not able to evaluate these responses.

Line 59-60: please clearly state genes considered are one-to-one orthologs and how many of them are analysed in both species.

Line 64-65: instead of using ‘large-scale’ please specify the average N of individuals used in each species across traits.

Line 96: ‘different’ instead of ‘unusual’, to cattle, humans can be unusual too

Line 97: LD should be high instead of large

Line 99-101: ‘millions of 100 highly accurate phenotypic records, including fertility, health and growth traits, have 101 been collected for cattle [7].’ Reference 7 describes some GWAS data from 28,000 cattle, where exactly are those millions of records?

**Reviewer 2**

The authors have done lots of good staff to address my and other reviewer's conserns. Their reponse and revision are fine to me. I do not have further questions. Thank you!

**Reviewer 3**

In their revised version (GBIO-D-20-01992R1), the authors have done a superb job at addressing my original concerns. Coming from a livestock genetics and genomics background, I particularly appreciate the introductory explanation around the population structure LD-driven differences between humans and cattle (lines 95-105). I agree these LD-driven differences can explain higher enrichment of cis-eQTLs around TSS in humans than in cattle (lines 272-278). I have no further comments.

**Authors’ response to reviewers**

Response to reviewers’ comments:

Reviewer #1:

General comments: The authors have provided additional analysis and results to address the concerns raised in the last round. However, there are still a number of issues, including unaddressed previous comments, some inconsistencies in the analyses, missing or disorganized tables/figures, lacking clarity in descriptions, especially in the methodology of analyses and the discussion section lacking depth.

AU: We have modified the manuscript carefully according to these comments and suggestions. Please see the detailed point-by-point responses to them below.

Q1. I appreciate the author correcting their heritability analysis. In the revised manuscript the author stated that ‘Compared to GREML-LDMS models, the sum-based permutation test based on the summary statistics was computationally faster, which could be used to scan a large number of genomic features in a short time [18].’ I disagree with this statement. Unless there is solid evidence including simulations to prove the equivalence of sum-based permutation test to GREML in estimating variance components. GREML-LDMS or LDSC is a method to estimate proportions of variance explained but the sum-based permutation test does not do this task. And you only test 3 traits for the comparison between GREML-LDMS the sum-based permutation test and 1/3 the correlation between the 2 estimates is not significant. what about the rest of 30+ traits? In supplementary figures, there is still mixed-up use of results not from variance components methods.

AU: We agree with the reviewer that GREML-LDMS or LDSC is a method to estimate proportions of variance explained but the sum-based permutation test does not do this task. Instead, the sum-based permutation test just does the GWAS signal enrichment analysis. In fact, we did the multi-component GREML-LDMS analysis for all the available cattle traits, but the models for the majority of them could not converge properly across many tissues, mainly due to the variance components being estimated were close to zero. Therefore, we only chose three milk production traits to compare these two methods, where most of models converged properly across tissues. We clearly stated, and rephrased the statement accordingly in the revised manuscript.

Q2. Although on the relative scale, conserved genes seem to be more enriched with h2 than divergent genes, the total amount of h2 explained is not great in either humans or cattle. I think the authors need to make this clear and provide a discussion on this point in the manuscript.

AU: We have carefully discussed this point in the revised Discussion as below:

Although on the relative scale, conserved genes seem to be more enriched with heritability than divergent genes, the total amount of heritability explained by conserved genes is not great in either humans or cattle on average across tissues. However, the top tissue for a complex trait could explain a relatively high proportion of heritability. For instance, 8% of SNPs in blood expression-conserved genes could explain 31% and 33% of heritability for inflammatory bowel disease and systemic lupus erythematosus, respectively (Additional file 9: Table S8). This finding suggested that expression-conserved genes contribute to the heritability of complex traits at a tissue-specific manner.

Q3. Also, the sequence-level conservation based on dN/dS is only between humans and cattle. Therefore, such 2-species conservation is not very powerful, compared to the conservation between 30 mammals (https://www.ncbi.nlm.nih.gov/pmc/articles/PMC4626285/) or 100 vertebrates (https://www.pnas.org/doi/10.1073/pnas.1904159116). Thus, the results that human-cattle sequence-level conserved genes show less enrichment of h2 than transcriptome-conserved genes could be somewhat expected. The authors should clearly state this caveat in the results and provide discussions on this. Otherwise the authors can provide additional analysis to compare how the enrichment of heritability is different between transcriptome-conserved gene and sequence-level conserved sites across 30 mammals/100 vertebrates, instead of conserved between just human and cattle, to get to the bottom of the issue.

AU: As suggested by the reviewer, we have clearly stated and discussed on this point in the revised manuscript. In addition, we ranked genes based on their average PhastCons scores (from largest to smallest), which represent the conservation scores for sequence alignments of 99 vertebrate genomes with human [1], and considered the top 10% genes (n=1731) as the sequence-level conserved sites across 100 vertebrates. We then conducted the heritability enrichment analysis of 47 complex traits for both sequence-conserved and expression-conserved genes. We still found that expression-conserved genes in certain tissues showed higher heritability enrichments than sequence-conserved ones (the revised Fig. 8a and Fig. S21a).

Q4. Accordingly, the statement ‘Finucane et al. reported that the heritability of complex traits was enriched in genomic regions with constrained DNA sequence across species [33]. We demonstrate that among orthologous genes (i.e., sequence conserved), transcriptionally conserved genes had significantly higher enrichment for the heritability of complex traits than diverged genes in humans and cattle.’ is a bit misleading. What Finucane et al found is ‘with 2.6% of SNPs explaining an estimated 35% of SNP-heritability on average across traits’. Xiang et al found 2.2% of conserved SNPs explain ~40% of heritability. The current study found on average slightly less than 10% of SNPs explain a bit more than 10% of heritability. therefore, in terms of heritability enrichment, the current study can not compare to previous findings. This needs to be clearly stated and discussed in the manuscript.

AU: As suggested by the reviewer, we have clearly added and discussed this point in the revised Discussion. As summarized in Table S8, we found that expression-conserved genes of the top tissue for a complex trait could explain a relatively high proportion of heritability. For instance, 8% of SNPs in blood expression-conserved genes could explain 31% and 33% of heritability for inflammatory bowel disease and systemic lupus erythematosus, respectively (Table S8). Compared to previous studies (i.e., Finucane et al., and Xiang et al.,), we found a relatively lower enrichment of heritability in expression-conserved genes than sequence-conserved regions. This may be due to the previous studies considered the sequence-conserved regions in the entire genome, including both genic and intergenic regions, whereas we here only focused on orthologous genes between humans and cattle.

Q5. Fig. S17. Comparison of MAF and LD of SNPs in conserved and diverged genes. it appears at least in panel d appears to show that conserved genes have higher LD than divergent ones. When comparing MAF between groups, because of LD, I think it’s best to prune SNPs first and then use pruned SNPs to compare average MAF between groups.

AU: As suggested by the reviewer, we obtained LD-independent SNPs using plink (v1.9) (--indep pairwise 1000 5 0.5), and then compared MAF of these SNPs between conserved and diverged genes across tissues. We still found that there was no significant difference in MAF between these two groups of genes across tissues. Therefore, MAF and LD might not bias the results. We have updated the results with LD-independent SNPs in the revised manuscript (Fig.S17a-b).

Q6. In author response ‘As suggested by the reviewer, we further compared the gene expression profiles of horns and rumen (the RNA-seq data from tails in cattle were not available) in cattle to those of all 54 tissues in human GTEx (Figure 5-6). We found that, in general, cattle horns showed a low correlation (mean Pearson’s r = 0.37) of gene expression across all human tissues, while among them Fallopian Tube was the most similar one (Figure 5, Pearson’s r = 0.62). On the other hand, we found that cattle rumen showed the highest similarity with vagina, esophagus and skin in humans compared to other tissues, which was due to the high enrichment of epithelial cells in these tissues (Figure 6).’ I cant find any specific information regarding rumen and horn’s expression related to humans. Please provide/specify detailed figures/tables.

AU: In the previous version, we just provided the figures in the response letter. As suggested by the reviewer, we here have provided these two plots as Additional file 2: Fig. S23a-b, and discussed them in the revised Discussion.

Q7. Authors kept mentioning the results from of PolyFun + SuSiE, but there is no methods description regarding how the models are constructed. Also I cant see the code of doing these analyses. They also mentioned PheWAS but there is not a description of this method.

AU: We have described PolyFun [2] + SuSiE [3] and PheWAS [4] carefully in the revised manuscript, and provided codes of doing them in the Github (https://github.com/B160389-2019/Comparative-Project). In brief, PolyFun [2] is an extension of S-LDSC that computes SNP prior causal probabilities via the same statistical framework (Step 1). These prior causal probabilities were then used as priors in SuSiE [3] for the following fine-mapping (Step 2) analysis of GWAS summary statistics. To investigate the association of a gene/variant with a variety of complex traits, we performed phenome-wide association analysis using PheWAS (https://atlas.ctglab.nl) [4], which includes a total of 4,756 GWAS. Only GWAS traits with Bonferroni corrected P-value < 0.05 were displayed in the PheWAS plots.

Q8. Please specify the heritability model (LDMS) used in the paper, i.e., what fixed effects and random effects are used. the description of the approach in methods is ‘3-component GREML-LDMS model’. Does this mean only 3 GRMs are fitted? What about those ‘200-kb segmentation stratification of SNPs in four sets by LD scores of individual SNPs in R followed by GRM estimation’? were those LD and MAF stratified GRMs fitted together with GRMs made of SNPs within conserved, diverged and the rest of genome’ as well?

AU: As suggested by the reviewer, we have clearly provided the details of LDMS in the revised manuscript. We first computed the amount of genetic variance in three milk production traits (i.e., milk, fat and protein yield), which had the largest population size (n = 27K) and the highest reliability of phenotypes [5], explained by SNPs within conserved and divergent genes using the 3-component GREML-LDMS model:

= µ + gcon + gdiv + grest + ;

where was the vector of phenotypes of individuals being analysed. The phenotypes were deregressed transmitting ability, i.e., the additive genetic values of cattle after correcting for all the known fixed effects. µ is global mean, gcon was the vector of polygenic effects for SNPs within conserved genes, where gcon~N(0, con2 ), con was the genomic relationship matrix (GRM) calculated by SNPs within conserved genes; gdiv was the vector of polygenic effects for SNPs within diverged variants, where gdiv~N(0, div2 ), div was the GRM calculated by SNPs within diverged genes; grest was the vector of polygenic effects for the rest of SNPs, where grest~N(0, rest2 ), rest was the GRM calculated by the rest variants; div was the incidence matrix made of the left variants set; e was the vector of residual. We applied GREML in GCTA [6] to calculate the heritability of each trait, ℎcon2 and ℎdiv2, respectively. For each group, the per-variant ℎ2 was calculated as the ℎ2 divided by the number of SNPs in the corresponding group.

Q9. The authors have further checked their results and in the response letter stated that ‘However, we did not find a significant heritability enrichment correlation between human height and cattle stature across these 20 tissues.’ Many published works state the genomic conservation of this trait between humans and cattle. This point seems not addressed in the revised manuscript. Given that this finding appears to be in contrast to several published works, I think this may need some further investigations/discussions. Is it because the tissues enriched with variants affecting height are different between 2 species? Is it because the data quality/power of GWAS/RNA-seq of cattle are not good enough, compared to what's in humans? Maybe it’s a fact that human height is very different from cattle stature, but in any case, I think some more evidence/discussions are needed to support this disagreement with published results.

AU: In the previous version, we have noticed that that it was very difficult to interpret the correlation of heritability enrichment between human and cattle traits across tissues, mainly due to the small number (n = 20) of tissues being analysed, the heterogeneity of complex traits between both species, and difference in the GWAS power and RNA-seq quality between both species. Therefore, we removed the heritability enrichment correlation part from the manuscript.

Q10. The author stated that ‘Our previous estimation showed that the power of GWAS (N ~ 300K) was similar between this Holstein bull population and the large cow population analysed previously [19] (https://www.frontiersin.org/articles/10.3389/fgene.2019.00412/full).’ I failed to find evidence in [19] to support that the GWAS power of 28,000 bulls is equivalent to the GWAS power of 300K cows. Can the authors show where exactly the evidence is and show statistical evidence of how the power was calculated?

AU: The phenotypes of bulls we analysed were DE-regressed PTA (breeding values), which were calculated based on millions of phenotypic records from the relatives (e.g., their daughters). For example, the 27K bulls had records for milk production from over 31.6 million daughters. We claim a similar power between 27K bulls and 300K cows mostly based on the empirical evidence that the smallest p-values for common QTLs in the two studies are comparable. We can provide additional evidence based on reliability of phenotype data on bulls and cows. The amount of information provided by a bull is equivalent to 5 cows for a highly heritable traits or 25 cows for a lowly heritable traits. Therefore, 27K bulls can be comparable to 300K cows depending on the heritability of traits and other factors

[5, 7, 8]. In addition, the genotypes for 300K cows have not been imputed to the whole genome sequence level, which the GWAS power may also be reduced due to the low LD between SNPs and causal variants.

Q11. In all analyses involving using statistical models in the manuscript, please specify the detailed mathematical equation used, what are the fixed effects and random effects fitted, and label these equations in numbers sequentially. For any claims made in the manuscript, please provide rigorous statistical support.

AU: We have added the mathematical equations used where necessary, and clearly described the fixed and random effects fitted. However, for some of methods (e.g., S-LDSC [9, 10] and PolyFun [2]), the mathematics behind them are too complicated to be fully understood by non-statisticians, thus beyond the scope of the current manuscript. In addition, these methods have been fully described in the original paper, which we carefully cited in the manuscript for avoiding the just copy and paste of those complex mathematics.

Q12. In author responses, ‘As shown in Figure 11, although sequence-conserved genes showed the highest enrichment (denoted as the triangle) for 11 out of 47 traits (e.g., weight and years of education), expression-conserved genes in particular tissues showed the highest enrichment for the remaining 36 traits.’ there is no figure 11 in main text and Fig. S11. Describes coefficient of variation in gene expression.

AU: We have summarized these results in Fig. 8a in the manuscript. The coefficient of variation (CV) in gene expression is the ratio of the standard deviation of gene expression to its mean value across individuals. The higher the CV, the greater the inter-individual variation of expression of a gene.

Q13. ‘Thus, we have turned down this conclusion in the revised manuscript. We have also compared sequence-conserved genes and expression-conserved ones in terms of the heritability enrichment of complex traits and found that expression-conserved genes in certain tissues showed higher enrichment for relevant traits than sequence-conserved genes (Figure 11-12 and Figure 13a).’ I think the figures in the main text stopped at 8th and supplementary figures don’t fit the descriptions so I am not able to evaluate these responses.

AU: Actually, we have provided these plots as Fig.8a and Additional file 2: Fig. S20-21 in the manuscript. In the previous version, we also provided these plots in the response letter labelled as Figure 11-12 and Figure 13a for the easy-review. Sorry for the confusion.

Minor comments:

Q14. Line 59-60: please clearly state genes considered are one-to-one orthologs and how many of them are analysed in both species.

AU: Done. A total of 17,315 one-to-one orthologous genes were analysed.

Q15. Line 64-65: instead of using ‘large-scale’ please specify the average N of individuals used in each species across traits.

AU: Done.

Q16. Line 96: ‘different’ instead of ‘unusual’, to cattle, humans can be unusual too

AU: Done.

Q17. Line 97: LD should be high instead of large

AU: Done.

Q18. Line 99-101: ‘millions of 100 highly accurate phenotypic records, including fertility, health and growth traits, have 101 been collected for cattle [7].’ Reference 7 describes some GWAS data from 28,000 cattle, where exactly are those millions of records?

AU: This has been described in the methods of previous publication by Jiang et al., [7]. This can also be found in the website of Council of Dairy Cattle Breeding

(CDCB) via the link below: https://www.uscdcb.com/wp-content/uploads/2021/10/2021-Activity-Report_101921_lowres.pdf

Reviewer #2:

General comments: The authors have done lots of good staff to address my and other reviewer's concerns. Their response and revision are fine to me. I do not have further questions. Thank you!

AU: Thank you for the kind word.

Reviewer #3:

General comments: In their revised version (GBIO-D-20-01992R1), the authors have done a superb job at addressing my original concerns. Coming from a livestock genetics and genomics background, I particularly appreciate the introductory explanation around the population structure LD-driven differences between humans and cattle (lines 95-105). I agree these LD-driven differences can explain higher enrichment of cis-eQTLs around TSS in humans than in cattle (lines 272-278). I have no further comments.

AU: Thank you for the kind word.

References

1.	Siepel A, Bejerano G, Pedersen JS, Hinrichs AS, Hou M, Rosenbloom K, Clawson H, Spieth J, Hillier LW, Richards S, et al: Evolutionarily conserved elements in vertebrate, insect, worm, and yeast genomes. Genome Res 2005, 15:1034-1050.

2.	Weissbrod O, Hormozdiari F, Benner C, Cui R, Ulirsch J, Gazal S, Schoech AP, van de Geijn B, Reshef Y, Marquez-Luna C, et al: Functionally informed fine-mapping and polygenic localization of complex trait heritability. Nat Genet 2020, 52:1355-1363.

3.	Wang G, Sarkar A, Carbonetto P, Stephens M: A simple new approach to variable selection in regression, with application to genetic fine mapping. Journal of the Royal Statistical Society: Series B (Statistical Methodology) 2020, 82:1273-1300.

4.	Watanabe K, Stringer S, Frei O, Umicevic Mirkov M, de Leeuw C, Polderman TJC, van der Sluis S, Andreassen OA, Neale BM, Posthuma D: A global overview of pleiotropy and genetic architecture in complex traits. Nat Genet 2019, 51:1339-1348.

5.	Jiang J, Cole JB, Freebern E, Da Y, VanRaden PM, Ma L: Functional annotation and Bayesian fine-mapping reveals candidate genes for important agronomic traits in Holstein bulls. Commun Biol 2019, 2:212.

6.	Yang J, Lee SH, Wray NR, Goddard ME, Visscher PM: GCTA-GREML accounts for linkage disequilibrium when estimating genetic variance from genome-wide SNPs. Proceedings of the National Academy of Sciences 2016, 113:E4579-E4580.

7.	Jiang J, Ma L, Prakapenka D, VanRaden PM, Cole JB, Da Y: A Large-Scale Genome-Wide Association Study in U.S. Holstein Cattle. Front Genet 2019, 10:412.

8.	VanRaden PM, Van Tassell CP, Wiggans GR, Sonstegard TS, Schnabel RD, Taylor JF, Schenkel FS: Invited review: reliability of genomic predictions for North American Holstein bulls. J Dairy Sci 2009, 92:16-24.

9.	Bulik-Sullivan BK, Loh PR, Finucane HK, Ripke S, Yang J, Schizophrenia Working Group of the Psychiatric Genomics C, Patterson N, Daly MJ, Price AL, Neale BM: LD Score regression distinguishes confounding from polygenicity in genome-wide association studies. Nat Genet 2015, 47:291-295.

10.	Finucane HK, Bulik-Sullivan B, Gusev A, Trynka G, Reshef Y, Loh PR, Anttila V, Xu H, Zang C, Farh K, et al: Partitioning heritability by functional annotation using genome-wide association summary statistics. Nat Genet 2015, 47:1228-1235.
